# Whole transcriptome profiling of Late-Onset Alzheimer’s Disease patients provides insights into the molecular changes involved in the disease

**DOI:** 10.1038/s41598-018-22701-2

**Published:** 2018-03-09

**Authors:** Anita Annese, Caterina Manzari, Claudia Lionetti, Ernesto Picardi, David S. Horner, Matteo Chiara, Mariano Francesco Caratozzolo, Apollonia Tullo, Bruno Fosso, Graziano Pesole, Anna Maria D’Erchia

**Affiliations:** 1 Institute of Biomembranes, Bioenergetics and Molecular Biotechnologies, National Research Council, Via Amendola 165/A, 70126 Bari, Italy; 2 Department of Biosciences, Biotechnology and Biopharmaceutics, University of Bari, Via Orabona 4, 70126 Bari, Italy; 3 Department of Biosciences, University of Milan, Via Celoria 26, 20133 Milan, Italy; 4 Center of Excellence in Comparative Genomics, University of Bari, Piazza Umberto I, 70121 Bari, Italy

## Abstract

Alzheimer’s Disease (AD) is the most common cause of dementia affecting the elderly population worldwide. We have performed a comprehensive transcriptome profiling of Late-Onset AD (LOAD) patients using second generation sequencing technologies, identifying 2,064 genes, 47 lncRNAs and 4 miRNAs whose expression is specifically deregulated in the hippocampal region of LOAD patients. Moreover, analyzing the hippocampal, temporal and frontal regions from the same LOAD patients, we identify specific sets of deregulated miRNAs for each region, and we confirm that the miR-132/212 cluster is deregulated in each of these regions in LOAD patients, consistent with these miRNAs playing a role in AD pathogenesis. Notably, a luciferase assay indicates that miR-184 is able to target the 3’UTR *NR4A2* - which is known to be involved in cognitive functions and long-term memory and whose expression levels are inversely correlated with those of miR-184 in the hippocampus. Finally, RNA editing analysis  reveals a general RNA editing decrease in LOAD hippocampus, with 14 recoding sites significantly and differentially edited in 11 genes. Our data underline specific transcriptional changes in LOAD brain and provide an important source of information for understanding the molecular changes characterizing LOAD progression.

## Introduction

Alzheimer’s Disease (AD) is the major chronic progressive form of senile dementia worldwide and is considered the prototypical neurodegenerative disease affecting the central nervous system (CNS). Characterized clinically by memory loss and deficits in cognitive domains^1^, it is distinguished by a long asymptomatic period that evolves into mild cognitive impairment (MCI) and later to dementia^2^. The neuropathology of AD is characterized by inflammation, oxidative stress and widespread neuronal loss. Pathological brain hallmarks are the presence of intraneuronal neurofibrillary tangles (NFTs) formed by twisted strands of hyperphosphorylated Tau, a microtubule-associated proteins (MAP), and of extracellular neuritic plaques containing β-Amyloid (Aβ) peptides, derived from proteolytic cleavage of the transmembrane glycoprotein Amyloid Precursor Protein (APP)^3^. These neuropathological changes originate in the entorhinal cortex and hippocampal formations, spreading later into other temporal, parietal, and finally frontal association cortices^4,5^. Distinct forms of AD sharing certain pathological and biochemical aspects have been described. Early-onset familial AD (EOFAD) accounts for less than 5% of all AD cases and is associated with Mendelian autosomal dominant inheritance of APP, PSEN1 and PSEN2 variants, while late-onset AD (LOAD) occurs sporadically and without any clear familial origin^6^. While some risk factors linked to LOAD pathogenesis have been identified (age^7^, family history^7^, social and cognitive engagement^7^, education^7^, history of traumatic brain injury (TBI)^8,9^, the ε4 allele of apolipoprotein E (APOE) gene^7^ and 16 other genomic loci identified through large-scale Whole Genome Association Studies (GWAS)^10^), the molecular mechanisms underlying neuronal dysfunction in LOAD remain elusive. Much effort has been focused on the “amyloid hypothesis” and devoted to finding molecular pathways involved in aberrant Aβ or Tau metabolism. Nevertheless, promising results from animal models tested with drugs that selectively targeted these pathways did not translate well in human clinical trials^11–13^. Increasing awareness of the complexity of gene expression regulation – including, but not restricted to - interactions between transcription factors, coding (mRNA) and non-coding (small and long) RNAs, and differential splicing patterns, led to proposals that changes in such mechanisms might be involved in the molecular pathogenesis of LOAD^14,15^. Here we present a comprehensive transcriptome profiling study, utilizing RNA-seq technology, that has allowed the identification of specific changes in the expression profiles of coding and non-coding RNAs (miRNA and lncRNAs) and in RNA editing levels in the hippocampus of LOAD patients, compared to cognitively normal controls and patients affected by Parkinson’s Disease (PD) - as neurodegenerative disease controls. Moreover, we have extended our miRNA profiling to the temporal and frontal areas of the brains of the same LOAD patients, identifying miRNAs whose deregulated expression could be related to LOAD progression. Our study thus sheds light on molecular mechanisms deregulated in the final stages of LOAD - identifying processes that could be valuable both in explaining the progression of the disease and in the rational design of therapeutic approaches.

## Results

### Transcriptomic Profiling of Hippocampus of LOAD patients

We used RNA-seq to examine the transcriptomic profile of the hippocampal CA1 region of a cohort of six patients affected by LOAD (samples AD1-6), six patients affected by PD (samples PD1–6 samples) and six cognitively normal controls (samples Ctrl1-6) (Table 1). All sampled individuals were Caucasian males with a comparable age of death, while LOAD patients had comparable dementia status (Braak V or VI). All frozen tissues preserved total RNA integrity as indicated by RIN values (5 to 8). PD patients were included as “disease-control patients”, to highlight transcriptomic changes that are LOAD specific, rather than common to general neurodegenerative processes. We chose the hippocampal region CA1 as the area of investigation for its relevance to memory processes and as this area is, together with the entorhinal cortex, the first affected by the pathogenic mechanisms associated with LOAD.

**Table 1:** Clinic-pathological information of the subjects analyzed by RNA-seq and miRNA-seq.

Case	ID	Gender	Race	Expired age	PMI (hrs)	Braak stage	Brain Bank	RIN
Ctrl1	5028	male	Ca	68	18	ND	NICHD	7.9
Ctrl2	5174	male	Ca	61	21	ND	NICHD	6.7
Ctrl3	5247	male	Ca	65	22	ND	NICHD	7.8
Ctrl4*	5352	male	Ca	81	17	ND	NICHD	6.1
Ctrl5	5533	male	Ca	67	23	ND	NICHD	5.5
Ctrl6	5362	male	Ca	60	16	ND	NICHD	5.5
AD1	1625	male	Ca	70	1	V	NICHD	5.2
AD2*	4737	male	Ca	80	7	VI	NICHD	7.6
AD3	5195	male	Ca	81	9	VI	NICHD	5.2
AD4	5198	male	Ca	72	5	V	NICHD	7.2
AD5	1946	male	Ca	69	5	VI	NICHD	5.8
AD6	A313/06	male	Ca	76	22	VI	LNDBB	5.7
PD1	1272	male	Ca	79	14	ND	NICHD	5.5
PD2	1741	male	Ca	72	20	ND	NICHD	7.1
PD3	1901	male	Ca	75	3	ND	NICHD	5.3
PD4	4526	male	Ca	79	1	ND	NICHD	6.8
PD5	5329	male	Ca	82	18	ND	NICHD	5.9
PD6	5520	male	Ca	63	8	ND	NICHD	5.7

Case: sample ID assigned in the present study; ID: sample ID in the original Bank; Ctrl: Non-Demented Control; AD: LOAD Patient; PD: Parkinson’s Disease Patient; race: Ca = Caucasian; PMI: Post-Mortem Interval expressed in hours; Braak stage: index used to classify the degree of AD pathology; ND: Not defined; NICHD: Brain and Tissue Bank for Developmental Disorders; LNDBB: London Neurodegenerative Diseases Brain Bank; RIN: RNA integrity number of total RNA preparation. (*) Outlier samples according to PCA analysis of gene expression data.

Directional RNA sequencing of rRNA-depleted total RNA generated an average of ∼140 million reads per sample, of which 88 to 92% could be aligned to the reference genome, suggesting excellent coverage and sequencing depth with low ribosomal RNA contamination (Supplementary Table S1). RNA-seq data analysis identified 25,272 annotated genes as expressed in, at least, one of the sequenced samples. Principal Component Analysis (PCA) indicated anomalous behavior for Ctrl4 and AD2 samples, which were thus discarded from all further analyses (Supplementary Figure S1), while no outlier was observed among PD samples.

CuffDiff2^16^ identified a total of 2,122 genes as differentially expressed (p-value ≤ 0.05 after false discovery rate correction) between LOAD samples and controls, of which 2,075 were protein coding genes and 47 were lncRNAs (of which 23 lincRNAs). Of the 2,075 differentially expressed protein coding genes, 789 were upregulated and 1,286 were downregulated, and of the 47 differentially expressed lncRNAs, 19 were upregulated (of which 11 lincRNAs) and 28 were downregulated (of which 12 lincRNAs) in LOAD samples. The complete list of deregulated genes is reported in Supplementary Table S2. Nineteen protein-coding genes were found to be differentially expressed between hippocampal PD samples and controls (Supplementary Table S3). Eleven of these genes (*ANKRD22, HAMP, HLA-DRA, HSPA6, CD14, FCGBP, HSPB1, HSPA7, BAG3, SERPINH1* and *TNFRSF1B*) were also present in the list of the 2,075 protein coding genes deregulated in LOAD patients. We analyzed these shared deregulated genes using the STRING tool^17,18^, and noted that they were involved in neurodegenerative and inflammatory pathways such as defense response, response to unfolded proteins, positive regulation of immune response and toll-like receptor (TLR) signaling. Accordingly, the common LOAD and PD differentially expressed protein coding genes were removed from the initial list of 2,075 LOAD deregulated genes and, in order to determine which pathways are affected in LOAD patients, the resulting 2,064 genes that were differentially expressed in a LOAD-specific manner, were analyzed using the IPA tool^19^. As shown in Table 2, we found that activities related to the regulation of important neurological functions, including synaptic long-term potentiation, neuronal signaling, axonal guidance signaling and mitochondrial dysfunction, were significantly enriched among this differentially expressed gene set. Interestingly, we noted that the products of several genes among this set are involved in pathways regulated by the miR-132/212 cluster, known to be down-regulated in AD^20–26^. In particular, we found the up-regulation of some direct targets of miR-132, such as *ITPKB* (log2FC: 1.33, padj: 0.0024), involved in Tau phosphorylation^27^, *TLR6* (log2FC: 1.36, padj: 0.0024)*, IL6R* (log2FC: 1.37, padj: 0.0014) and *IRAK3* (log2FC: 1.33, padj: 0.0048), involved in inflammatory signaling^28^. Moreover, we found *BDNF*, a positive upstream regulator of miR-132/212 transcription^29^, to be down-regulated (log2FC: −2.6, padj: 0.0041) as was *RASAL1*, which regulates angiogenesis through mir-132^30^ (log2FC: −1.46, padj: 0.0014) (Supplementary Table S2).

**Table 2:** IPA pathway analysis of the deregulated protein coding genes in LOAD hippocampus.

Ingenuity Pathways	−log(p-value)
Neuropathic Pain Signaling in Dorsal Horn Neurons	6.2E + 00
Synaptic Long Term Potentiation	5.8E + 00
nNOS Signaling in Neurons	5.6E + 00
Mitochondrial Dysfunction	5.6E + 00
GABA Receptor Signaling	5.4E + 00
Calcium Signaling	5.3E + 00
Dopamine-DARPP32 Feedback in cAMP Signaling	4.8E + 00
Role of Pattern Recognition Receptors in Recognition of Bacteria and Viruses	4.1E + 00
CREB Signaling in Neurons	3.4E + 00
Synaptic Long Term Depression	3.4E + 00
Axonal Guidance Signaling	3.2E + 00
Huntington’s Disease Signaling	2.2E + 00
Amyotrophic Lateral Sclerosis Signaling	1.7E + 00
ERK/MAPK Signaling	1.6E + 00
Neuroprotective Role of THOP1 in Alzheimer’s Disease	1.5E + 00
Induction of Apoptosis by HIV1	1.4E + 00
PI3K/AKT Signaling	1.3E + 00
Reelin Signaling in Neurons	1.3E + 00
Semaphorin Signaling in Neurons	1.0E + 00
Dopamine Receptor Signaling	1.0E + 00
Agrin Interactions at Neuromuscular Junction	8.6E-01
Apoptosis Signaling	6.8E-01
Dopamine Degradation	6.3E-01
Pathogenesis of Multiple Sclerosis	5.5E-01
Parkinson’s Signaling	4.6E-01
Neurotrophin/TRK Signaling	4.4E-01
Myc Mediated Apoptosis Signaling	4.4E-01
STAT3 Pathway	4.3E-01
Death Receptor Signaling	4.1E-01
Retinoic acid Mediated Apoptosis Signaling	3.4E-01
Noradrenaline and Adrenaline Degradation	2.8E-01
Melatonin Degradation I	2.7E-01
Serotonin Degradation	2.5E-01

The Fisher-exact Test P-value is reported in each column.

**Figure 1:**
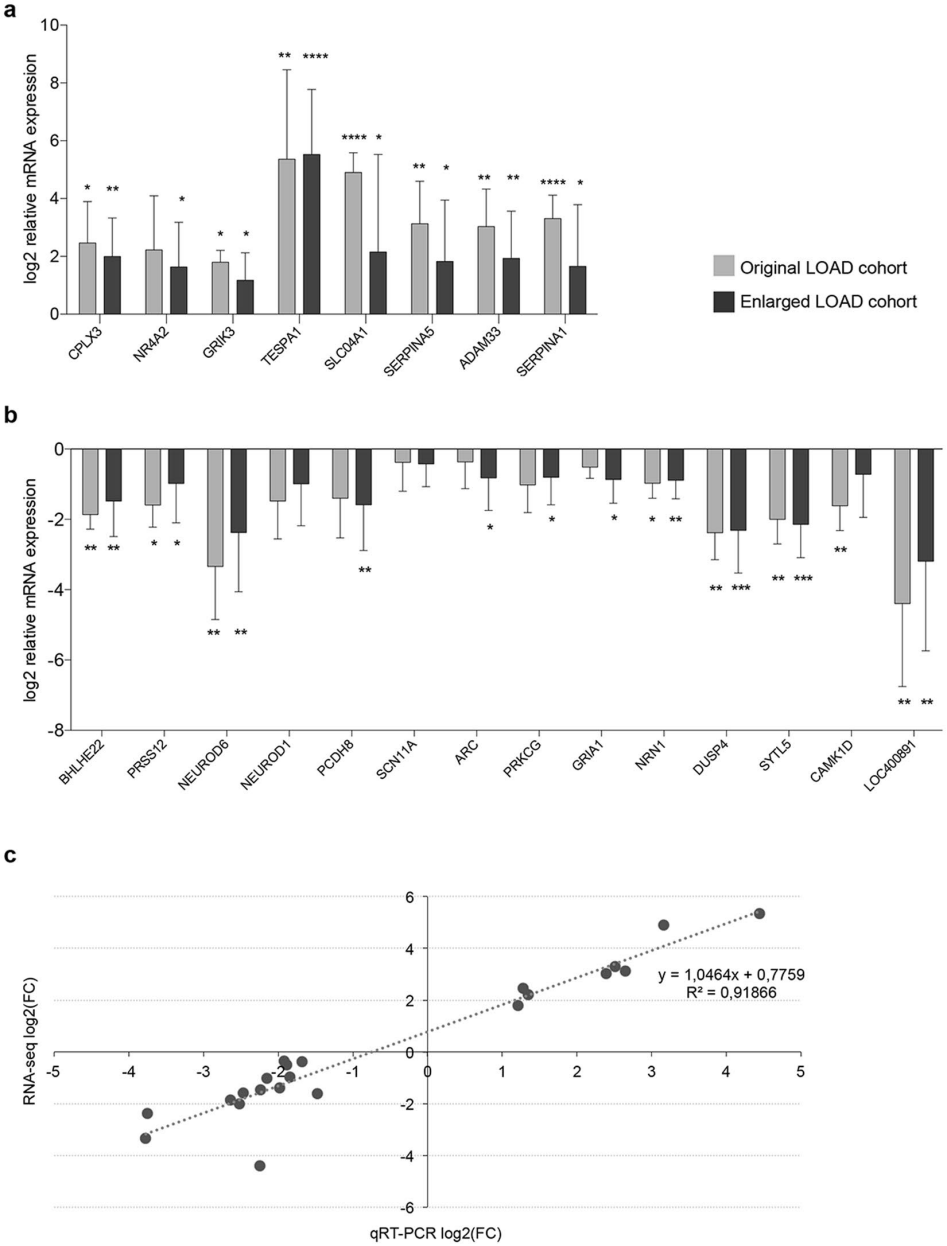
RT-qPCR validation of the RNA-seq dataset in LOAD hippocampus. Validation of 8 selected upregulated genes **(a)** and 13 selected downregulated protein coding genes and 1 downregulated lncRNA **(b)** in the original (5 LOAD patients and 5 control subjects) and in the enlarged sample group (9 LOAD patients and 9 control subjects). The data are expressed as the means of log2(ΔΔCt) ± SD. P-value was calculated by T-test. *p-value ≤ 0.05; **p value ≤ 0.01; ***p value ≤ 0.001; ****p value ≤ 0.0001. **(c)** Linear correlation between log2(FC) values computed by CuffDiff2 on RNA-seq data and log2(FC) values detected by qRT-PCR analysis for the 22 selected genes in the original (5 LOAD patients and 5 control subjects) sample group.

We validated the hippocampal RNA-seq differential expression data by RT-qPCR using an enlarged cohort of nine controls (five original controls plus four new controls) and nine LOAD patients (five original LOAD patients plus four new LOAD patients), chosen to fit criteria of selection of the original cohort used in the RNA-seq analysis. Given that the exclusion of Ctrl4 from RNA-seq analyses caused a reduction of the average age of control samples with respect to LOAD samples, we selected control samples with a higher average age than the original control groups (see Supplementary Table 4). From the list of deregulated genes, we selected 21 protein coding genes, implicated in neurological functions and showing brain-predominant expression and 1 lncRNA (Supplementary Table S5). RT-qPCR analysis confirmed the differential expression levels for all 8 up-regulated genes (*CPLX3, NR4A2, GRIK3, TESPA, SLCO4A1, SERPINA5, ADAM33, SERPINA1)*, both in the original and extended cohorts (p-value ≤ 0.05) (Fig. 1a and Supplementary Figure S2). Regarding the down-regulated genes, RT-qPCR analysis confirmed the expression trend resulting from RNA-seq data for all 14 genes (*BHLHE22, PRSS12, NEUROD6, PCDH8, NRN1, DUSP4*, *CAMK1D, NEUROD1, GRIA1, SYTL5, PRKCG, ARC, SCN11A* and *LOC400891*), in the original and/or in the extended cohort (p-value ≤ 0.05), although two genes (*NEUROD1* and *SCN11A*) did not show a statistically significant reduction of expression in the RT-qPCR analyses (Fig. 1b and Supplementary Figure S3). In addition, for all 22 deregulated genes, a positive and highly significant correlation was also found between the estimates of fold change in expression level from RNA-seq data and RT-qPCR results (R^2^: 0.9187) (Fig. 1c).

### MicroRNA Profiling in LOAD Brain regions

To gain insights into the complexity and specificity of transcriptomic changes in LOAD, we next extended our analysis to miRNA profiling. In order to identify miRNAs whose deregulation is related to the spatio-temporal progression of the disease, we analyzed three different vulnerable brain areas, the hippocampal region CA1, the middle temporal gyrus Brodmann’s Area 21 and the middle frontal gyrus Brodmann’s Area 46, in the same LOAD patients and controls used in the RNA-seq experiment. This choice was determined by existing evidence that these brain regions are involved in distinct stages of the neurodegenerative process in terms of development of the pathology and functional outcome, with the hippocampus affected first, followed by the temporal lobe and, finally, the frontal lobe^4,5^. In the hippocampal analysis, we also included PD patients, in order to identify shared deregulated miRNAs between LOAD and PD hippocampal samples.

Small RNA-seq generated an average of ∼4.5 millions of reads per sample, with 90% of bases showing Q scores above 30 (Supplementary Table S6). Mapping of reads from hippocampal samples identified 1,018 annotated miRNAs expressed in, at least, one of the sequenced samples. Considering a coverage of at least 10 reads/miRNA, this number was reduced to 407. Differential expression analysis identified 4 miRNAs (miR-184, miR-34c-3p, miR-375 and miR-132-5p), that were differentially expressed (all down-regulated) in LOAD patients respect to control samples (padj ≤ 0.05) (Table 3). In addition, 40 miRNAs were differentially expressed in hippocampal PD samples with respect to controls (padj ≤ 0.05) (11 up-regulated and 29 down-regulated) (Supplementary Table S7), but none of these were present in the list of deregulated LOAD hippocampal miRNAs. PD deregulated miRNA targets are enriched in some neuronal and inflammatory pathways also identified for LOAD miRNAs, consistent with the involvement of such pathways in general responses to brain injury (p-value ≤ 0.05) (Supplementary Table S8).

**Table 3:** Deregulated miRNA in hippocampus, temporal gyrus and frontal gyrus of LOAD patients, as revealed by miRNA-seq.

miRNA	log2FC	p-value	padj
***LOAD Hippocampus***			
miR-184	−4.26	1.1E-05	1.41E-03
miR-34c-3p	−3.31	4.7E-05	4.48E-03
miR-375	−1.77	1.1E-03	4.91E-02
miR-132-5p*	−1.04	8.8E-04	4.78E-02
***LOAD Temporal gyrus***			
miR-501–3p	2.13	5.1E-13	1.31E-10
miR-10a-5p	1.23	2.3E-04	1.25E-02
miR-320a	0.76	5.5E-04	2.33E-02
miR-28–3p	0.61	1.8E-05	1.40E-03
miR-30a-3p	0.51	2.2E-04	1.25E-02
miR-539–5p	−1.53	8.1E-05	2.64E-03
miR-132-5p*	−1.77	5.9E-12	2.26E-09
miR-132-3p*	−1.85	7.6E-11	1.16E-08
miR-212-5p*	−1.85	1.5E-10	1.40E-08
miR-212-3p*	−1.80	9.1E-11	1.16E-08
***LOAD Frontal gyrus***			
miR-941	0.80	5.3E-06	4.75E-04
miR-582-5p	−0.85	2.0E-03	4.41E-02
miR-889-3p	−1.27	1.4E-04	6.08E-03
miR-132-5p*	−0.91	4.8E-04	4.62E-02
miR-132-3p*	−1.58	6.6E-06	4.75E-04
miR-212-5p*	−1.34	1.0E-06	3.96E-04
miR-212-3p*	−1.99	5.7E-09	1.24E-06

Expression levels are expressed as log2 fold change; P-value and the corresponding corrected P-value (padj) were calculated by DESeq2. (*) Members of miR-132/212 family, already known to be down-regulated in AD^20–26^.

Mapping of reads from middle temporal gyrus samples identified 1,205 annotated miRNAs as expressed in, at least, one of the sequenced samples. Considering a coverage of at least 10 reads/miRNA, this number was reduced to 442. Differential expression analysis identified 10 miRNAs deregulated (padj ≤ 0.005) in LOAD patients. Five of these (miR-501-3p, miR-10a-5p, miR-320a, miR-28-3p and miR-30a-3p) were upregulated and 5 (miR-539-5p, miR-132-5p, miR-212-3p, miR-132-3p and miR-212-5p) were downregulated (Table 3).

1,212 annotated miRNAs were detected in, at least, one of the middle frontal gyrus samples. Considering a coverage of at least 10 reads/miRNA, this number fell to 445. Differential expression analysis identified 7 miRNAs as deregulated in LOAD samples of which 1 (miR-941) was up-regulated and 6 (miR-582-5p, miR-889-3p, miR-212-5p, miR-212-3p, miR-132-3p and miR-132-5p,) were down-regulated (padj ≤ 0.005) (Table 3).

KEGG pathway annotations using DIANA miRPath tool^31^ allowed us to identify 6 pathways that are enriched in genes with predicted targets for the miRNAs deregulated in every brain region studied (p-value ≤ 0.05). These pathways include MAPK and neurotrophin signaling, axon guidance, long-term potentiation, glutamatergic and cholinergic synapses (Table 4).

**Table 4:** Pathways identified by DIANA miRPath analysis, affected by the deregulated miRNAs from hippocampus, temporal and frontal gyrus of LOAD patients. The Fisher-exact test P-value is reported in each column.

KEGG Pathway	Hippocampus	Temporal Gyrus	Frontal Gyrus
Glutamatergic synapse	1.9E-07	2.7E-04	2.5E-07
MAPK signaling pathway	1.9E-07	5.9E-03	3.5E-05
Axon guidance	3.5E-04	4.4E-09	2.3E-05
Neurotrophin signaling pathway	1.7E-03	7.0E-09	2.5E-07
Long-term potentiation	1.9E-02	2.7E-08	4.2E-05
Cholinergic synapse	2.9E-02	1.8E-05	5.8E-03

miRNA profiling confirmed the down-regulation of the miR-132/212 family - previously identified as down-regulated in LOAD^20–26^ - in each of the three different regions of studied LOAD brains, consistent with a role in LOAD pathogenesis for these miRNAs and supporting the consistency of our analyses. No other miRNA showed differential expression in all three regions of LOAD brains, although the identified deregulated miRNAs of each region affected common pathways, involved in the regulation of different aspects of neuronal cellular function.

**Figure 2:**
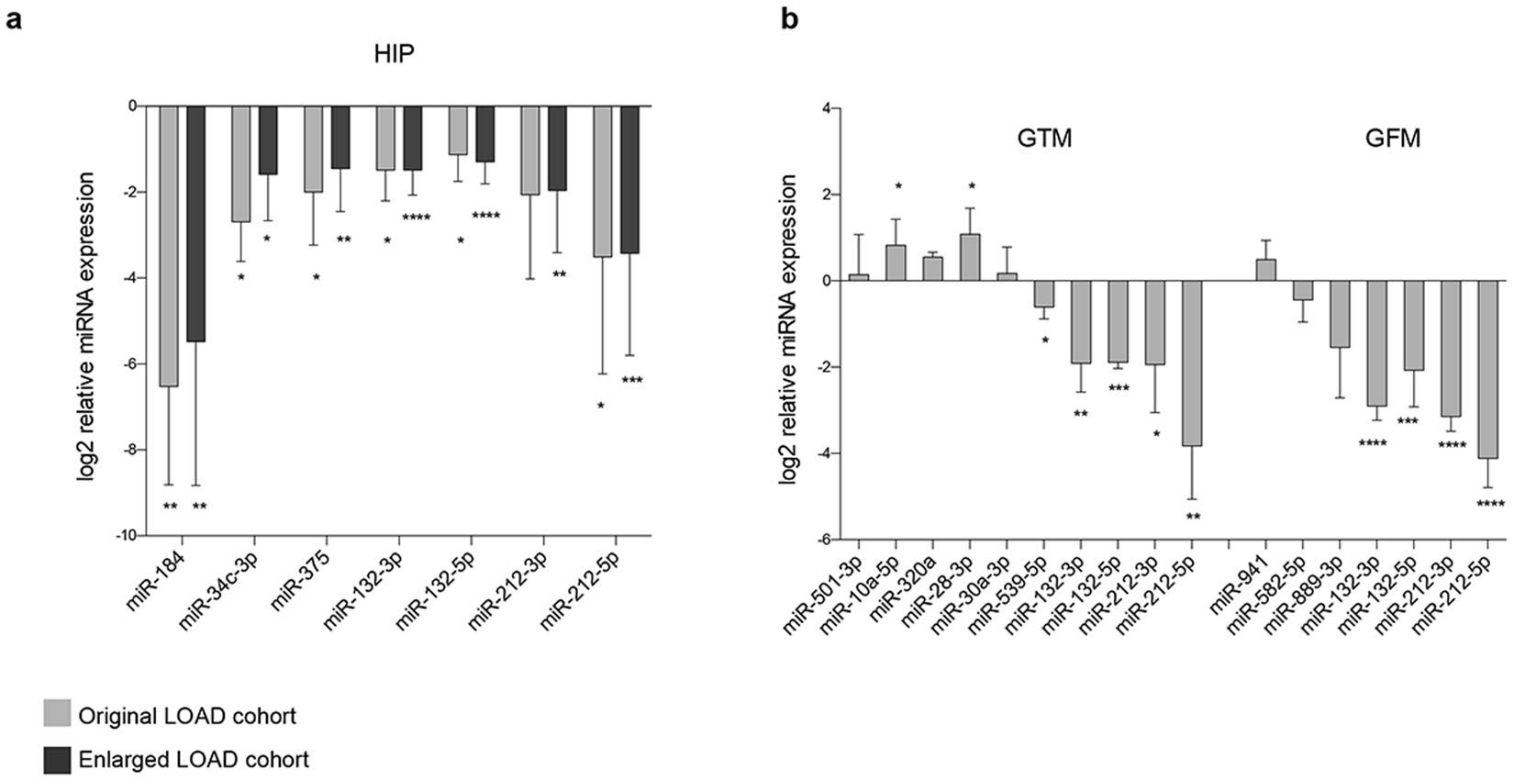
RT-qPCR validation of miRNAs expression in different LOAD brain regions. RT-qPCR assay confirmed miRNA deregulated expression in LOAD hippocampus (HIP) (**a**), middle temporal gyrus (GTM) and middle frontal gyrus (GFM) (**b**). In hippocampus, the deregulated expression of miR-184, miR-34c-3p, miR-375 and miR-132/212 cluster was confirmed in both the original (5 LOAD patients and 5 control subjects) and in the enlarged sample group (9 LOAD patients and 9 control subjects). The data are expressed as the means of log2(ΔΔCt) ± SD. P-value was calculated by T-test. *p-value ≤ 0.05; **p value ≤ 0.01; ***p value ≤ 0.001; ****p value ≤ 0.0001.

RT-qPCR assays were used to validate the deregulated miRNAs of each brain region. For the hippocampal region, we used both the original and the enlarged cohorts, as employed for the RNA-seq data validation (Table 1 and Supplementary Table S4). We confirmed the downregulation of all miRNAs identified in miRNA-seq (miR-184, miR-34c-3p, miR-375 and miR-132-5p), and also of the other members of the 132/212 cluster (p-value ≤ 0.05) (Fig. 2a and Supplementary Figure S4). For the middle temporal gyrus and middle frontal gyrus, we used the original cohort of 5 controls and 5 LOAD samples. In both brain regions, for all deregulated miRNAs, RT-qPCR assay confirmed the expression trend identified in miRNA-seq (in the middle temporal gyrus: p-value ≤ 0.05 for miR-10a-5p, miR-28-3p miR-539-5p and miR-132/212 cluster members; in the middle frontal gyrus: p-value ≤ 0.001 for miR-132/212 cluster members) (Fig. 2b and Supplementary Figure S4).

### miR-184 targets the 3’UTR of NR4A2 transcript

**Figure 3:**
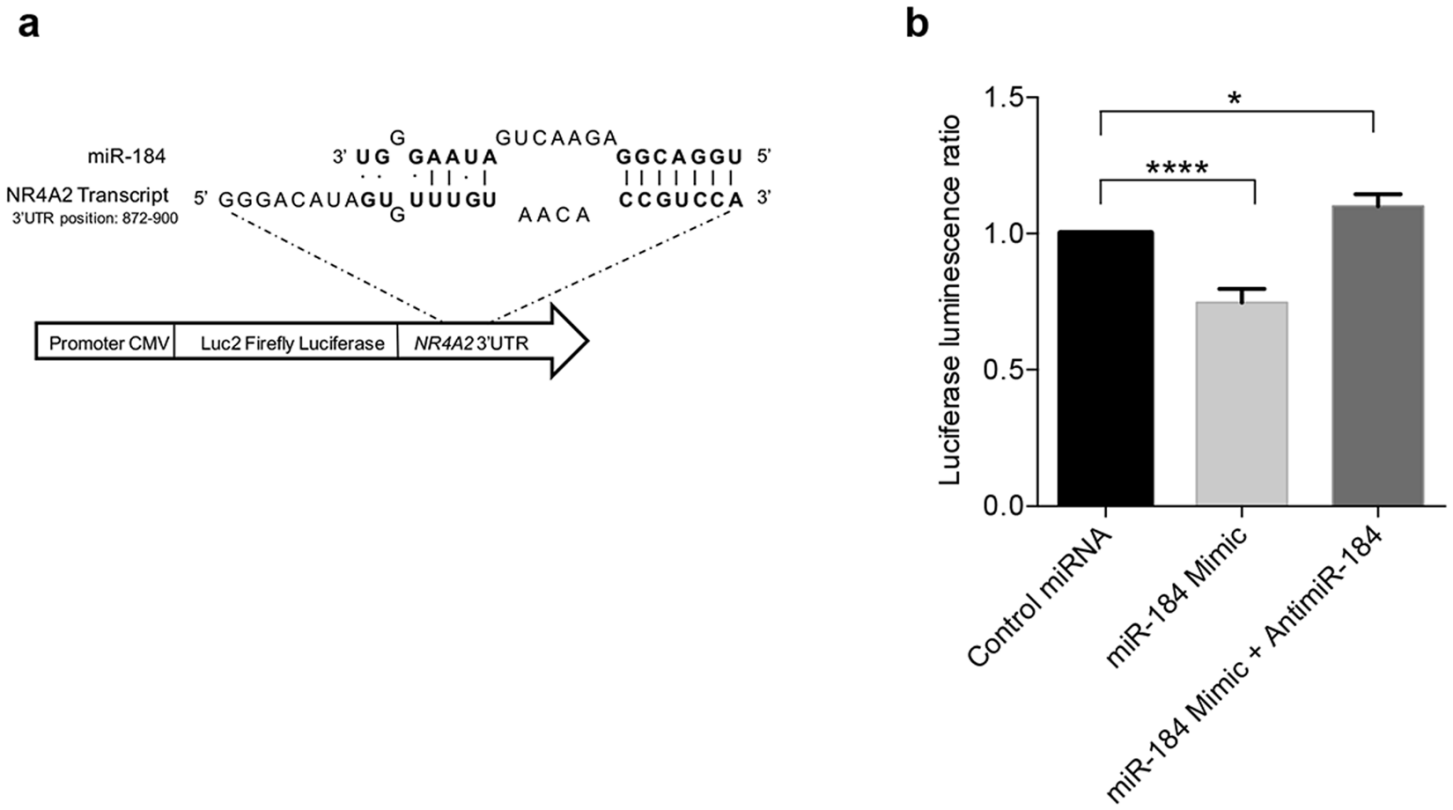
Experimental validation of miR-184/*NR4A2* interaction by luciferase assay. **(a)** Schematic representation of the 3’UTR of *NR4A2* transcript (NM_006186.3) cloned in the pMIR-reporter luciferase miRNA expression vector, downstream the firefly luciferase gene. The sequence alignment between miR-184 seed region and *NR4A2* 3’UTR is reported. **(b)** Luciferase assay. H1299 cells were transfected with a negative control miRNA mimic, miR-184 mimic alone and with anti-miR-184 along with pMIR luciferase reporter vectors containing *NR4A2* 3’UTR. Luciferase expression was normalized by Renilla expression and by calibrating the results data against the control sample (H1299 cells transfected with the control miRNA mimic). Data represent the means ± SD from the results of three independent experiments. P-value was calculated by T-test. *p-value ≤ 0.05; ****p-value < 0.0001.

We used the microRNA Target Filter, a microRNA target prioritization tool available within IPA^19^, the DIANA miRPath^31^ algorithm and Miranda^32^ to compare the deregulated miRNAs and transcripts identified in LOAD hippocampus. All tools predicted with high confidence, using TargetScan Human^33^ as source, the interaction between miR-184 and the *NR4A2* and *NRN1* transcripts. Interestingly, we found an inverse expression correlation for *NR4A2* and miR-184, as *NR4A2* was over-expressed (Fig. 1a and Supplementary Figure S2) and miR-184 was down-regulated (Fig. 2a and Supplementary Figure S4) in our RNA-seq data. We verified the predicted interaction miR-184/*NR4A2* using a luciferase reporter assay. We cloned the 3’ untranslated region (UTR) of the *NR4A2* transcript downstream of the *luc2* firefly luciferase open reading frame in the pMIR-Reporter Luciferase miRNA Expression Vector (Fig. 3a). The recombinant vector was transfected into H1299 cells either with a negative control miRNA, with the miR-184 mimic alone or together with a miR-184 inhibitor (anti-miR-184). We found that miR-184 caused a significant reduction, of about 25%, of the normalized luciferase expression with respect to the control miRNA, while the co-transfection of equimolar quantity of the miR-184 mimic and the antimiR-184 didn’t produce any significant difference with respect to the control miRNA (Fig. 3b). These results indicate that *NR4A2* transcript can be directly targeted by miR-184, consistent with the expression levels of *NR4A2* that we found in LOAD hippocampus. On the contrary, *NRN1* was down-regulated (Fig. 1a and Supplementary Figure S2) in our RNA-seq data, as miR-184. We also tested the predicted interaction miR-184/*NRN1* by luciferase reporter assay. We found that miR-184 did not produce any significant reduction in luciferase expression with respect to the control miRNA, thus excluding a *NRN1* regulation from miR-184 (Supplementary Figure S5b).

### RNA editing analysis

**Figure 4:**
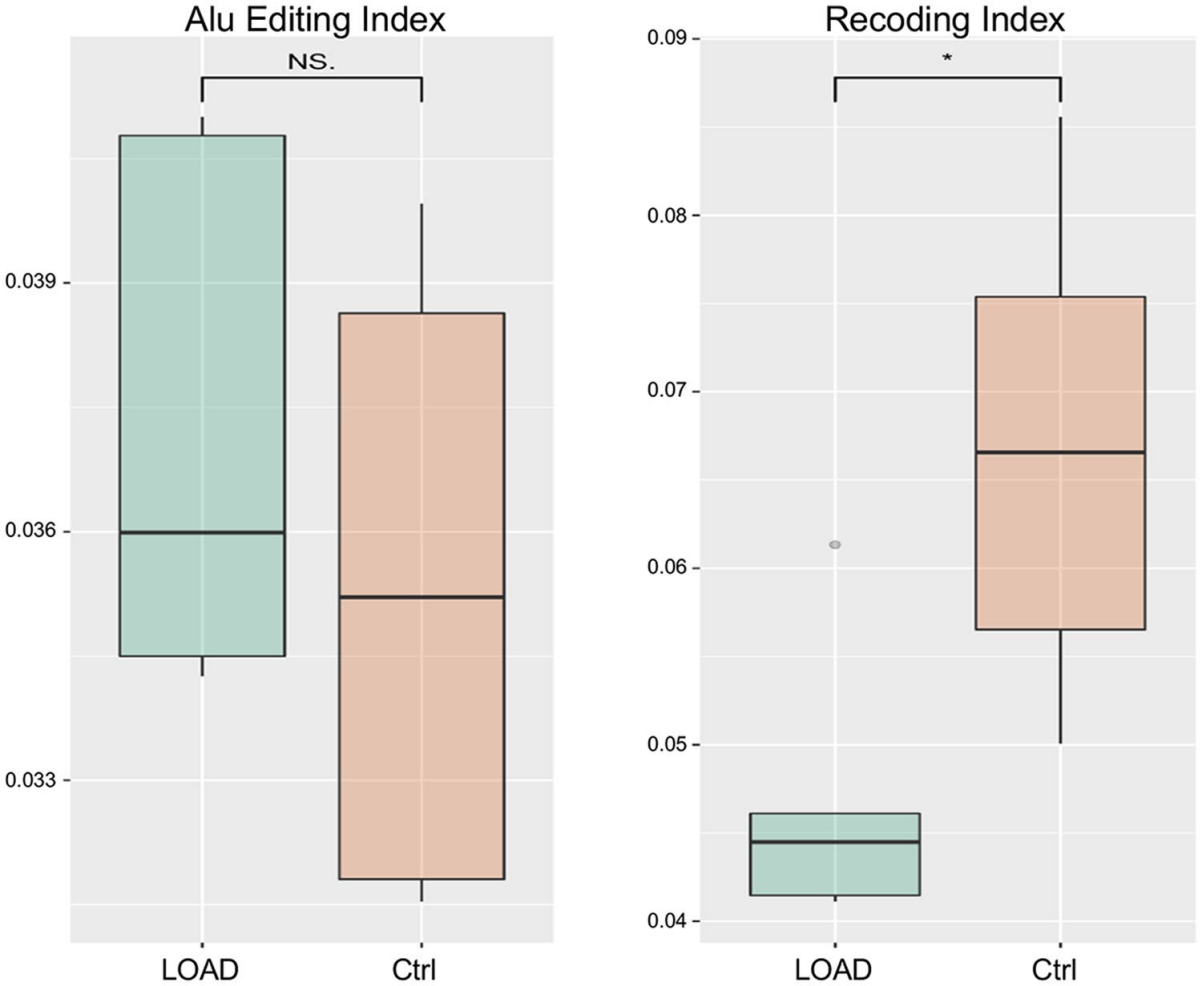
Average RNA editing levels expressed across Alu sequences and recoding sites. Global RNA editing activity calculated through the Alu Editing index and the Recoding Editing Index in LOAD hippocampus. Values for LOAD patients and controls are shown as box plots. P-value was calculated by t-test, followed by Benjamini-Hochberg procedure for multiple test p-values correction. *p-value ≤ 0.05. The box lower and upper limits correspond to the 25th and 75th percentile respectively and the line in the middle represents the median value (50th percentile). The whiskers lengths are inferred by using the following formula: (i) upper whisker = 75th percentile + 1.5*IQR; (ii) lower whisker = 25th percentile + 1.5*IQR, where IQR = 75th – 25th percentile. Dots correspond to outlier values.

**Figure 5:**
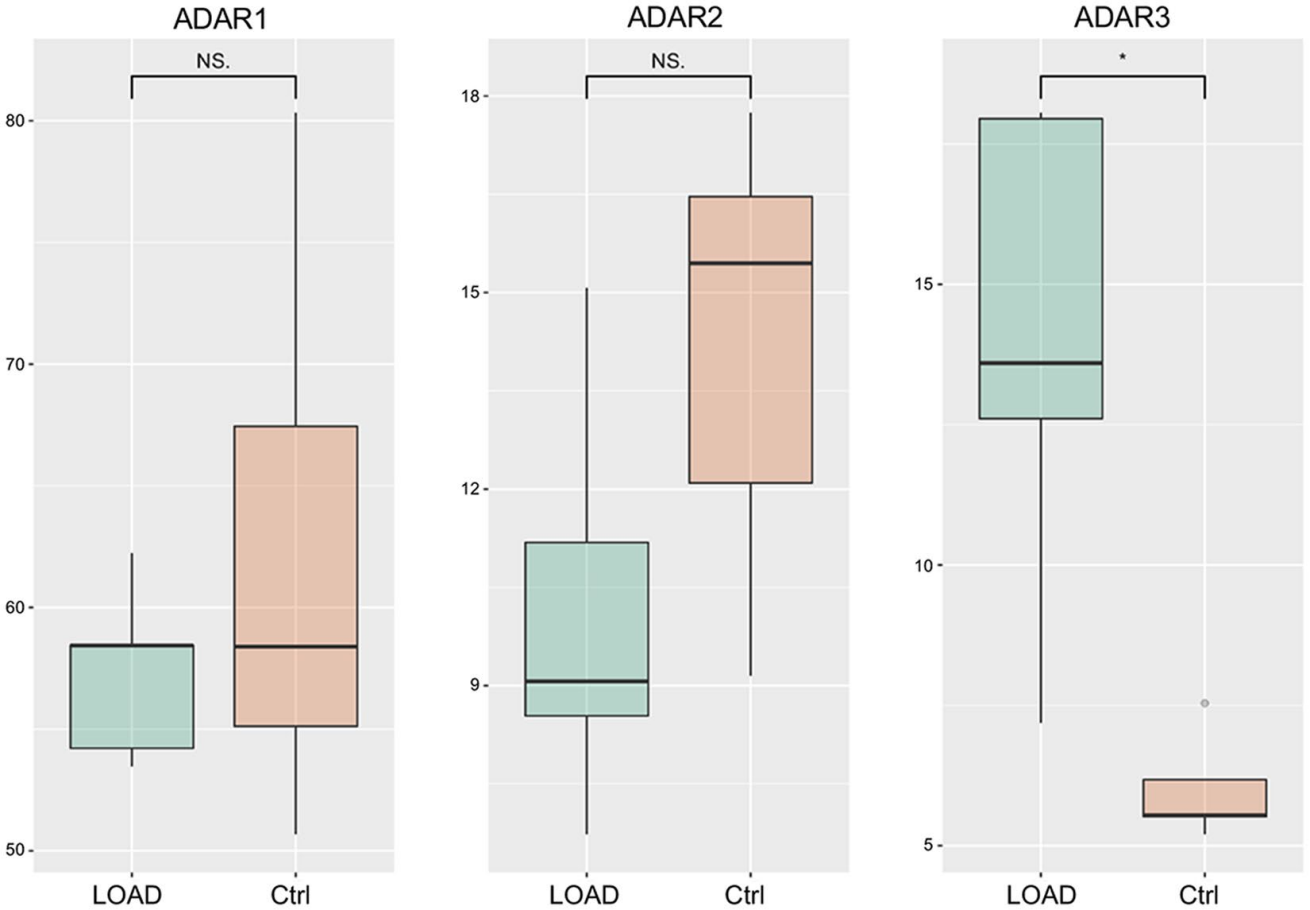
Expression of ADAR1, ADAR2 and ADAR3 genes. Expression levels of ADAR genes in LOAD hippocampal RNA-seq data calculated using CuffDiff2. FPKM values for LOAD patients and controls are shown as box plots. *p-value ≤ 0.05. The box lower and upper limits correspond to the 25th and 75th percentile respectively and the line in the middle represents the median value (50th percentile). The whiskers lengths are inferred by using the following formula: (i) upper whisker = 75th percentile + 1.5*IQR; (ii) lower whisker = 25th percentile + 1.5*IQR, where IQR = 75th – 25th percentile. Dots correspond to outlier values.

RNA editing is an important post-transcriptional process that alters the genetic blueprint of an organism by specific modifications in primary RNAs. In human, it mainly involves the deamination of adenosines to inosines by the family of adenosine deaminase acting on RNA (ADAR) enzymes acting on double RNA strands and its deregulation has been linked to a variety of neurological and neurodegenerative disorders^34,35^. We investigated A-to-I RNA editing alterations in LOAD hippocampal tissues using RNA-seq data. First, for each sample, we calculated the global editing activity through the Alu editing index (AEI) as it represents the weighted average editing level across all expressed Alu sequences^36^. We found a slight, but non-significant, increase of editing activity in LOAD samples compared to controls (Fig. 4). Interestingly, the RNA editing activity at recoding sites, that have a functional role in brain, appeared decreased in LOAD hippocampal samples (p-value < 0.05), as attested by the Recoding Editing Index (REI) calculated as the weighted average of editing levels over all known recoding sites from the REDIportal database^37^ (Fig. 4). Focusing on recoding editing sites, we found that 14 out of 1,585 sites were significantly and differentially edited (t-test followed by 10% FDR multiple-testing correction) in LOAD samples compared to controls (Table 5). In particular, we confirmed the deficient RNA editing at the glutamate receptor subunit B, GRIA2, Q/R site, previously described in LOAD hippocampus^38^ and at other five sites (COPA I/V site, GRIK1 Q/R site, GRIK2 I/V site, GRIA3 R/G site, GRIA4 R/G site), identified in a previous study that analyzed the editing levels at 180 recoding sites in different LOAD brain regions^39^. Finally, we evaluated the expression of ADAR genes in RNA-seq data. We found that ADAR1 and ADAR2 were not significantly differentially expressed in LOAD samples respect to controls although the ADAR2 expression level appeared slightly decreased in LOAD (Fig. 5). Interestingly, we found that the ADAR3 locus, encoding for an inactive adenosine deaminase protein, primarily expressed in the brain, was significantly over-expressed in LOAD samples (Fig. 5).

**Table 5:** Recoding sites with significant differential RNA editing levels in LOAD hippocampus. P-value was calculated by T-test and corrected using Benjamini-Hochberg with FDR = 0.1.

Gene name	Edit site location	AA change	Mean Ctrl	Mean LOAD	Edit difference (Ctrl-LOAD)	p-value
BLCAP	chr20:36147572	Y/C	0.122	0.181	−0.059	0.004
COPA	chr1:160302244	I/V	0.160	0.283	−0.123	0.029
GRIA2	chr4:158257875	Q/R	0.972	0.918	0.054	0.022
GRIA3	chrX:122598962	R/G	0.906	0.764	0.142	0.017
GRIA4	chr11:105804694	R/G	0.524	0.290	0.234	0.021
GRIK1	chr21:30953750	Q/R	0.601	0.393	0.207	0.004
GRIK2	chr6:102337689	I/V	0.372	0.211	0.160	0.027
GRIK2	chr6:102337702	Y/C	0.637	0.465	0.172	0.033
VN1R1	chr19:57967115	Y/C	0.058	0.134	−0.076	0.013
ZNF235	chr19:44793030	I/M	0.127	0.254	−0.127	0.001
ZNF235	chr19:44793302	R/G	0.049	0.113	−0.064	0.018
ZNF397	chr18:32825609	K/E	0.051	0.185	−0.135	0.008
ZNF397	chr18:32825654	I/V	0.065	0.218	−0.153	0.014
ZNF582	chr19:56896203	N/D	0.109	0.273	−0.164	0.020

## Discussion

LOAD is an age-related neurodegenerative disorder, for which whole transcriptome profiling represents an informative approach to characterize end-stage alterations resulting from interactions among multiple genetic, epigenetic and environmental factors. To date, several studies have investigated transcriptomic changes in LOAD, employing various technological platforms (RT-qPCR, microarray^40–43^, RNA-seq^15,44–46^), different tissue samples (transgenic AD models^47,48^, patient-derived cell lines^47,48^, post-mortem tissues^49^), different disease stages and brain areas, variable post-mortem interval (PMI) affecting RNA quality, and finally different bioinformatics protocols to analyze data. An accurate set of genes and pathways deregulated in LOAD is, thus, far from established. In an attempt to overcome these issues, we have performed, using NGS technology, a comprehensive transcriptomic profiling, including coding and non-coding RNAs (lncRNAs and miRNAs) of post-mortem hippocampal tissues from three cohorts, one consisting of LOAD patients, one consisting of cognitively normal controls and the last consisting of PD patients. To restrict the variability, samples were selected to minimize gender, ethnicity, age at death and disease stage differences between patients and controls and to have a PMI not longer than 23 hours. Indeed, it has been demonstrated that all categories of biomolecules remain stable up to 48 hours post-mortem^50^, consistent with RIN values of RNA obtained from all samples.

We used the ‘whole’ frozen brain tissue as laser-micro-dissection, targeting exclusively the neuronal soma, misses a variable proportion of transcripts that are transported for pre- or post-synaptic translation^51^. We used both grey and white matter since several studies, which examined grey and white matter separately, observed similar deregulated level of expression of genes involved in LOAD^40^. We included PD patients as “neuro-inflammatory disease” controls in order to identify LOAD specific transcriptomic changes, not shared with general neuro-inflammation processes. In fact, considering that affected regions also contain inflammatory cells, linked to neurodegeneration and not normally associated with healthy tissue (such as macrophages and T lymphocytes), the comparison involving the control group may involve tissues with a quite distinct cellular composition. Considering that our LOAD patients had high Braak score (advanced disease), to exclude that the differential expressed genes might be affected by an imbalance between the original cellular populations between LOAD and control samples, we analyzed also the expression level of some cell-type specific genes in our RNA-seq data. We did not find any statistically significant changes in the expression of neuronal markers: *DCX* (log2FC: −0.43, padj: 0.50), *RBFOX3* (log2FC: −0.37, padj: 0.46) and *TUB* (log2FC: −0.43, padj: 0.43); astroglial markers: *GFAP* (log2FC: 0.23, padj: 0.94), *AQP4* (log2FC: 0.74, padj: 0.52) and *SLC1A3* (log2FC: 0.59, padj: 0.37); microglial markers: *AIF1* (log2FC: 1.21, padj: 1) and *CX3CR1* (log2FC: −0.07, padj: 0.94). This observation indicates that LOAD tissues analyzed in our study preserved the original cellular population, and that the results of the differential analysis were not affected by differential expression of cell-type specific genes.

RNA-seq identified a set of 2,064 specific differentially expressed transcripts in the hippocampus of LOAD patients, which includes coding and long non-coding RNAs. The deregulated genes are enriched in roles related to the regulation of important neural processes, such as neurogenesis, synaptic vesicle trafficking, long-term potentiation, neurite outgrowth and hyper-phosphorylation of Tau protein, consistent with their involvement in LOAD pathogenesis. For the data validation by RT-qPCR, we enlarged both the control and the LOAD sample groups, including control samples with a higher average age with respect to the original control groups (Supplementary Table S4), as we were aware that, with the exclusion of Ctrl4 in the RNA-seq analysis, the ages of control samples became slightly smaller than those of LOAD samples. The deregulated expression of the selected group of transcripts was confirmed both in the original and in the enlarged cohorts, thus supporting the consistence of our bioinformatics analysis and suggesting that the difference of age between the two groups did not significantly affect our results.

In order to identify miRNAs differently expressed during LOAD neurodegenerative progression, with the aim of uncovering new diagnostic and prognostic markers for the disease as well as new potential drug targets, we extended the miRNA profiling from the hippocampus to the middle temporal gyrus (Brodmann’s Area 21) and middle frontal gyrus (Brodmann’s Area 46) of the same LOAD patients. These specific brain regions were chosen according to evidence that they are involved in distinct stages of the neurodegenerative process, with the hippocampus affected first, followed by the temporal lobe and at last the frontal lobe^4,5^. Our data further sustain the role of the miR-132/212 cluster in AD pathogenesis^52^, as its expression was deregulated in all three brain regions of the LOAD patients. Additionally, sequencing and RT-qPCR analyses demonstrate the differential expression of other specific miRNAs in each of the three brain regions considered, three miRNAs in the hippocampus, six miRNAs in middle temporal gyrus and three miRNAs in middle frontal gyrus of the same LOAD patients. These results suggest that miRNA expression changes in LOAD might be brain area specific and could differently contribute to disease progression.

Interplay between LOAD hippocampal deregulated genes and miRNAs was supported by *in silico* predictive tools that revealed a possible interaction between the *NR4A2* and *NRN1* transcripts and miR-184. Both *NRN1* and *NR4A2* predicted miRNA target sites can be considered canonical (7-8 nucleotides that match perfectly with miR-184 seed region) (Fig. 3a and Supplementary Figure S5a). Nevertheless, it is well known that many factors beyond pairing capacity determine functional interactions *in vivo* and, consequently, the presence of a putative miRNA binding site in a transcript is not sufficient to assert its functional relevance in the absence of experimental validation^53,54^. Accordingly, we performed luciferase assays that showed that miR-184 is able to target the 3’UTR of the *NR4A2* transcript but not of the *NRN1* transcript indicating that only *NR4A2* expression is regulated by miR-184 *in vivo* (Fig. 3b and Supplementary Figure S5b). In order to understand the different target specificity in the two transcripts, we considered the mammalian orthologs of *NR4A2* and *NRN1* genes. We observed higher conservation of the seed-matching region in *NR4A2* than in *NRN1* binding site, consistent with the observation that the conservation level of the seed matched site in 3′UTRs of orthologous genes is a relevant feature in predicting miRNA interaction^55^. Notably, our sequencing data recover an inverse correlation of expression between *NR4A2*, that was over-expressed, and miR-184, that was down-regulated in LOAD patients, while *NRN1*, like miR-184, was strongly down-regulated in LOAD patients.

MiR-184 was previously shown to trigger neural stem cell (NSC) proliferation and inhibit differentiation by repressing the NSC fate-regulator Numblike^56^. Its deregulation could negatively affect ongoing neurogenesis in the hippocampal neurogenic niches of adult brains, and which are known to be compromised in AD^57^. Several gene targets for miR-184 have been described, including mediators of neurological development and apoptosis, such as E2F1 and DP^58^.

*NR4A2*, also known as *Nurr1*, encodes an orphan nuclear receptor, belonging to the nuclear receptor subfamily 4A (NR4A), which also includes NR4A1 and NR4A3^59^. It is expressed in several brain areas and plays important roles in various neural processes including cognitive functions and/or long-term memory in forebrain areas and hippocampal formation. Immune-fluorescence staining with a specific NR4A2 antibody in 5XFAD mice showed that the NR4A2 protein was prominently localized in brain areas with Aβ plaque accumulation, highlighting a possible involvement of this protein in AD pathology^60^. The potential regulation of *NR4A2* by miR-184 requires further functional testing but leads us to suggest that the loss of expression of miR-184 in LOAD hippocampus may contribute to NR4A2 accumulation in Aβ plaques.

While the down-regulation of miR-132/212 cluster in AD is well established by the current study (Table 3 and Fig. 2) and by other published studies^21,22^, we also identified deregulation of several genes whose products are involved in miR-132/212 related pathways. *BDNF* (brain derived neurotrophic factor), which positively regulates miRNA-132/212 cluster transcription *via* the ERK1/2-dependent (but CREB-independent) pathway^29^ was down-regulated in LOAD brains. MiR-132 regulates Tau phosphorylation by targeting *ITPKB* (inositol-trisphosphate3-kinase B), which is recovered as up-regulated in LOAD brains in the current study. ITPKB is known to enhance both ERK1/2 kinases and BACE1 activity, and to be elevated in LOAD brains^27^. Finally, miR-132 is a negative regulator of inflammation^28^, and a positive regulator of vasculogenesis^30^. In our study, miR-132 down-regulation is correlated with the up-regulation of inflammatory signals (such as *TLR6, IL6R and IRAK3*) and the down-regulation of *RASAL1*, a positive effector of angiogenesis.

Finally, we investigated RNA editing levels in the LOAD hippocampus since alterations in RNA editing have been implicated in AD^39^ and in other neurodegenerative diseases^34,35^. We found a general decrease of RNA editing activity at recoding sites (Fig. 4), in agreement with a previous study that reported decreased editing levels in different regions of AD patients’ brains, analyzing 180 recoding sites by a targeted resequencing approach supplemented by a microfluidic-based high-throughput PCR coupled with next-generation sequencing^31^. We identified 14 recoding sites with significant and differential editing levels in LOAD hippocampal tissues compared to controls (Table 5). Among these sites, we found down-regulation of RNA editing levels in several glutamate receptors: GRIA2, GRIA3, GRIA4, GRIK1, GRIK2. Regarding the Q/R site of GRIA2, it has been demonstrated that editing of this site is essential in neurons, as incorporation of unedited GRIA2 subunits results in an increase in glutamate receptors that are permeable to calcium, leading to excitotoxicity and cell death of neurons^61^. Our finding of low RNA editing levels at the Q/R site of GRIA2 is in line with previous findings that reported this deficiency in the hippocampus of AD patients, suggesting that it may be an early event that precedes neuronal demise^38^. Three ADAR enzymes are present in mammals: ADAR (ADAR1), ADARB1 (ADAR2) and ADARB2 (ADAR3). ADAR1 and ADAR2 are the main catalytic enzymes, accountable for all A-to-I editing events, while ADAR3 has not been shown to have deaminase activity *in vitro* and has no known *in vivo* target^62^. Interestingly, while changes in ADAR1 and ADAR2 expression levels were not observed in our RNA-seq data, we recovered a significant increase of ADAR3 transcript levels in LOAD hippocampal tissues (Fig. 5). Recently, it has been reported that ADAR3 is able to bind directly to *GRIA2* pre-mRNA transcripts, thus competing with ADAR2 activity and inhibiting the RNA editing at the Q/R site of GRIA2. This mechanism could explain the reduced editing levels at the Q/R site of GRIA2 observed in human glioblastoma, where ADAR2 is normally expressed while ADAR3 expression is increased^63^. Analogously, we can suggest that the inhibition of ADAR2 activity by ADAR3 may be responsible for decreased RNA editing levels at the Q/R site of GRIA2 as well at other recoding sites in the LOAD brain.

In conclusion, post-mortem samples do not permit the study of transcriptomic changes during early stages of LOAD and can only evaluate the transcriptome alterations that characterize the final stages of neurodegeneration. Furthermore, the use of bulk RNA does not allow the identification of cell specific transcriptional deregulation, but currently, single-cell transcriptome analysis is not feasible on frozen post-mortem tissues. Despite these limitations, we believe that our study, providing a list of transcriptomics alterations in LOAD hippocampus, may represent an important resource for further molecular investigations aimed at better understanding the molecular mechanisms involved in LOAD and for the potential identification of novel therapeutic targets in LOAD.

## Methods

### Brain Samples

Frozen post-mortem brain tissue samples from cognitively normal controls, LOAD patients and PD patients were obtained from several non-profit brain banks: the NICHD Brain and Tissue Bank for Developmental Disorders (University of Maryland School of Medicine, Baltimore, USA), the London Neurodegenerative Diseases Brain Bank (King’s College Hospital, London, UK) and the Parkinson’s UK Brain Bank (Imperial College London, UK) (Table 1 and Supplementary Table S4). All patients provided their informed consent to the brain bank. All methods were carried out in accordance with relevant guidelines and regulations and the study was approved by the Institutional Review Board of the Institute of Biomembranes, Bioenergetics and Molecular Biotechnologies, National Research Council. Three brain regions, hippocampus (CA1 region), middle temporal gyrus (Brodmann’s Area 21) and middle frontal gyrus (Brodmann’s Area 46) were used in this study. All tissues contained both grey and white matter. Control subjects did not have a history of neurological disease or indications of brain abnormalities at tissue level, as determined at autopsy. LOAD patients were positive for Aβ plaques and NFTs and were selected according to dementia status (Braak V or VI). PD patients were positive for Lewy bodies in the substantia nigra.

### RNA Extraction

Total RNA and small RNA fractions were selectively extracted using the mirVana™ miRNA Isolation Kit (Thermo Fisher Scientific, Waltham, MA, USA) according to the manufacturer’s instructions. RNA was quantitatively and qualitatively evaluated using NanoDrop 2000c (Thermo Fisher Scientific) and Agilent Bioanalyzer 2100 (Agilent, Santa Clara, CA, USA), respectively. For total RNA, a RNA Integrity Number (RIN) ranging from 5.2 to 7.9 was obtained, which was considered acceptable as deriving from post-mortem tissues.

### RNA-seq

RNA-seq libraries were prepared from 1 µg of total hippocampal RNA, using the Illumina’s TruSeq Stranded Total RNA Sample Preparation Kit (Illumina, San Diego, CA, USA), according to the manufacturer’s protocol. cDNA libraries were checked on the Bioanalyzer 2100 and quantified by fluorimetry using the Quant-iTTM PicoGreen® dsDNA Assay Kit (Thermo Fisher Scientific) on NanoDrop™ 3300 Fluorospectrometer (Thermo Fisher Scientific). Sequencing was performed on NextSeq 500 platform, generating for each sample almost 100 millions of 100 bp paired-end reads. RNA-seq statistics are reported in Supplementary Table S1.

### Preprocessing and analysis of RNA-seq data

Reads with a Phred quality score (Q) > 20, a length higher of 50 nt and homopolymeric tract lower of the 50% of the total read length, were selected. In detail, reads in FASTQ format were inspected using FASTQC program (http://www.bioinformatics.babraham.ac.uk/projects/fastqc/) whereas adaptors and low quality regions were trimmed using Trim Galore (http://www.bioinformatics.babraham.ac.uk/projects/trim_galore/). Cleaned reads were aligned onto the complete latest human genome (assembly hg19) by means of GSNAP^64^ version 2013-11-27 (using as parameters: -B 5 -d hg19 -t5 -s splicesites -E1000 -N1 -n1 -Q -O–nofails -A sam–force-xs-dir -a paired) providing a list of exon-exon junctions from Ensembl, UCSC and RefSeq databases. Unique and concordant alignments in SAM format were converted in the binary BAM format by SAMtools^65^ and basic statistics were calculated using Picard tools (CollectRnaSeq Metrics.jar) (http://picard.sourceforge.net/).

Transcriptome quantification and differential expression of coding and non-coding RNA was performed using CuffDiff2 (http://cufflinks.cbcb.umd.edu/)^16^ software version 2.1.1 (using as main parameters:–library-type fr-secondstrand–labels Ctl,Als -u -b hg19.fa -M rRNA.gtf refgenes.gtf). The Principal Component Analysis (PCA) was conducted by ClustVis (http://biit.cs.ut.ee/clustvis/)^66^ on gene expression values obtained by CuffDiff2 on RNA-seq aligned reads. After the exclusion of outlier samples (Ctrl4 and AD2), CuffDiff2 analysis was repeated on the remaining samples. Reference human transcriptome was obtained from iGenomes repository (http://cole-trapnell-lab.github.io/cufflinks/igenometable/) and annotations for rRNA genes were downloaded from UCSC genome browser selecting the RepeatMask table. Differentially expressed genes were selected if |log2(FC)| > 1 and corrected P value < 0.05.

Differentially expressed coding RNAs were used as input to perform pathway enrichment analysis by IPA system (Ingenuity^®^ Systems, www.ingenuity.com), STRING tool (http://string-db.org)^17^ and Genatlas (http://genatlas.medecine.univ-paris5.fr/)^67^, to select transcripts for RT-qPCR validation.

### miRNA-seq

miRNA-seq libraries were prepared from 100 ng of small RNA fraction isolated from middle temporal gyrus, middle frontal gyrus and hippocampal samples, using the Illumina’s TruSeq Small RNA Sample Preparation Protocol, according to the manufacturer’s protocol. Libraries were checked on the Bioanalyzer 2100, quantified by fluorimetry using the Quant-iTTM PicoGreen® dsDNA Assay Kit on NanoDrop™ 3300 Fluorospectrometer and pooled in equomolar ratios to be sequenced on the Illumina MiSeq platform, generating for each sample approximately from 3 to 7 millions of 50 bp long reads. miRNA-seq statistics are reported in Supplementary Table S6.

### Preprocessing and analysis of miRNA-seq data

Adapter sequences were trimmed from raw reads using a custom Python script. Trimmed reads were mapped to human mature miRNA sequences, downloaded from miRBase (v21), using another *ad hoc* Python script, which required a best alignment with overlap of at least 17 bp, without mismatches, with an annotated mature miRNA sequence to assign miRNA identity. Only miRNAs with a read count higher than 10 were considered in the differential expression analysis. To assess the coherence of miRNA expression profiles, miRNA hierarchical cluster analysis was carried out using the R package pvclust (https://CRAN.R-project.org/package=pvclust)^68^ and computing pairwise-distances among samples. Differentially expressed miRNAs were identified using both DESeq2^69^ and in-house script methodologies, which generated substantially congruent conclusions. The in-house script method, although based on the log_2_ of fold change of expression levels, differs from DESeq2 method since: it does not use mathematical models to estimate the variance associated with the level of expression, it uses the upper quartile normalization system, it compares the log_2_ of fold change of expression levels intra- and inter-conditions, and it applies the T test (instead of the Wald test).

Potential miRNA-targeted genes and their impact on the biological pathways were assessed using DIANA-mirPath v2.0 (http://diana.imis.athena-innovation.gr)^70^.

### Reverse Transcription Quantitative PCR Analysis

#### Coding and non-coding RNA Expression Analysis

1 µg and 4 µg of total hippocampal RNA were used in the reverse transcription reaction (RT) for coding and non-coding RNA expression levels analysis, respectively, using the iScript™ Advanced cDNA Synthesis Kit (Bio-Rad Laboratories Ltd, Berkeley, California, USA), according to the manufacturer’s instructions. Quantitative PCR (qPCR) reactions were performed using 1 µl of the diluted cDNA (1:4) as input, specific TaqMan Gene Expression Assays and following the TaqMan Fast Advanced Master Mix Protocol (Thermo Fisher Scientific). IDs of the TaqMan Gene Expression Assays used are listed in Supplementary Table S5. RT-qPCR experiments were performed in triplicate for each sample on StepOnePlus Real-Time PCR System (Thermo Fisher Scientific). The expression of five housekeeping genes, CYC1, EIF4A2, GAPDH, HPRT1 and β-actin was also analyzed using two software applet for Microsoft Excel, named GeNorm^71^ and NormFinder^72^ in order to define the most stable housekeeping genes among samples, that resulted in selecting CYC1, EIF4A2 GAPDH genes. To normalize the data, the arithmetic Ct mean of housekeeping genes was evaluated for each sample; then, the transcript expression levels were calculated according to the ΔΔCt method, setting the arithmetic ΔCt mean of control group, as calibrator. The resulting data were represented as log2(ΔΔCt) and were expressed as the mean ± SD. Two-tailed Student’s T tests were performed to assess the statistical significance of gene expression levels differences observed between normal and LOAD samples. A p-value < 0.05 was considered statistically significant.

#### miRNA Expression Analysis

10 ng of small RNA fraction isolated from middle temporal gyrus, middle frontal gyrus and hippocampal samples were used to perform RT reactions using the TaqMan™ Advanced miRNA cDNA Synthesis Kit (Thermo Fisher Scientific), according to the manufacturer’s instructions. The resulting cDNA, diluted 1:10, was used as input for qPCR using specific TaqMan Advanced miRNA Assays and the TaqMan Fast Advanced Master Mix Protocol (Thermo Fisher Scientific). IDs of the TaqMan Advanced miRNA Assays used are listed in Supplementary Table S9. GeNorm and NormFinder tools were used to define the most stable miRNAs to be used as endogenous controls, that resulted to be: miR-423-3p (478327_mir), miR-181b-5p (478583_mir) and miR-191-5p (477952_mir). To normalize the data, the arithmetic Ct mean of the three stable miRNAs was evaluated for each sample; then, the miRNA expression levels were calculated according to the ΔΔCt method, setting the arithmetic ΔCt mean of control group as calibrator. The resulting data were represented as log2(ΔΔCt) and were expressed as the mean ± SD. Two-tailed Student’s T tests were performed to assess the statistical significance of miRNA expression levels differences observed between normal and LOAD samples. A p-value < 0.05 was considered statistically significant.

### Luciferase Reporter Assay

*In silico* evaluation of miRNA target genes was performed by using IPA’s microRNA Target Filter (www.ingenuity.com/products/IPA/micro RNA.html)^19^, that exploits TargetScan Human as source (http://www.targetscan.org/vert_50/)^73^, DIANA miRPath algorithm (http://diana.imis.athena-innovation.gr/DianaTools/index.php?r=mirpath/index)^31^ and Miranda (http://www.microrna.org/microrna/getGeneForm.do)^32^. To assessed miR184/*NR4A2* and miR184/*NRN1* interaction, the *NR4A2* and *NRN1* 3’UTRs, containing the miR-184 putative binding site, were amplified by PCR using the DreamTaq DNA Polymerase (Thermo Fisher Scientific) and cloned in the TA cloning vector pGEM-T Easy (Promega, Madison, WI, USA). Primers (5′ to 3′) used for cloning *NR4A2* were: Forward ACTAGTGCACAAGTATTACACATCAG and Reverse AAGCTTACGGTACATACAACACTTAC (SpeI and HindIII restriction sites are underlined). Primers (5′ to 3′) used for cloning *NRN1* were: Forward ACTAGTGCTTCCAGAAGACATGCTGC and Reverse AAGCTTGGTATTACTGTGTGTGTAACAGC. The recombinant vectors were then digested with SpeI and HindIII restriction enzymes and the isolated inserts were cloned in the SpeI and HindIII sites of the pMIR-REPORT luciferase reporter vector (Thermo Fisher Scientific), downstream the *luc2* firefly luciferase reporter gene, that is in turn under the control of a cytomegalovirus (CMV) promoter/termination system. Plasmid construct sequence was verified by Sanger sequencing. About 24 h before transfection, H1299 cells were plated at a density of 4 × 10^5^ cells/well in 6-well plates. Transfections of 1 µg of the recombinant Luciferase vector and 100 ng of the Renilla Luciferase Control Reporter vector pRL-SV40 (Thermo Fisher Scientific), used to normalize the transfection efficiency, as well as co-transfections of 30 pmol of control miRNA (Negative Control #1, Thermo Fisher Scientific) or mimic hsa-miR-184 (Thermo Fisher Scientific) and/or anti-miR-184 (Thermo Fisher Scientific), were performed using Lipofectamine 2000 (Thermo Fisher Scientific) in 80-90% confluent H1299 cells. About 48 h after transfection, firefly and Renilla luciferase activity were evaluated by using the Dual-luciferase Reporter Assay Kit (Promega), following the manufacturer’s instructions, and the Turner Designs Luminometer Model TD-20/20 (Promega). Firefly luciferase activity was normalized to Renilla expression for each sample and calibrated respected to the normalized value of the control sample (H1299 cells transfected with control miRNA). Data represent the means ± SDs from the results of three independent experiments. Statistical analysis was performed by Two-tailed Student’s T tests and a p-value < 0.05 was considered statistically significant.

### RNA editing analysis

RNA editing candidates were detected using REDItools^74^. The Alu editing index, the weighted average editing level across all expressed Alu sequences, was calculated using custom scripts and according to the methodology described in Bazak *et al*.^75^. RNA editing levels in recoding sites were assessed using REDItools and providing a list of 1585 known positions from REDIportal database^37^ in which RNA editing causes amino acid change. Only positions supported by RNAseq reads in at least three samples per group and showing a median editing level higher than 0.1 were used to calculate dysregulated RNA editing. A t-test was used to assess editing dysregulation for each RNA site. P-values were corrected using Benjamini-Hochberg with FDR = 0.1. ADAR expressions in LOAD and control samples were calculated using CuffDiff2^16^.

### Statistics

Differentially expressed genes in RNA-seq analysis were defined by using CuffDiff2. miRNA-seq data were statistically analyzed by applying DESeq2 both for data normalization and comparison (Wald test) and in-house procedure relying in the upper quartile normalization and T test for comparisons. For RT-qPCR and luciferase analyses, the statistical significance was assessed by Two-tailed Student’s T tests and results were expressed as the means ± SD. For RNA editing analyses, the statistical significance was assessed by using T test followed by Benjamini-Hochberg procedure for multiple test p-values correction. *P*-values less than 0.05 were considered to indicate statistical significance.

## Electronic supplementary material

Supplementary Material
