# Supplementary material for: Whole transcriptome profiling of Late-Onset Alzheimer’s Disease patients provides insights into the molecular changes involved in the disease
Source: Sci Rep. 2018 Mar 9;8:4282. doi: 10.1038/s41598-018-22701-2 (PMC5844946; doi:10.1038/s41598-018-22701-2)
Supplement: Supplementary file 1 — Supplementary Material [file 41598_2018_22701_MOESM1_ESM.pdf]

Supplementary Material

Whole transcriptome profiling of Late-Onset Alzheimer's Disease patients provides insights into the molecular changes involved in the disease

Anita Annese[1], Caterina Manzari[1], Claudia Lionetti[1], Ernesto Picardi[1,2], David S. Horner[1,3], Matteo Chiara[3], Mariano Francesco Caratozzolo[1], Apollonia Tullo[1], Bruno Fosso[1], Graziano Pesole[1,2,4,*], Anna Maria D'Erchia[1,2,*]

[1] Institute of Biomembranes, Bioenergetics and Molecular Biotechnologies, National Research Council, Via Amendola 165/A, 70126 Bari, Italy

[2] Department of Biosciences, Biotechnology and Biopharmaceutics, University of Bari, Via Orabona 4, 70126 Bari, Italy

[3] Department of Biosciences, University of Milan, Via Celoria 26, 20133 Milan, Italy

[4] Center of Excellence in Comparative Genomics, University of Bari, Piazza Umberto I, 70121 Bari, Italy

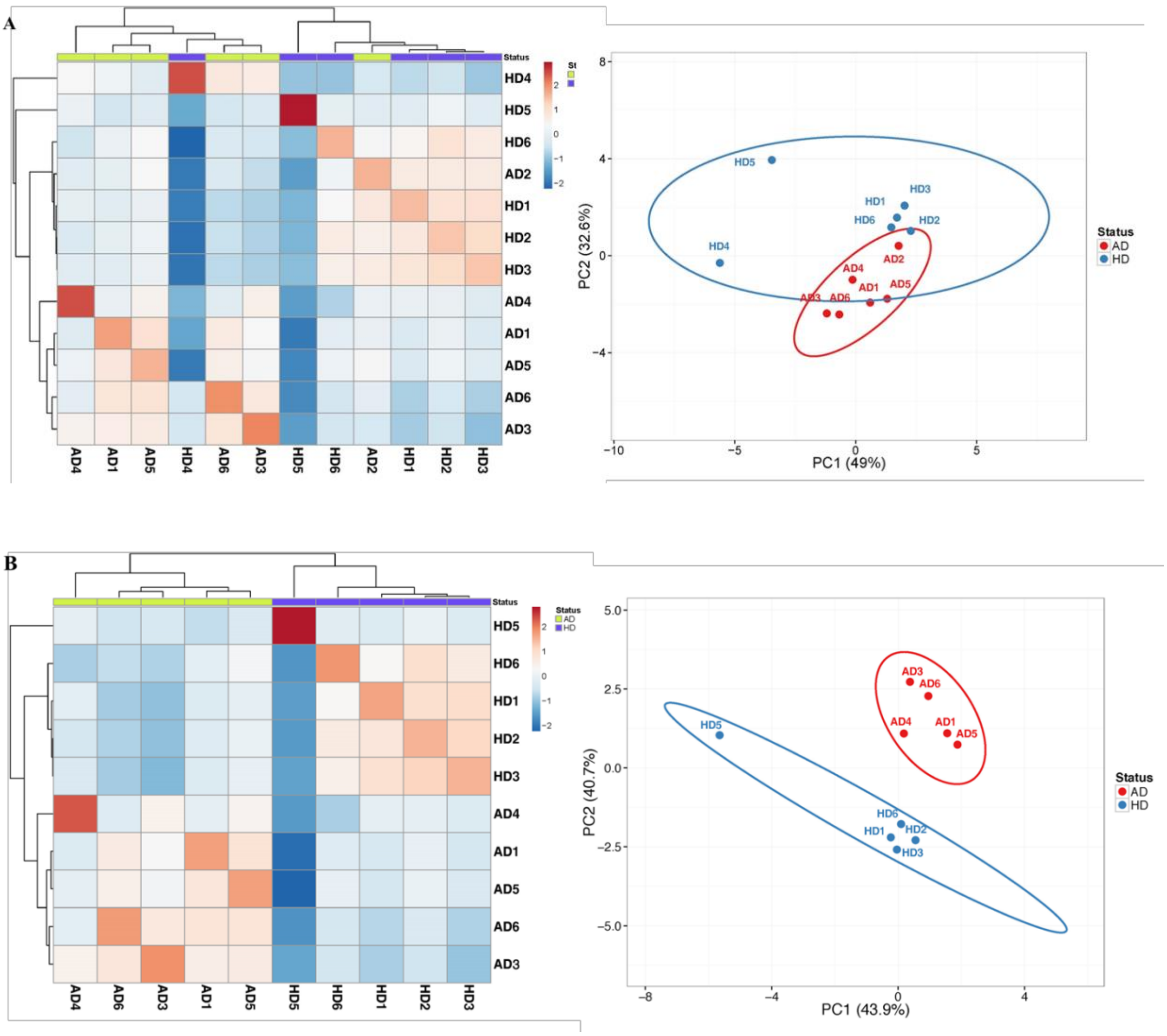

Supplementary Figure S1. Heatmap and PCA of RNA-Seq data.
HD1-6: control samples (Ctrl1-6); AD 1-6: LOAD samples (LOAD 1-6).
(A) HD4 (Ctrl4) and AD2 (LOAD2) samples resulted as outliers. (**B**) The exclusion of Ctrl4 and LOAD2 samples produced a better clusterization of LOAD patients and controls.

A

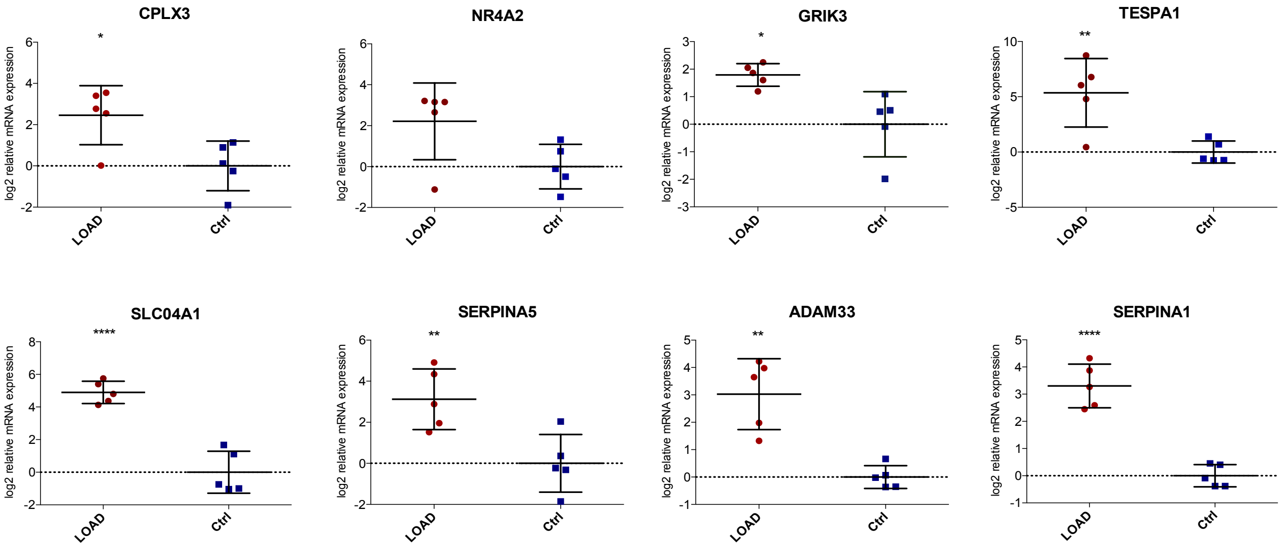

B

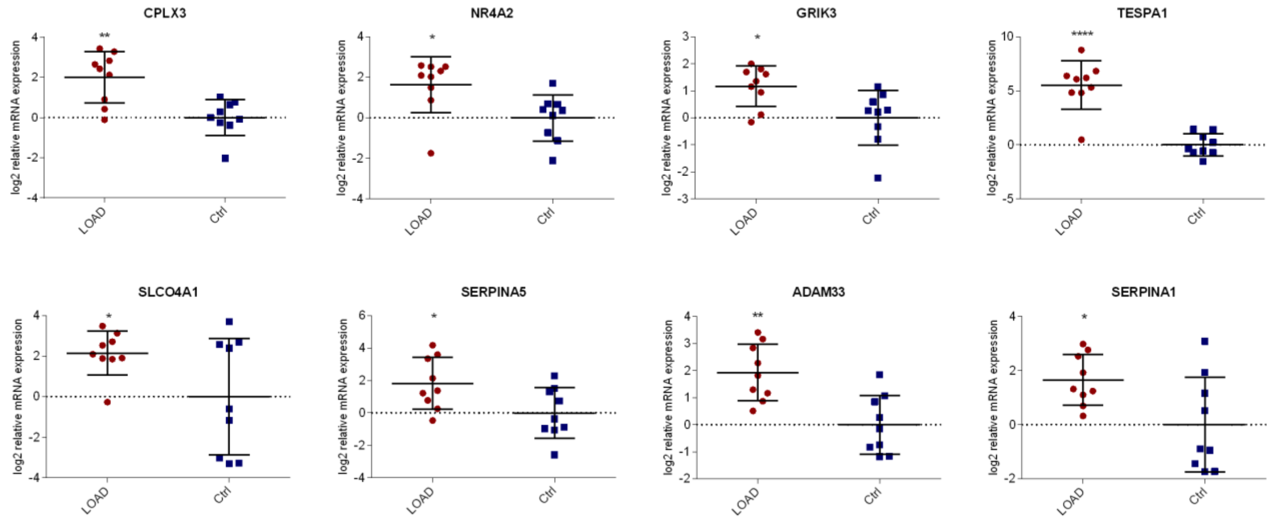

Supplementary Figure S2. RT-qPCR validation of upregulated protein coding genes of the RNA-seq dataset in LOAD hippocampus.

(A): original cohorts (5 controls and 5 LOAD patients); **(B)**: enlarged cohorts (9 controls and 9 LOAD patients). LOAD: Group of LOAD patients; Ctrl: Group of control subjects

The data are expressed as the means of log2(ΔΔCt) ± SD. P-value was calculated by T-test. *: p-value ≤ 0.05; **: p value ≤ 0.01; ****: p value ≤ 0.0001.

A

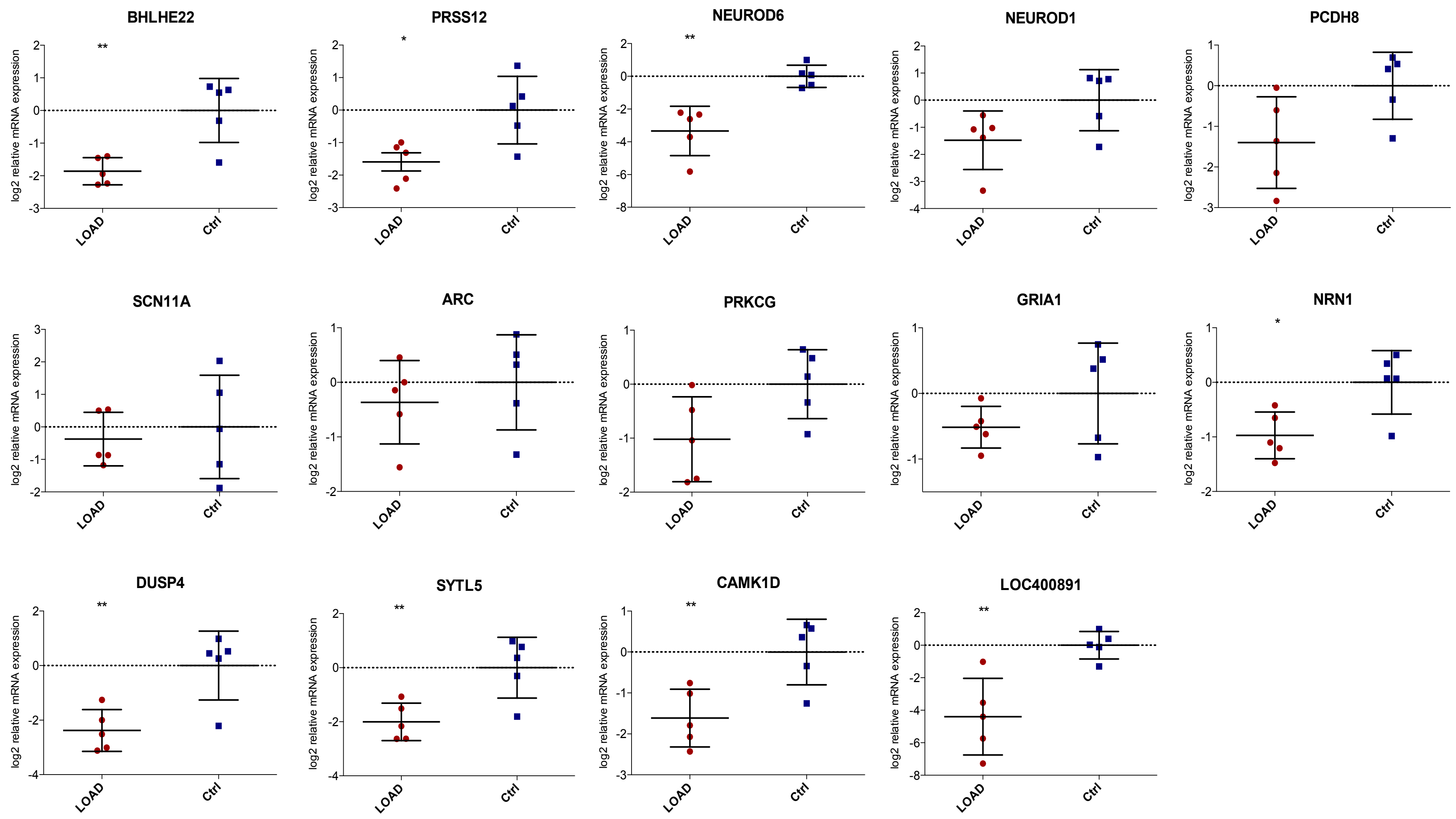
BHLHE22
**
PRSS12
*
NEUROD6
**
NEUROD1
PCDH8
SCN11A
ARC
PRKCG
GRIA1
NRN1
*
DUSP4
**
SYTL5
**
CAMK1D
**
LOC400891
**
log2 relative mRNA expression
LOAD
Ctrl

B

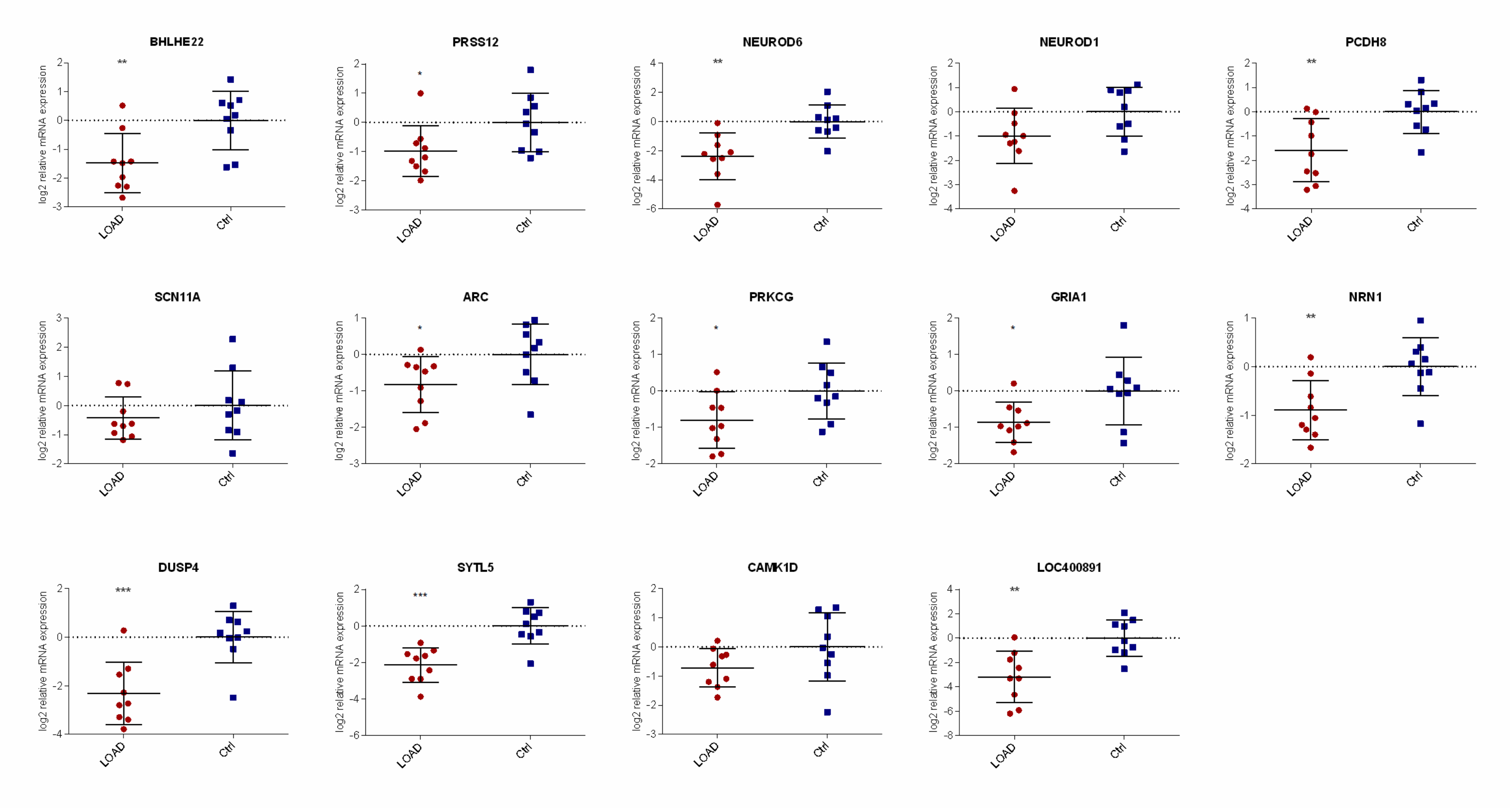

Supplementary Figure S3. RT-qPCR validation of 13 LOAD downregulated protein coding genes and 1 lncRNA of the RNA-seq dataset in LOAD hippocampus.

(A): original cohorts (5 controls and 5 LOAD patients); **(B)**: enlarged cohorts (9 controls and 9 LOAD patients).

LOAD: Group of LOAD patients; Ctrl: Group of control subjects.

The data are expressed as the means of log2(ΔΔCt) ± SD. P-value was calculated by T-test. *: p-value ≤ 0.05; **: p value ≤ 0.01; ***: p-value ≤ 0.001.

A

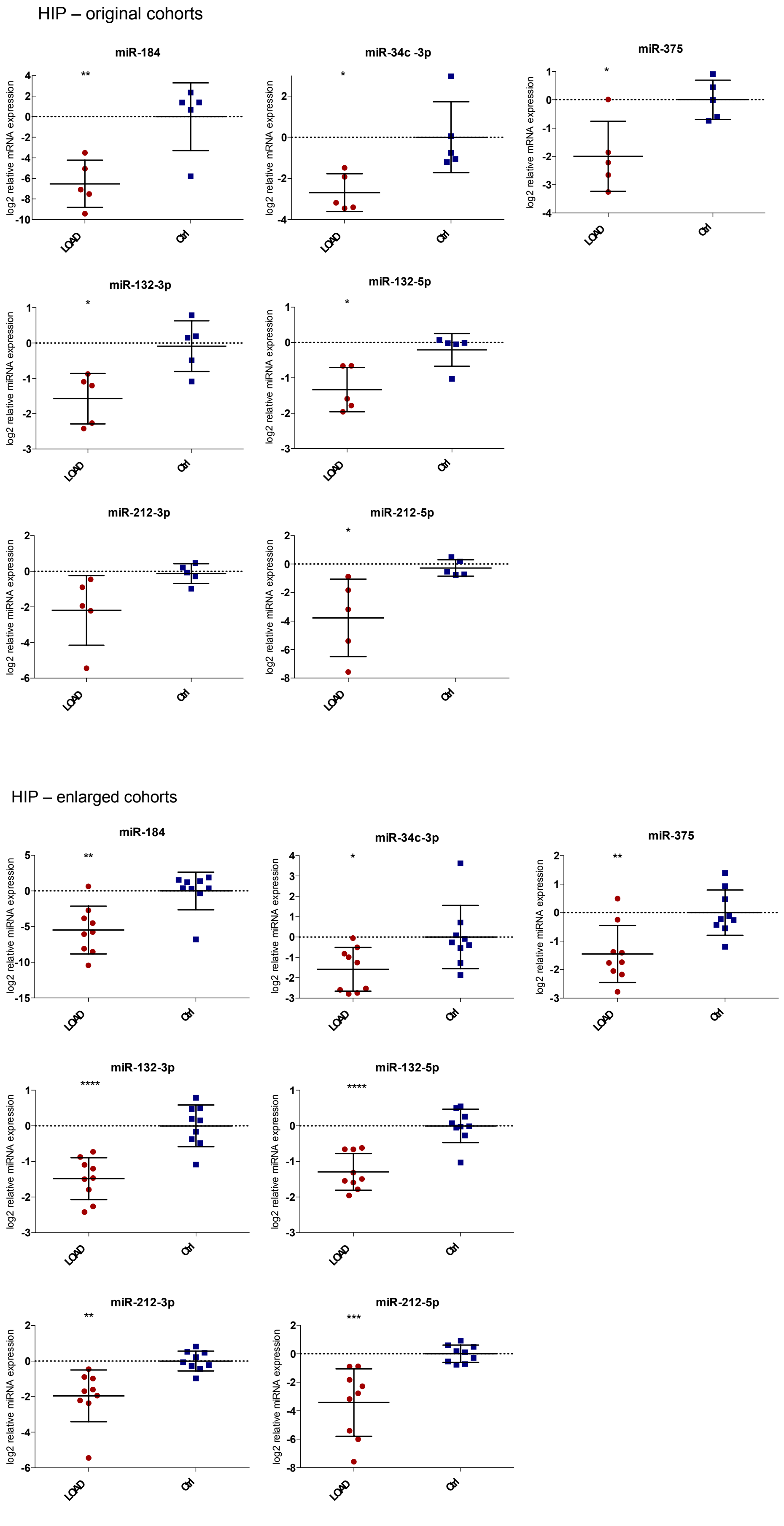

HIP – original cohorts
miR-184
**
log2 relative mRNA expression
LOAD
Ctrl
miR-34c -3p
*
log2 relative mRNA expression
LOAD
Ctrl
miR-375
*
log2 relative mRNA expression
LOAD
Ctrl
miR-132-3p
*
log2 relative miRNA expression
LOAD
Ctrl
miR-132-5p
*
log2 relative miRNA expression
LOAD
Ctrl
miR-212-3p
log2 relative miRNA expression
LOAD
Ctrl
miR-212-5p
*
log2 relative miRNA expression
LOAD
Ctrl
HIP – enlarged cohorts
miR-184
**
log2 relative miRNA expression
LOAD
Ctrl
miR-34c-3p
*
log2 relative miRNA expression
LOAD
Ctrl
miR-375
**
log2 relative miRNA expression
LOAD
Ctrl
miR-132-3p
****
log2 relative miRNA expression
LOAD
Ctrl
miR-132-5p
****
log2 relative miRNA expression
LOAD
Ctrl
miR-212-3p
**
log2 relative miRNA expression
LOAD
Ctrl
miR-212-5p
***
log2 relative miRNA expression
LOAD
Ctrl

B

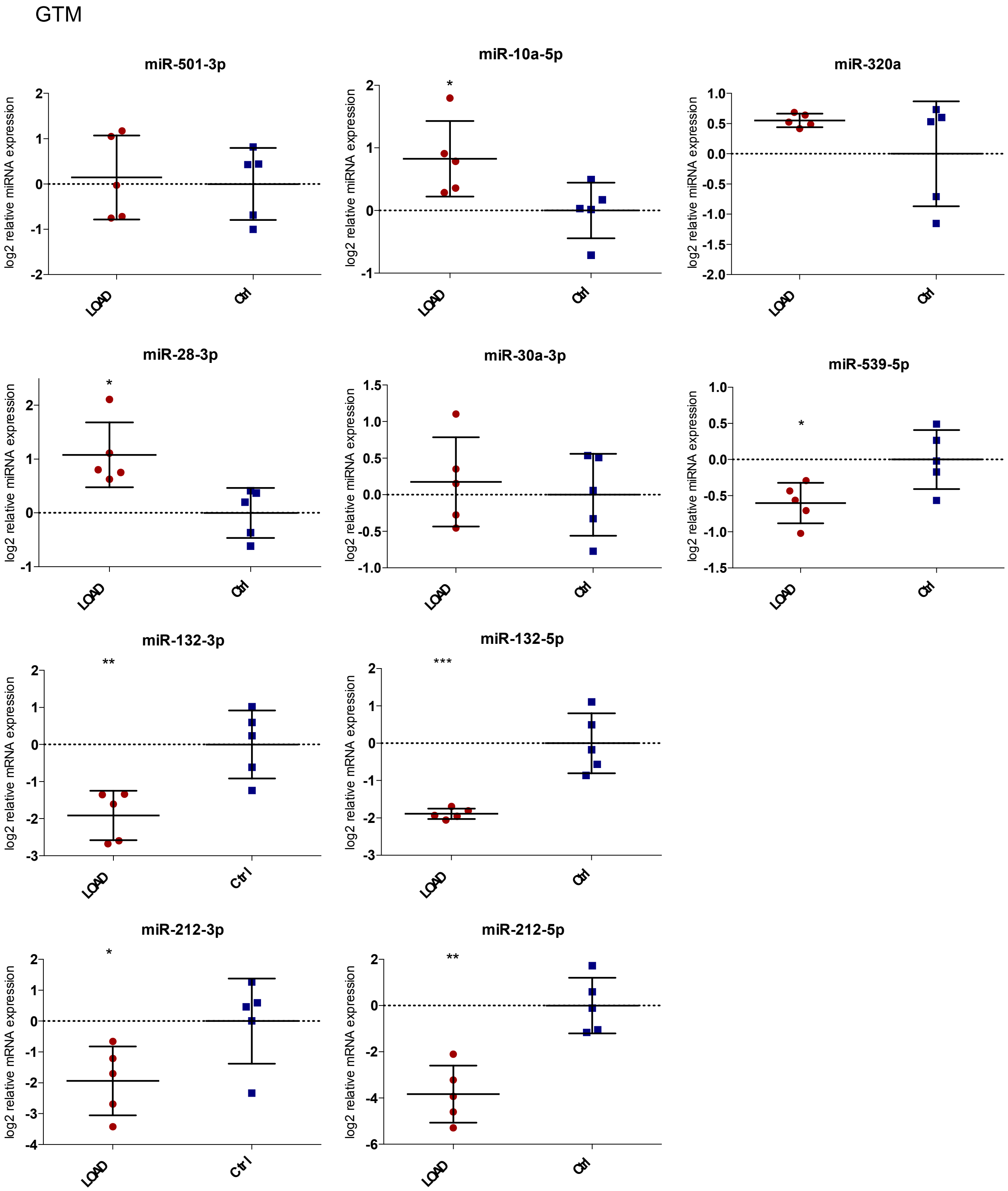
GTM
miR-501-3p
miR-10a-5p
miR-320a
miR-28-3p
miR-30a-3p
miR-539-5p
miR-132-3p
miR-132-5p
miR-212-3p
miR-212-5p
log2 relative miRNA expression
log2 relative mRNA expression
LOAD
Ctrl
*
**
***

C

GFM

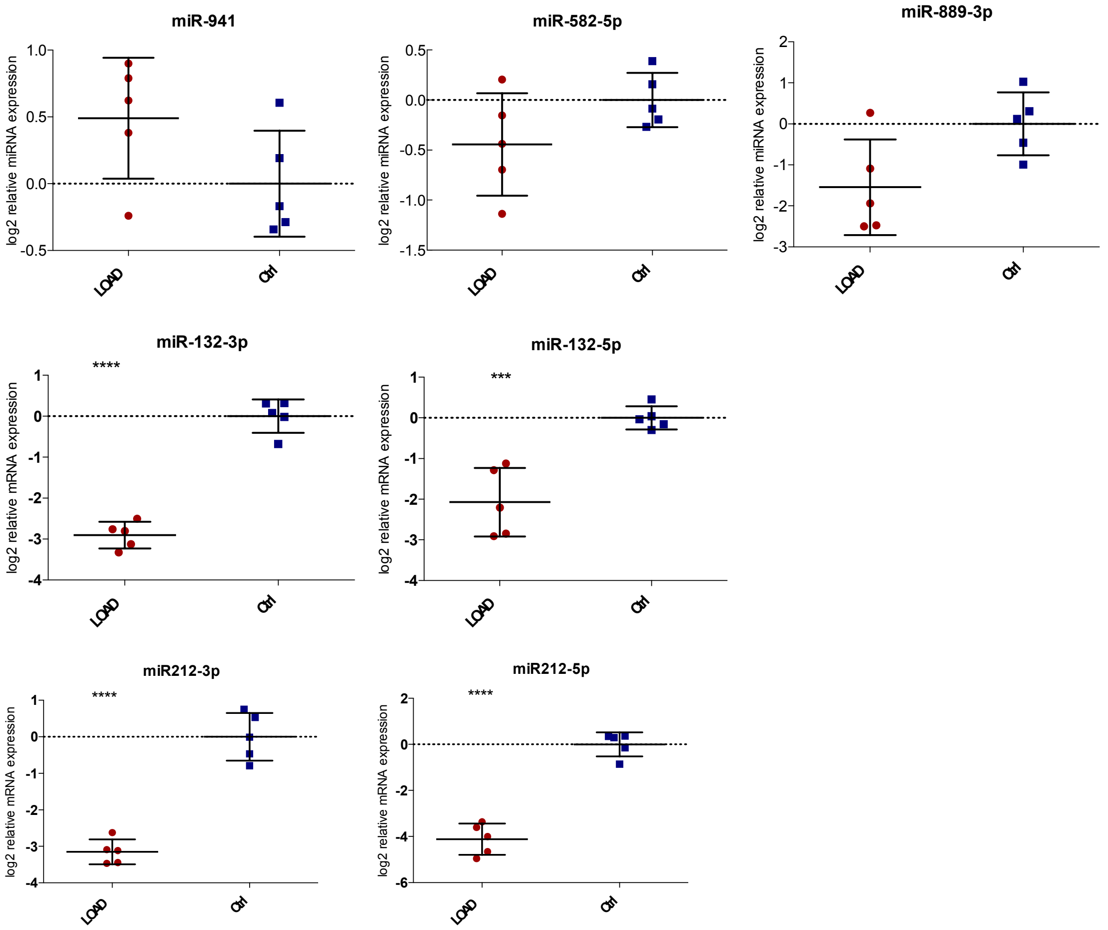

Supplementary Figure S4. RT-qPCR assay confirmed the miRNA expression trend shown in the miRNA-Seq analysis in the LOAD hippocampus (HIPP) (A), middle temporal gyrus (GTM) (B) and middle frontal gyrus (GFM) (C). LOAD: Group of LOAD patients; Ctrl: Group of control subjects.

(A) In the LOAD hippocampus miRNAs expression levels were validated by RT-qPCR in the original cohorts (5 controls and 5 LOAD patients) and in the enlarged cohorts (9 controls and 9 LOAD patients).

(B) In the middle temporal gyrus miRNAs expression levels were validated by RT-qPCR in the original cohorts (5 controls and 5 LOAD patients).

(C) In the middle frontal gyrus miRNAs expression levels were validated by RT-qPCR in the original cohorts (5 controls and 5 LOAD patients).

The data are expressed as the means of log2(ΔΔCt) ± SD. P-value was calculated by T-test. *: p-value ≤ 0.05; **: p value ≤ 0.01; ***: p-value ≤ 0.001; ****: p-value ≤ 0.0001.

A

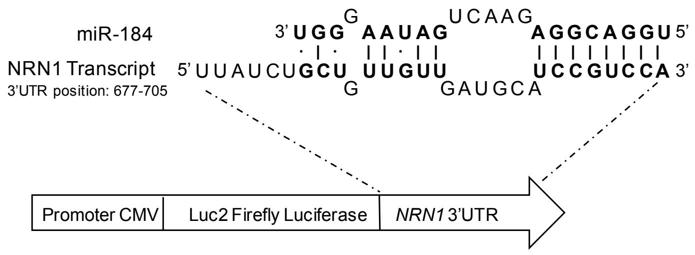

B

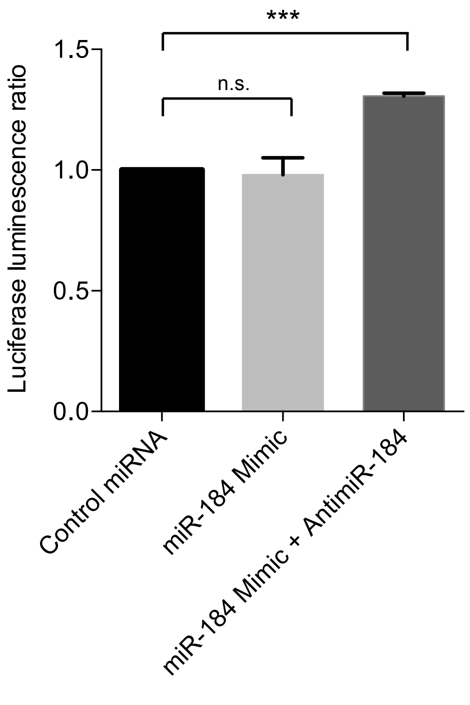

Supplementary Figure S5. Experimental evaluation of miR-184/*NRN1* interaction by luciferase assay.

(A) Schematic representation of the 3'UTR of *NRN1* transcript (NM_016588.2) cloned in the pMIR-reporter luciferase miRNA expression vector, downstream the firefly luciferase gene. The sequence alignment between miR-184 seed region and *NRN1* 3'UTR is reported.

(B) Luciferase assay. H1299 cells were transfected with a negative control miRNA mimic, mir-184 mimic alone and with anti-miR-184 along with pMIR luciferase reporter vectors containing *NRN1* 3'UTR. Luciferase expression was normalized by Renilla expression and by calibrating the results data against the control sample (H1299 cells transfected with the control miRNA mimic). Data represent the means ± SD from the results of three independent experiments. P-value was calculated by T-test. n.s.: not significant; ***: p-value < 0.001.

Case ID	Sample	Original Reads	Reads after filtering	Reads after rRNA removal	% Aligned
5028	Ctrl1	170,087,337	162,208,459	157,130,648	92.38
5174	Ctrl2	169,442,296	161,233,038	149,838,855	88.43
5247	Ctrl3	165,037,459	157,761,964	152,497,046	92.40
5352	Ctrl4	186,126,444	178,140,803	171,510,319	92.15
5533	Ctrl5	146,321,310	139,354,597	134,646,801	92.02
5362	Ctrl6	153,989,368	146,050,462	139,075,608	90.32
1625	AD1	159,856,680	152,220,646	145,487,327	91.01
4737	AD2	153,670,958	146,658,614	142,227,477	92.55
5195	AD3	173,256,469	164,979,346	147,975,278	85.41
5198	AD4	178,672,115	169,149,188	160,669,030	89.92
1946	AD5	188,847,245	179,102,843	162,773,140	86.19
3136	AD6	150,233,101	143,128,942	132,954,147	88.50
1272	PD1	98,064,291	93,007,945	88,291,680	90.03
1741	PD2	120,794,493	114,026,750	110,873,158	91.87
1901	PD3	96,965,168	91,922,726	89,000,005	91.79
4526	PD4	94,971,843	89,608,126	83,608,812	88.04
5329	PD5	91,525,386	87,107,791	84,201,270	92.00
5520	PD6	92,250,528	87,777,038	83,322,558	90.32

Supplementary Table S1. Statistics of hippocampal RNA-seq data. Number of sequencing reads before and after filtering and rRNA removal and percentage of reads aligned to the human genome hg19.

Legend
Gene Symbol: Gene Symbol from RefSeq annotations
Locus: Genomic locus according to hg19 human genome assembly
value_Ctrl: Mean expression level (in FPKM) per Control group calculated by CuffDiff2
value_LOAD: Mean expression level (in FPKM) per ALS group calculated by CuffDiff2
log2(fold_change): Log2 of the ratio value_Ctrl/value_LOAD calculated by CuffDiff2
test_stat: Statistical Test value calculated by CuffDiff2
p_value: P value calculated by CuffDiff2
q_value: Corrected P value calculated by CuffDiff2

Gene symbol	locus	value_Ctrl	value_LOAD	log2(fold_change)	test_stat	p_value	q_value
TESPA1	chr12:55342086-55378530	0.234793	5.09992	4.44102	4.15414	0.00005	0.00136643
HLA-DRB5	chr6:32485153-32498006	0.492694	7.67076	3.96061	2.65079	0.00005	0.00136643
ROS1	chr6:117609529-117747018	0.0622164	0.902891	3.85918	2.5483	0.00005	0.00136643
ANKRD22	chr10:90562486-90611732	0.0469512	0.680935	3.85828	2.21349	0.00015	0.00330506
RGS1	chr1:192544856-192549159	2.40179	34.5989	3.84854	3.9466	0.00005	0.00136643
HAMP	chr19:35773409-35776045	1.05482	12.9969	3.62309	2.86284	0.00005	0.00136643
HLA-DRB1	chr6_ssto_hap7:3850433-3993164	1.79041	20.935	3.54755	2.96427	0.00225	0.024287
KRT17	chr17:39775691-39780882	0.179545	2.03864	3.50519	2.15172	0.00005	0.00136643
EMR1	chr19:6887559-6940464	0.0480178	0.531098	3.46734	1.97245	0.00005	0.00136643
SELE	chr1:169691780-169703220	0.156133	1.6795	3.42718	2.12171	0.0003	0.00551994
FPR1	chr19:52249022-52255150	1.77783	18.2455	3.35935	3.13388	0.00005	0.00136643
GPR78	chr4:8581963-8591750	0.0510444	0.509667	3.31973	1.97165	0.0029	0.0292774
REREP3	chr15:22546564-22570831	0.0638269	0.614128	3.2663	1.61955	0.0032	0.0313892
HLA-DQA1	chr6:32605182-32611429	0.820133	7.83233	3.25551	2.43195	0.00005	0.00136643
RNASE2	chr14:21423629-21424594	0.28265	2.69234	3.25177	2.21728	0.00005	0.00136643
ALOX15B	chr17:7942357-7952451	0.28581	2.58142	3.17504	2.14815	0.00025	0.00483727
SLCO4A1	chr20:61273796-61303647	3.16586	28.2665	3.15842	3.9143	0.00005	0.00136643
HLA-DRA	chr6_ssto_hap7:3754282-3759493	0.202611	1.7851	3.13922	1.79864	0.00145	0.0177865
CXCL1	chr4:74735108-74737019	0.294232	2.54491	3.11259	1.89669	0.00045	0.00751151
HLA-A	chr6_ssto_hap7:1239581-1243012	0.875311	7.40648	3.08092	2.14913	0.00005	0.00136643
PCK1	chr20:56136136-56141513	0.34455	2.89461	3.07059	2.67355	0.00005	0.00136643
TNFSF14	chr19:6663147-6670599	0.0735007	0.612106	3.05795	1.76849	0.00065	0.00986869
VSIG4	chrX:65241579-65259967	3.73591	28.911	2.95209	3.52454	0.00005	0.00136643
FCGR1C	chr1:149369293-149378303	0.260669	1.9983	2.93848	2.07346	0.00005	0.00136643
SIGLEC7	chr19:51645557-51685222	0.134233	0.978251	2.86546	1.79536	0.00035	0.00620045
SIGLEC5	chr19:52114755-52133727	0.0533325	0.385179	2.85244	1.73494	0.0017	0.0198318
APOL4	chr22:36585175-36600879	0.42988	3.05942	2.83126	2.63103	0.00005	0.00136643
MT1H	chr16:56703725-56705041	1.19063	8.41223	2.82076	1.94333	0.00005	0.00136643
BCL2A1	chr15:80253231-80263643	0.339338	2.39064	2.8166	1.63298	0.0012	0.015451
C10orf10	chr10:45455218-45490172	9.62922	66.2173	2.78172	2.18657	0.00095	0.0131856
C7orf61	chr7:100054237-100061894	1.10648	7.48575	2.75817	2.2643	0.0001	0.00238525
CD163	chr12:7623411-7656414	4.16621	27.944	2.74573	2.9102	0.00005	0.00136643
SIGLEC9	chr19:51628136-51639520	0.714656	4.69284	2.71514	2.37602	0.00005	0.00136643
SLC11A1	chr2:219246751-219261617	1.18496	7.7561	2.71049	2.51684	0.00005	0.00136643
MEP1A	chr6:46761093-46807519	0.0471542	0.30643	2.7001	1.77123	0.00015	0.00330506
GABRR2	chr6:89966839-90025018	0.0689754	0.445533	2.69138	1.7916	0.0003	0.00551994
LINC01094	chr4:79567147-79605655	8.86678	56.5264	2.67244	2.97815	0.00005	0.00136643

SECTM1	chr17:80278899-80291921	0.193079	1.22579	2.66645	1.69636	0.003	0.0299609
HSPA6	chr1:161494035-161496687	0.490462	3.09642	2.65838	2.27765	0.0001	0.00238525
CASP5	chr11:104864966-104893895	0.0344441	0.216445	2.65167	0.923813	0.0033	0.0321732
SERPINA5	chr14:95047705-95059457	1.37174	8.6045	2.64909	2.18908	0.00005	0.00136643
MKI67	chr10:129894924-129924468	0.0339712	0.21283	2.64732	2.05252	0.00005	0.00136643
HIST1H3F	chr6:26250369-26250835	0.305687	1.88049	2.62098	1.57757	0.00565	0.0476976
C9orf153	chr9:88835179-88874572	0.145679	0.895024	2.61913	1.99629	0.00005	0.00136643
SCIN	chr7:12610202-12693228	2.47263	14.9123	2.59239	3.3487	0.00005	0.00136643
PLAC8	chr4:84011210-84035911	0.463386	2.74158	2.56472	1.87844	0.0002	0.00407561
MARCO	chr2:119699744-119752236	0.430448	2.54414	2.56327	2.01135	0.00005	0.00136643
HSD17B1	chr17:40703983-40707232	0.511214	2.99636	2.55121	2.11301	0.00005	0.00136643
GPR65	chr14:88471467-88481155	0.330091	1.91419	2.5358	2.36271	0.00005	0.00136643
C1QB	chr1:22979681-22988029	25.2019	145.838	2.53276	3.66332	0.00005	0.00136643
HILS1	chr17:48243365-48253293	0.346938	1.99245	2.52179	1.48813	0.0032	0.0313892
CYP26A1	chr10:94833231-94837641	0.118341	0.67786	2.51804	1.66984	0.0002	0.00407561
SERPINA1	chr14:94843083-94857029	1.03554	5.90977	2.51271	2.69441	0.00005	0.00136643
LRG1	chr19:4537226-4540036	0.188701	1.05552	2.48378	1.57423	0.0021	0.0232225
NGFR	chr17:47572654-47651426	0.894795	4.90999	2.45609	2.1733	0.00005	0.00136643
HIST1H1B	chr6:27834569-27835359	0.567939	3.11128	2.4537	1.68614	0.0004	0.00687883
IQGAP3	chr1:156495196-156542396	0.0509981	0.27932	2.4534	1.71354	0.00015	0.00330506
SLC52A3	chr20:740723-749228	0.918123	5.00377	2.44625	2.43773	0.00005	0.00136643
NOD2	chr16:50731049-50766987	0.131214	0.709879	2.43565	1.93227	0.0001	0.00238525
CD14	chr5:140011312-140013286	6.83575	36.9283	2.43356	3.21033	0.00005	0.00136643
FCGR3A	chr1:161511550-161520413	4.88749	26.2197	2.42349	3.05174	0.00005	0.00136643
GPR4	chr19:46093022-46105466	0.405034	2.16082	2.41546	1.96233	0.00005	0.00136643
C1QA	chr1:22963117-22966175	13.4275	71.6105	2.41499	3.26647	0.00005	0.00136643
C1QC	chr1:22970117-22974603	18.1276	95.1731	2.39236	3.5419	0.00005	0.00136643
ADAM33	chr20:3648619-3662778	0.890307	4.67229	2.39175	2.79166	0.00005	0.00136643
SOCS3	chr17:76352857-76356160	4.27673	22.3457	2.38542	2.64979	0.00005	0.00136643
NPC1L1	chr7:44552134-44580914	0.0531193	0.276908	2.3821	1.37402	0.00585	0.0490104
FCGR2A	chr1:161475204-161489360	2.87233	14.6749	2.35306	2.92577	0.00005	0.00136643
FAM167B	chr1:32712817-32714461	0.259348	1.31149	2.33824	1.56988	0.0003	0.00551994
CR1	chr1:207669472-207815110	0.183699	0.928267	2.33719	2.04697	0.0002	0.00407561
FCGBP	chr19:40353962-40440533	1.79771	8.96426	2.31803	3.4433	0.00005	0.00136643
CHI3L2	chr1:111770280-111786062	2.97908	14.7683	2.30956	2.42727	0.00005	0.00136643
CD180	chr5:66478103-66492617	0.333566	1.65075	2.30708	1.88855	0.00025	0.00483727
MAFB	chr20:39314487-39317880	2.16052	10.5301	2.28507	2.88017	0.00005	0.00136643
FCGR1B	chr1:120926127-120935944	0.456707	2.21819	2.28004	1.94445	0.00105	0.0140962
SIGLEC8	chr19:51954250-51961708	1.75787	8.42797	2.26136	2.61159	0.00005	0.00136643
HERC2P3	chr15:20613649-20711433	4.42994	21.1451	2.25497	3.0017	0.00005	0.00136643
LILRA4	chr19:54844691-54850421	0.660935	3.14404	2.25004	1.91503	0.00055	0.00876569
LOC10012931	chr9:93825575-93837414	0.116074	0.551119	2.24731	1.74637	0.00045	0.00751151
FOXF1	chr16:86544132-86548070	0.63718	2.99056	2.23064	1.85329	0.00045	0.00751151
TLR2	chr4:154605440-154627242	1.98371	9.28408	2.22656	2.74038	0.00005	0.00136643
DPP4	chr2:162848754-162931052	0.0895366	0.418594	2.225	1.64232	0.00035	0.00620045
BCL6B	chr17:6926368-6932961	0.497171	2.29981	2.2097	1.84056	0.00075	0.0109258
PCAT19	chr19:41960073-42006554	0.32616	1.50597	2.20704	1.89384	0.00025	0.00483727
KCNE4	chr2:223916647-223920357	2.09298	9.62336	2.20098	2.75371	0.00005	0.00136643

ICAM1	chr19:10381516-10397291	2.04519	9.31114	2.18673	2.74336	0.00005	0.00136643
CCR5	chr3:46411632-46417697	0.306442	1.39399	2.18553	1.73485	0.00075	0.0109258
OTOS	chr2:241078445-241080073	2.34795	10.6513	2.18156	1.73101	0.00045	0.00751151
MS4A6A	chr11:59939079-59952139	2.69336	12.1791	2.17693	2.48311	0.00005	0.00136643
SLC16A3	chr17:80186281-80197375	1.9766	8.89162	2.16942	2.3497	0.00005	0.00136643
SAP25	chr7:100169852-100171270	0.801919	3.59087	2.16281	1.68641	0.0007	0.010395
MYO1G	chr7:45002259-45018704	0.221718	0.992615	2.16251	1.72288	0.0002	0.00407561
HIF3A	chr19:46720994-46846690	4.02655	17.7433	2.13966	2.74503	0.00005	0.00136643
TLR5	chr1:223282747-223316624	0.868623	3.79722	2.12814	2.39996	0.00005	0.00136643
H19	chr11:2016405-2019065	0.174503	0.761661	2.12589	1.51026	0.0011	0.0145132
ATAD3C	chr1:1385068-1405538	0.335656	1.46447	2.12532	1.91143	0.00005	0.00136643
ANGPTL4	chr19:8429010-8439259	7.59757	33.0822	2.12245	2.99945	0.00005	0.00136643
HSPB1	chr7:75931874-75933614	33.2575	144.357	2.11789	3.1785	0.00005	0.00136643
LOC10050613	chr7:96250968-96293650	0.440879	1.90368	2.11034	1.68193	0.00095	0.0131856
FGR	chr1:27938800-27961727	1.02262	4.40737	2.10765	2.17584	0.00005	0.00136643
LAIR1	chr19:54865232-54882163	2.18172	9.37877	2.10393	2.54448	0.00005	0.00136643
KLHL6	chr3:183205318-183273499	0.487995	2.0885	2.09753	2.32358	0.00005	0.00136643
SIGLEC10	chr19:51913274-51921057	1.27518	5.41624	2.08659	2.42768	0.00005	0.00136643
SPP1	chr4:88896801-88904563	75.1744	319.122	2.0858	3.06633	0.00005	0.00136643
PIM1	chr6:37137921-37143204	3.46125	14.677	2.08419	2.68907	0.00005	0.00136643
MSR1	chr8:15965386-16050300	1.81831	7.67707	2.07796	2.23315	0.00005	0.00136643
CLEC5A	chr7:141627156-141646783	0.444049	1.87081	2.07488	1.88682	0.00005	0.00136643
CHRNB3	chr8:42552561-42592209	0.095761	0.400986	2.06604	1.39483	0.0018	0.0207648
FGF17	chr8:21900427-21906319	1.26999	5.23873	2.0444	1.67216	0.0007	0.010395
C3	chr19:6677845-6720662	29.141	120.12	2.04336	2.60618	0.00005	0.00136643
ZNF812	chr19:9800813-9811493	0.191663	0.789617	2.04258	1.52261	0.0005	0.00814556
EBI3	chr19:4229539-4237524	0.838918	3.45237	2.04099	1.66625	0.00075	0.0109258
FCGR2C	chr1:161551128-161571010	0.535976	2.20486	2.04045	1.86702	0.00005	0.00136643
TLR8	chrX:12920935-12961419	0.190207	0.781483	2.03864	1.58088	0.0013	0.0163446
NAPSA	chr19:50861733-50868931	0.146811	0.600178	2.03143	1.36302	0.0027	0.0277348
GADD45B	chr19:2476122-2478257	9.33011	37.8659	2.02093	2.5722	0.00005	0.00136643
PARVG	chr22:44568835-44604349	1.07221	4.31664	2.00932	2.23872	0.00005	0.00136643
CD300A	chr17:72462521-72480937	1.33131	5.33464	2.00254	2.04097	0.00015	0.00330506
PLAUR	chr19:44150246-44174498	0.986545	3.95073	2.00166	1.76254	0.00055	0.00876569
CD177	chr19:43857810-43867324	0.15989	0.638684	1.99803	1.39642	0.0017	0.0198318
TFEC	chr7:115575201-115670867	0.74012	2.94356	1.99173	2.2487	0.00005	0.00136643
LRP2	chr2:169983618-170219122	4.03021	16.0184	1.99081	2.79116	0.00005	0.00136643
SLC1A5	chr19:47278139-47291842	1.99055	7.85886	1.98115	2.02553	0.0001	0.00238525
ABI3	chr17:47283595-47300587	1.27415	5.013	1.97614	2.01134	0.00005	0.00136643
HILPDA	chr7:128095883-128098472	9.91978	38.8498	1.96953	2.48682	0.00005	0.00136643
RUNX1	chr21:36160097-36421595	1.13788	4.45621	1.96947	2.06763	0.00025	0.00483727
FPR3	chr19:52298410-52329334	0.403928	1.57376	1.96204	1.66697	0.00155	0.0185989
LOC10192928	chr5:8839843-8881636	0.408587	1.58771	1.95824	1.36461	0.00225	0.024287
POU6F2	chr7:39017608-39504390	0.320835	1.2414	1.95206	1.57935	0.00055	0.00876569
PIK3AP1	chr10:98353068-98480279	1.55962	6.02623	1.95006	2.42167	0.00005	0.00136643
NAPSB	chr19:50837056-50848005	1.08372	4.15531	1.93896	1.58707	0.00235	0.024984
MT1A	chr16:56672577-56673999	1.67948	6.41731	1.93396	1.35506	0.0032	0.0313892
MT1L	chr16:56651372-56652730	2.83375	10.7897	1.92886	1.59582	0.00125	0.0158974

LILRA2	chr19:55084463-55099028	0.707625	2.69371	1.92854	1.91307	0.00065	0.00986869
IL32	chr16:3115312-3119668	0.660489	2.51173	1.92707	1.52471	0.00245	0.0257982
LAT2	chr7:73624086-73644164	2.58976	9.83357	1.9249	2.31932	0.00005	0.00136643
IL7R	chr5:35856976-35879705	0.19756	0.74814	1.92102	1.51078	0.0009	0.0126208
HCLS1	chr3:121350244-121379795	4.19833	15.863	1.91778	2.53138	0.00005	0.00136643
MT1G	chr16:56700652-56701977	9.54056	35.8977	1.91175	1.8945	0.0001	0.00238525
APOLD1	chr12:12878850-12944399	9.98825	37.5555	1.91072	2.95947	0.00005	0.00136643
APLNR	chr11:57001051-57004927	29.3669	110.044	1.90581	2.32463	0.00005	0.00136643
ST14	chr11:130029681-130080257	0.371447	1.38792	1.9017	1.63575	0.00085	0.0120733
CCR1	chr3:46243199-46249832	1.48966	5.54279	1.89563	2.06563	0.00005	0.00136643
ITGB2	chr21:46305867-46349595	4.99947	18.5252	1.88964	2.69091	0.00005	0.00136643
AZGP1	chr7:99564349-99573735	4.89563	18.0922	1.8858	1.9916	0.00005	0.00136643
HSPA7	chr1:161575848-161578341	0.758356	2.80139	1.88519	1.80269	0.0002	0.00407561
SLC14A1	chr18:43304091-43332485	17.5933	64.4296	1.8727	2.60649	0.00005	0.00136643
SH2D6	chr2:85661917-85664152	0.268459	0.981602	1.87044	1.2132	0.00365	0.0347776
STX11	chr6:144471653-144513076	0.147197	0.535939	1.86433	1.44405	0.0017	0.0198318
C1orf162	chr1:112016603-112021134	2.00601	7.30258	1.86408	1.81001	0.00005	0.00136643
MS4A7	chr11:60145957-60163426	2.27573	8.26517	1.86072	2.23085	0.00005	0.00136643
HIC1	chr17:1958392-1962981	0.531393	1.92198	1.85474	1.5735	0.0023	0.0246907
GJA4	chr1:35258598-35261348	1.35499	4.86979	1.84558	1.79725	0.00025	0.00483727
FAM95C	chr9:38540563-38545369	2.7524	9.88085	1.84395	2.14271	0.00005	0.00136643
MAFF	chr22:38597938-38612517	3.28422	11.7318	1.8368	1.92424	0.0007	0.010395
EMR2	chr19:14843508-14889353	0.375728	1.34191	1.83653	1.7617	0.0009	0.0126208
SERPINA3	chr14:95078713-95090390	69.246	247.139	1.83552	1.64019	0.0002	0.00407561
ADAMTS1	chr21:28208605-28217728	7.56065	26.9401	1.83317	2.77042	0.00005	0.00136643
DLL4	chr15:41221530-41231258	1.44877	5.15709	1.83173	2.08879	0.00005	0.00136643
MLKL	chr16:74705752-74734789	0.83735	2.97761	1.83025	1.83684	0.00055	0.00876569
TREM2	chr6:41126243-41130924	5.20302	18.4795	1.8285	2.16816	0.00005	0.00136643
ABCA6	chr17:67074846-67138015	4.04323	14.3577	1.82824	2.63292	0.00005	0.00136643
IL10RA	chr11:117857105-117872199	1.99971	7.09276	1.82656	2.3187	0.00005	0.00136643
GBP2	chr1:89571815-89591842	1.69696	6.00652	1.82358	2.28128	0.00005	0.00136643
ANKRD18A	chr9:38571360-38620360	4.09308	14.4631	1.82112	2.44581	0.00005	0.00136643
LRRC25	chr19:18501953-18508415	0.609305	2.15269	1.8209	1.68973	0.0002	0.00407561
C11orf21	chr11:2317506-2323143	0.0965213	0.33887	1.81181	1.23312	0.00305	0.0303571
CLDN15	chr7:100875372-100882101	1.93453	6.78604	1.81058	1.86731	0.0003	0.00551994
RAC2	chr22:37621300-37640339	0.855099	2.99742	1.80956	1.48776	0.00395	0.0368006
TLR7	chrX:12885201-12908480	1.1242	3.94059	1.80951	2.04633	0.00005	0.00136643
TPTEP1	chr22:17082800-17129720	1.11178	3.89693	1.80946	1.64454	0.00005	0.00136643
OR6W1P	chr7:142759380-142760882	0.243042	0.850866	1.80773	1.29186	0.00415	0.0381398
LINC00638	chr14:105287537-105290055	0.483163	1.69008	1.80651	1.45108	0.00415	0.0381398
C3AR1	chr12:8210918-8218955	3.23869	11.3175	1.80507	2.13957	0.00005	0.00136643
SLC27A6	chr5:128301209-128369335	1.96374	6.83312	1.79894	1.99626	0.0001	0.00238525
SIRPB2	chr20:1455235-1472233	0.594215	2.06599	1.79778	1.76239	0.0003	0.00551994
IL4R	chr16:27325229-27376099	2.84753	9.89562	1.79708	2.28484	0.00005	0.00136643
CCRL2	chr3:46448720-46451014	0.317103	1.09919	1.79341	1.42664	0.0038	0.0358579
LYZ	chr12:69742133-69748013	1.57894	5.46809	1.79208	1.68762	0.00045	0.00751151
CASP4	chr11:104813593-104839325	1.46939	5.08567	1.79122	1.75795	0.00035	0.00620045
SLC15A5	chr12:16341418-16430619	0.0841452	0.290376	1.78697	1.17734	0.00455	0.0407742

IL18	chr11:112013973-112034840	1.50541	5.1945	1.78683	1.64054	0.0002	0.00407561
ARHGAP9	chr12:57853917-57873633	0.963371	3.32303	1.78634	1.42215	0.00495	0.0433451
CXCR4	chr2:136871918-136875725	5.39717	18.5912	1.78434	2.06783	0.00005	0.00136643
MATN1-AS1	chr1:31184123-31199593	0.50425	1.73376	1.78169	1.3679	0.0053	0.0457508
PI16	chr6:36916038-36932613	3.34874	11.4989	1.77981	2.20495	0.00005	0.00136643
FCER1G	chr1:161185086-161189038	6.23425	21.3206	1.77396	1.71752	0.00055	0.00876569
ACSL5	chr10:114133915-114188138	2.38602	8.14953	1.77211	2.23395	0.00005	0.00136643
MS4A4A	chr11:60048013-60076445	1.53536	5.23812	1.77047	1.58408	0.0016	0.0190433
FAP	chr2:162970950-163100045	0.63826	2.17524	1.76896	1.62934	0.00035	0.00620045
ABCC2	chr10:101542354-101611949	0.176674	0.600607	1.76533	1.42542	0.0056	0.0474801
CD22	chr19:35820068-35838264	4.5377	15.4238	1.76513	2.4492	0.00005	0.00136643
FYB	chr5:39105353-39270759	1.86864	6.35131	1.76507	2.33677	0.00005	0.00136643
S100A9	chr1:153330329-153333503	5.15605	17.4906	1.76224	1.52353	0.00245	0.0257982
ITPR3	chr6:33589155-33664348	0.783293	2.65371	1.76039	2.2389	0.00005	0.00136643
CH25H	chr10:90965693-90967071	1.57684	5.31502	1.75304	1.6271	0.00015	0.00330506
CXCL2	chr4:74962753-74964997	1.24979	4.21041	1.75228	1.51481	0.0006	0.00935947
TNFRSF10A	chr8:23048969-23082680	0.457784	1.53399	1.74455	1.39356	0.0039	0.0365472
A4GALT	chr22:43088126-43116876	1.62109	5.41383	1.73968	1.75801	0.00035	0.00620045
EMP1	chr12:13349601-13369708	7.6005	25.3627	1.73855	2.47578	0.00005	0.00136643
TBX3	chr12:115108058-115121969	0.753204	2.51149	1.73743	1.72723	0.0016	0.0190433
PTGS2	chr1:186640943-186649559	0.918267	3.05466	1.73403	1.69747	0.001	0.0136643
CD44	chr11:35160416-35253949	14.7849	48.9641	1.7276	2.43525	0.00005	0.00136643
CFI	chr4:110661847-110723335	2.1322	7.05768	1.72685	1.92328	0.00005	0.00136643
LINC00601	chr10:128102437-128110448	0.956117	3.15123	1.72066	1.35144	0.00565	0.0476976
IKZF1	chr7:50343678-50472798	1.21226	3.96444	1.70942	1.67346	0.00005	0.00136643
FENDRR	chr16:86508130-86542466	0.509285	1.66329	1.7075	1.4709	0.0021	0.0232225
TNFRSF1B	chr1:12226999-12269277	3.14343	10.2566	1.70614	2.34356	0.00005	0.00136643
LOC729737	chr1:134772-140566	0.273369	0.891617	1.70558	1.57422	0.0003	0.00551994
LINC01338	chr5:82146683-82155655	2.97057	9.68429	1.7049	1.38297	0.006	0.0498407
CD53	chr1:111413820-111442558	5.04089	16.4131	1.7031	2.12341	0.00005	0.00136643
RNASE6	chr14:21249209-21250626	2.58167	8.39031	1.70042	1.64139	0.0008	0.0115682
SLC17A9	chr20:61583998-61599949	0.50167	1.62789	1.69819	1.38069	0.00485	0.0427669
FLT1	chr13:28874482-29069265	12.4862	40.4004	1.69404	2.63051	0.00005	0.00136643
SPN	chr16:29674270-29681823	0.484118	1.56265	1.69056	1.82536	0.00005	0.00136643
MS4A14	chr11:60163486-60185228	0.429817	1.3841	1.68716	1.59796	0.0007	0.010395
CLEC2B	chr12:10004967-10022458	0.924146	2.97	1.68427	1.5693	0.00045	0.00751151
TEAD4	chr12:3068477-3149842	0.444837	1.42763	1.68227	1.31607	0.00365	0.0347776
SH3TC1	chr4:8201059-8242830	1.63818	5.23817	1.67697	2.0981	0.00005	0.00136643
ITPRIPL1	chr2:96991061-96994091	0.253829	0.810434	1.67484	1.38592	0.0045	0.0404495
CPM	chr12:69244955-69357020	2.8174	8.98356	1.67292	2.57202	0.00005	0.00136643
C5AR1	chr19:47813103-47825327	1.29109	4.11271	1.67149	1.64132	0.0004	0.00687883
TCAP	chr17:37821598-37822807	1.22506	3.88155	1.66378	1.35114	0.00555	0.0473065
HPR	chr16:72097124-72111145	1.94641	6.1495	1.65965	1.63806	0.0007	0.010395
NLRC5	chr16:57050985-57117436	0.899046	2.83797	1.65839	2.08384	0.00005	0.00136643
SLC7A7	chr14:23242431-23289020	1.3764	4.33773	1.65604	1.71606	0.0006	0.00935947
MYOT	chr5:137203544-137223540	1.48336	4.67231	1.65527	1.7605	0.0003	0.00551994
ADORA3	chr1:112025969-112106597	5.12777	16.0261	1.64402	2.03262	0.00005	0.00136643
APOBR	chr16:28505969-28510291	0.375215	1.17238	1.64365	1.5297	0.00075	0.0109258

TLR9	chr3:52255095-52260179	0.207067	0.642916	1.63453	1.25814	0.00315	0.0310023
ATF3	chr1:212738675-212794119	1.81954	5.63853	1.63175	1.84747	0.0004	0.00687883
TMEM102	chr17:7338761-7340998	0.330111	1.02238	1.63091	1.25742	0.0055	0.0470395
SASH3	chrX:128913891-128929176	1.336	4.13445	1.62978	1.81897	0.0002	0.00407561
SLC2A5	chr1:9097004-9129887	6.17792	19.1133	1.62939	2.1614	0.00005	0.00136643
SLC19A3	chr2:228549925-228582745	1.1491	3.54198	1.62406	1.81904	0.00005	0.00136643
AKR1C2	chr10:5029967-5060225	1.23183	3.79188	1.62211	1.49847	0.0017	0.0198318
RHBDF2	chr17:74466974-74497509	3.45791	10.6301	1.62019	2.23307	0.00005	0.00136643
TMPRSS5	chr11:113558267-113577095	4.30561	13.2265	1.61914	1.95601	0.00035	0.00620045
C1orf64	chr1:16330730-16333184	5.43035	16.5901	1.61121	1.83688	0.0002	0.00407561
OSGIN1	chr16:83986826-83999937	1.24434	3.79688	1.60944	1.54927	0.00085	0.0120733
PIDD1	chr11:799178-805250	2.20348	6.7058	1.60563	1.85459	0.0001	0.00238525
CXorf36	chrX:45007617-45060146	2.27966	6.93175	1.6044	1.99881	0.00005	0.00136643
OLR1	chr12:10310898-10344403	2.80237	8.49265	1.59957	1.86317	0.0001	0.00238525
LCP1	chr13:46700057-46756459	3.06546	9.27283	1.59691	2.19818	0.00005	0.00136643
SPI1	chr11:47376408-47400127	2.77061	8.35069	1.59169	1.66314	0.0014	0.0173787
STAB1	chr3:52529355-52569093	4.35585	13.1056	1.58916	2.00839	0.00005	0.00136643
RGR	chr10:86004808-86018944	4.54811	13.6737	1.58806	1.78531	0.0011	0.0145132
CDA	chr1:20915443-20945400	0.603278	1.81135	1.58617	1.18741	0.0054	0.0463417
ADM	chr11:10326641-10328923	6.7173	20.1592	1.58548	1.64944	0.00215	0.0235978
MYO1F	chr19:8585673-8642331	1.67419	5.00553	1.58006	1.97443	0.00005	0.00136643
ARHGAP30	chr1:161016731-161039760	1.17241	3.50215	1.57876	1.7591	0.0012	0.015451
APOC2	chr19:45445494-45452822	5.57537	16.6388	1.57741	1.69516	0.00025	0.00483727
SMTN	chr22:31477281-31500610	1.96184	5.85106	1.57649	1.82205	0.0004	0.00687883
SLC37A2	chr11:124933012-124960412	0.85848	2.55444	1.57315	1.66692	0.0001	0.00238525
BCL6	chr3:187416046-187463513	13.2492	39.3827	1.57166	2.59184	0.00005	0.00136643
BAG3	chr10:121410881-121437329	12.7605	37.9159	1.57112	2.47956	0.00005	0.00136643
CEBPD	chr8:48649475-48650726	10.9699	32.552	1.56919	2.00969	0.00005	0.00136643
RIN3	chr14:92980124-93155334	1.87557	5.55827	1.5673	1.98135	0.00005	0.00136643
ALOX5AP	chr13:31287614-31338565	4.36019	12.91	1.56602	1.64693	0.0003	0.00551994
WAS	chrX:48542185-48549817	1.00311	2.96698	1.56451	1.53473	0.0011	0.0145132
PIK3R5	chr17:8782232-8869029	0.988755	2.9231	1.56381	1.76975	0.00155	0.0185989
HS3ST4	chr16:25703346-26149009	4.74929	13.9418	1.55364	2.1619	0.00005	0.00136643
CLCA4	chr1:87012758-87046432	2.69217	7.90183	1.55342	1.97874	0.00005	0.00136643
CASP1	chr11:104896236-104905884	1.58678	4.6546	1.55256	1.50241	0.00125	0.0158974
LGALS9	chr17:25958173-25976586	2.95737	8.67056	1.55181	1.80117	0.0004	0.00687883
ANLN	chr7:36363758-36493401	24.816	72.7379	1.55143	2.10603	0.00005	0.00136643
LOXL2	chr8:23154409-23261722	0.484969	1.41985	1.54977	1.35124	0.00595	0.0496124
S100A4	chr1:153516094-153518282	3.92166	11.4488	1.54566	1.40394	0.003	0.0299609
C10orf128	chr10:50362768-50396445	2.39489	6.98943	1.54522	1.82725	0.0009	0.0126208
TMC6	chr17:76108998-76139049	7.27168	21.101	1.53695	2.04086	0.00005	0.00136643
EHD2	chr19:48216600-48246391	3.21956	9.32892	1.53484	2.05299	0.00005	0.00136643
SRPK3	chrX:153046455-153051187	0.680993	1.97132	1.53345	1.42459	0.0029	0.0292774
PIEZO2	chr18:10670243-11148761	3.34858	9.64807	1.52669	2.34782	0.00005	0.00136643
ID3	chr1:23884420-23886285	19.8042	57.0431	1.52625	2.22161	0.00005	0.00136643
DOCK6	chr19:11309968-11373168	1.42974	4.11236	1.52422	1.95714	0.00005	0.00136643
TMEM140	chr7:134832765-134855578	2.55844	7.35187	1.52285	1.30117	0.0049	0.0430141
C10orf11	chr10:77542518-78317126	2.05591	5.88665	1.51767	1.43373	0.0019	0.0216212

PRAM1	chr19:8554939-8567538	1.00191	2.86674	1.51666	1.48407	0.002	0.0224405
CD86	chr3:121774208-121839988	1.22432	3.49424	1.51299	1.56503	0.00235	0.024984
TBXAS1	chr7:139478046-139720125	3.20467	9.10757	1.50689	1.79961	0.001	0.0136643
DKFZp451B08	chr6:163759373-163768065	1.02852	2.91944	1.50513	1.69705	0.00055	0.00876569
ITGAL	chr16:30483982-30534506	0.569474	1.61158	1.50078	1.54743	0.00105	0.0140962
LOC10028965	chr19:43326014-43331030	0.815221	2.29321	1.49211	1.3545	0.0011	0.0145132
PI4KAP1	chr22:20383730-20398695	1.53234	4.30833	1.49139	1.55873	0.00055	0.00876569
PGAM2	chr7:44102325-44105186	8.62947	24.2526	1.49079	1.77902	0.0002	0.00407561
COL7A1	chr3:48601505-48632593	1.06936	3.00415	1.49021	1.92396	0.00005	0.00136643
TCIRG1	chr11:67806461-67818366	2.46283	6.91776	1.48999	1.68119	0.0004	0.00687883
CSF2RB	chr22:37309674-37336479	0.577669	1.62093	1.48851	1.4526	0.00265	0.0273487
CD68	chr17:7482804-7485429	4.88666	13.6937	1.48659	1.91759	0.00005	0.00136643
MYC	chr8:128748314-128753680	1.61312	4.51632	1.48529	1.62633	0.00015	0.00330506
CRYAB	chr11:111779343-111797595	164.764	460.803	1.48375	2.2851	0.00005	0.00136643
EFHD1	chr2:233470766-233547491	30.9576	86.5501	1.48324	2.31048	0.00005	0.00136643
PLSCR1	chr3:146232966-146262628	5.31505	14.8295	1.48031	1.96858	0.0001	0.00238525
ACKR1	chr1:159173802-159176290	5.77495	16.1066	1.47977	1.7615	0.00035	0.00620045
HMOX1	chr22:35777059-35790207	6.18189	17.24	1.47964	1.88504	0.00005	0.00136643
COL27A1	chr9:116918230-117072975	1.44738	4.03626	1.47958	1.76406	0.0001	0.00238525
LRRC63	chr13:46786077-46850938	1.06174	2.95952	1.47894	1.42249	0.0022	0.0239231
PRX	chr19:40899670-40919271	1.25873	3.50475	1.47734	1.79571	0.00035	0.00620045
MT1X	chr16:56716381-56718108	50.6724	141.067	1.47711	2.09186	0.00005	0.00136643
SBNO2	chr19:1107632-1174282	2.51192	6.98973	1.47644	1.97044	0.0002	0.00407561
LRRC32	chr11:76368567-76381791	3.0732	8.55133	1.47641	1.8661	0.00035	0.00620045
MYBPC1	chr12:101988708-102079658	5.07371	14.1119	1.4758	2.30299	0.00005	0.00136643
C1QTNF6	chr22:37576205-37584330	0.768753	2.13768	1.47545	1.28932	0.0045	0.0404495
FAM107A	chr3:58549838-58613337	317.635	883.2	1.47537	1.47896	0.0024	0.0253778
MT2A	chr16:56642477-56643409	109.66	304.675	1.47424	2.35713	0.00005	0.00136643
MARCH3	chr5:126203405-126366500	1.82145	5.04834	1.47072	1.77633	0.0001	0.00238525
HERC2P2	chr15:23282264-23378259	12.0466	33.3369	1.4685	2.46614	0.00005	0.00136643
NOS3	chr7:150688143-150721586	1.3888	3.82952	1.46333	1.47333	0.003	0.0299609
ITGB4	chr17:73717515-73753899	21.6039	59.4682	1.46083	2.21815	0.00005	0.00136643
P2RX7	chr12:121570621-121624354	10.1588	27.9526	1.46025	2.24801	0.00005	0.00136643
FES	chr15:91427664-91439006	1.76189	4.84291	1.45875	1.76332	0.0005	0.00814556
KCNJ2	chr17:68165675-68176183	6.32542	17.3754	1.45782	2.35809	0.00005	0.00136643
JAK3	chr19:17935592-17958841	1.78248	4.89624	1.45779	1.90594	0.00005	0.00136643
TNFAIP3	chr6:138144806-138204451	1.16057	3.18413	1.45607	1.72101	0.0007	0.010395
GADD45G	chr9:92219926-92221469	2.42121	6.63928	1.4553	1.36683	0.0017	0.0198318
MUC1	chr1:155158299-155162706	0.708502	1.93901	1.45248	1.15843	0.006	0.0498407
NFKBIA	chr14:35870715-35873960	22.368	61.1681	1.45134	2.31958	0.00005	0.00136643
PTAFR	chr1:28473676-28520447	1.94651	5.32009	1.45056	1.82767	0.00015	0.00330506
LINC00910	chr17:41447212-41466266	0.712993	1.94815	1.45014	1.36373	0.0022	0.0239231
PLIN4	chr19:4472192-4517716	2.89012	7.8764	1.44641	1.43125	0.00415	0.0381398
SLC26A2	chr5:149340299-149366963	2.99446	8.15909	1.44612	2.09515	0.00005	0.00136643
PRKX	chrX:3522383-3631675	4.38419	11.9263	1.44377	2.18274	0.00005	0.00136643
RNF144B	chr6:18387580-18469105	3.69484	10.0411	1.44233	2.09402	0.00005	0.00136643
UNC13D	chr17:73823307-73840798	1.05014	2.83903	1.43482	1.62534	0.00065	0.00986869
ADAMTS9	chr3:64501330-64997143	2.74158	7.40998	1.43446	1.78169	0.0003	0.00551994

MECOM	chr3:168801286-169381563	1.74146	4.69483	1.43077	1.87345	0.00005	0.00136643
MT1F	chr16:56691854-56693215	27.0974	73.0066	1.42987	1.73218	0.00025	0.00483727
HAVCR2	chr5:156512842-156536248	3.50825	9.43531	1.42732	1.76516	0.0004	0.00687883
LCP2	chr5:169675087-169724822	1.02138	2.74567	1.42663	1.38638	0.0045	0.0404495
SEPP1	chr5:42756919-42812024	82.9446	222.292	1.42224	2.19312	0.00005	0.00136643
C10orf54	chr10:73156690-73575704	9.57607	25.6632	1.4222	1.49702	0.00135	0.0168292
BTK	chrX:100604434-100645784	0.830844	2.21575	1.41515	1.40855	0.0022	0.0239231
PECAM1	chr17:62396776-62407083	3.6583	9.74233	1.41309	1.92311	0.00005	0.00136643
SYK	chr9:93563961-93660842	2.20257	5.86334	1.41254	1.91102	0.0001	0.00238525
ISLR	chr15:74466086-74469212	3.92206	10.4353	1.41179	1.74696	0.0002	0.00407561
NFKB2	chr10:104153866-104162286	1.51964	4.04219	1.41141	1.58657	0.00145	0.0177865
DRAXIN	chr1:11751780-11780336	1.33287	3.53975	1.40911	1.30841	0.00265	0.0273487
EMP3	chr19:48828628-48833810	7.9271	20.9853	1.40452	1.56135	0.00145	0.0177865
SLC6A12	chr12:299242-323371	4.78766	12.6743	1.40451	1.95969	0.00015	0.00330506
ENTPD2	chr9:139942550-139948503	4.39997	11.62	1.40105	1.62227	0.0005	0.00814556
TGFBR1	chr9:101867411-101916473	6.57448	17.3436	1.39945	2.14151	0.00005	0.00136643
TRAF1	chr9:123664670-123691451	2.73547	7.20944	1.3981	1.87213	0.00015	0.00330506
PAPSS2	chr10:89419475-89507462	2.77943	7.31774	1.39661	1.74376	0.0002	0.00407561
PRKY	chrY:7142012-7249588	0.933301	2.45679	1.39636	1.73815	0.0002	0.00407561
FKBP5	chr6:35541361-35704724	22.0406	57.997	1.39582	1.79108	0.0012	0.015451
RARRES3	chr11:63014620-63330855	13.3238	35.0508	1.39545	1.37693	0.00235	0.024984
PTP4A3	chr8:142431487-142442554	4.05666	10.6333	1.39022	1.70299	0.00075	0.0109258
MAMDC4	chr9:139746818-139755251	0.975455	2.55609	1.38979	1.31413	0.00485	0.0427669
LIME1	chr20:62367977-62370460	2.12071	5.54608	1.38692	1.25822	0.00395	0.0368006
NEAT1	chr11:65190268-65194003	114.578	299.485	1.38616	1.29616	0.0016	0.0190433
CDH19	chr18:64168423-64271375	3.09824	8.07313	1.38168	2.03829	0.00005	0.00136643
CHST3	chr10:73724119-73773322	6.32139	16.4663	1.3812	2.07283	0.00005	0.00136643
FXYD5	chr19:35645624-35660788	2.69988	7.0291	1.38044	1.37194	0.0043	0.0391105
BOK	chr2:242483800-242513553	19.5902	50.9929	1.38016	2.07519	0.00005	0.00136643
PPAP2C	chr19:281039-291435	10.1433	26.3711	1.37843	1.80468	0.0003	0.00551994
NKX6-2	chr10:134598319-134599537	14.8042	38.4717	1.3778	1.64254	0.0003	0.00551994
HELZ2	chr20:62189438-62205592	0.825549	2.14456	1.37726	1.69512	0.0001	0.00238525
MAN2A1	chr5:109025066-109205326	30.31	78.7296	1.37711	2.06821	0.0001	0.00238525
KLF15	chr3:126061477-126076236	11.5394	29.9643	1.37667	2.08786	0.00005	0.00136643
RAMP3	chr7:45197366-45223850	4.96631	12.8913	1.37615	1.6498	0.0003	0.00551994
IL6R	chr1:154377668-154441926	2.59524	6.7262	1.37393	1.90557	0.00005	0.00136643
ZFP36L1	chr14:69254371-69262960	26.5431	68.7564	1.37316	2.27591	0.00005	0.00136643
PTCHD1	chrX:23352984-23414918	3.61823	9.37174	1.37303	1.97439	0.00005	0.00136643
CTSS	chr1:150702671-150738433	3.26452	8.44947	1.37199	1.93162	0.00005	0.00136643
CIITA	chr16:10971054-11018840	1.94635	5.03695	1.37178	1.69167	0.0006	0.00935947
RASAL3	chr19:15562437-15575382	0.708746	1.83059	1.36897	1.29324	0.00555	0.0473065
FANCC	chr9:97861335-98079991	0.96109	2.48203	1.36878	1.45479	0.0024	0.0253778
PALM3	chr19:14164178-14169971	0.643458	1.66171	1.36875	1.27656	0.00365	0.0347776
MID1IP1	chrX:38660500-38665783	31.6282	81.5789	1.36699	2.13753	0.00005	0.00136643
DNAJB1	chr19:14625581-14629201	34.3076	88.3532	1.36476	2.18102	0.00005	0.00136643
MMRN1	chr4:90816051-90875780	1.0686	2.75159	1.36454	1.31392	0.0042	0.0384589
KANK2	chr19:11274942-11308243	5.28888	13.6093	1.36355	2.0243	0.0001	0.00238525
TLR6	chr4:38825328-38858438	0.98982	2.54526	1.36257	1.64398	0.0001	0.00238525

SLC7A2	chr8:17354596-17428077	10.7485	27.6119	1.36116	2.01733	0.00005	0.00136643
MKNK2	chr19:2037469-2051243	13.0883	33.6075	1.36051	1.88381	0.00005	0.00136643
HK2	chr2:75059781-75120481	2.25174	5.7812	1.36033	1.94297	0.0001	0.00238525
IRF8	chr16:85932773-85956211	2.55268	6.53765	1.35676	1.59391	0.0003	0.00551994
OSMR	chr5:38845959-38935743	4.68026	11.9825	1.35627	1.59197	0.00065	0.00986869
SLC38A2	chr12:46751970-46766645	38.745	99.0962	1.35482	2.16911	0.00005	0.00136643
FGL2	chr7:76751933-76924521	3.79052	9.65037	1.34819	1.35839	0.00465	0.0415015
NR4A2	chr2:157180943-157189287	1.82309	4.64141	1.34818	1.47294	0.00125	0.0158974
SRGN	chr10:70847827-70864567	22.5239	57.3002	1.34708	1.88853	0.00005	0.00136643
BMF	chr15:40380090-40401085	0.679195	1.72488	1.3446	1.36715	0.0059	0.0492421
SIPA1	chr11:65405577-65418391	3.35097	8.50607	1.34392	1.83709	0.00015	0.00330506
TYROBP	chr19:36395302-36399211	9.87256	25.0325	1.34231	1.48255	0.0027	0.0277348
SOX7	chr8:10581277-10588084	0.790433	2.00175	1.34055	1.30753	0.0043	0.0391105
STARD8	chrX:67867510-67945684	1.97506	4.99614	1.33892	1.75292	0.0002	0.00407561
ABCA8	chr17:66863427-66951533	12.348	31.233	1.33879	2.08467	0.00005	0.00136643
GRM4	chr6:33986419-34123399	0.60312	1.52504	1.33833	1.47453	0.00145	0.0177865
IRAK3	chr12:66582977-66648394	1.53396	3.87446	1.33673	1.82465	0.00025	0.00483727
CD84	chr1:160510883-160549306	1.36159	3.43666	1.33571	1.79805	0.00005	0.00136643
ITPKB	chr1:226819390-226926876	65.3119	164.83	1.33556	1.74845	0.0001	0.00238525
MICALL2	chr7:1473994-1499109	5.58239	14.0744	1.33412	1.84354	0.0001	0.00238525
ASB4	chr7:95115212-95169543	0.752002	1.89138	1.33063	1.31316	0.004	0.0371878
PYGL	chr14:51371934-51411248	3.4794	8.74573	1.32974	1.78318	0.00015	0.00330506
PLIN2	chr9:19115758-19127604	4.99474	12.5523	1.32947	1.77959	0.0002	0.00407561
DOCK2	chr5:169064250-169510386	2.13543	5.36151	1.32811	1.64161	0.001	0.0136643
DENND3	chr8:142138719-142205900	5.75399	14.446	1.32803	2.10697	0.00005	0.00136643
DSC2	chr18:28645941-28742819	1.57227	3.94706	1.32793	1.58587	0.0015	0.0182223
IFI16	chr1:158979681-159024945	7.11733	17.8658	1.32779	2.02319	0.00005	0.00136643
CD34	chr1:208059882-208084683	2.82062	7.06719	1.32512	1.58583	0.00075	0.0109258
ATOH8	chr2:85980908-86018506	1.78581	4.47429	1.32509	1.67991	0.0003	0.00551994
ERBB2IP	chr5:65222381-65376851	49.3417	123.548	1.32419	1.77035	0.0002	0.00407561
MT1M	chr16:56666533-56667898	30.8259	77.0979	1.32255	1.72427	0.0002	0.00407561
TRIP10	chr19:6739692-6751537	2.90201	7.2417	1.31928	1.49007	0.0021	0.0232225
NFIL3	chr9:94171326-94186908	3.86223	9.62444	1.31727	1.71744	0.0003	0.00551994
RFTN2	chr2:198435526-198540584	18.8398	46.8607	1.3146	2.2233	0.00005	0.00136643
LRP4	chr11:46867961-46940173	25.2034	62.6738	1.31424	1.9312	0.00005	0.00136643
LAPTM5	chr1:31205314-31230683	15.6102	38.7992	1.31353	2.00277	0.00005	0.00136643
POU2F2	chr19:42590261-42636625	0.710866	1.76536	1.31231	1.23414	0.0031	0.0306643
GALNT15	chr3:16216183-16271253	23.6271	58.6394	1.31143	2.00098	0.00005	0.00136643
DTX3L	chr3:122246759-122294049	3.39324	8.42125	1.31137	1.55173	0.0007	0.010395
PRIMA1	chr14:94184643-94254766	1.549	3.84128	1.31025	1.43686	0.00235	0.024984
ATHL1	chr11:289137-295688	1.88765	4.66911	1.30656	1.47126	0.00235	0.024984
INPP5D	chr2:233924676-234116549	3.83958	9.48767	1.30511	1.97682	0.00005	0.00136643
CYTIP	chr2:158271130-158300604	0.706742	1.74564	1.3045	1.1961	0.0052	0.0449757
MFNG	chr22:37865100-37882478	1.68101	4.1507	1.30402	1.38284	0.0022	0.0239231
TSHZ3	chr19:31765850-31840190	2.25219	5.56003	1.30376	1.73562	0.00015	0.00330506
LINC01354	chr1:234663636-234667525	3.11338	7.65448	1.29782	1.57733	0.0011	0.0145132
PLIN1	chr15:90207599-90222648	5.11726	12.5778	1.29744	1.76036	0.00015	0.00330506
PTH1R	chr3:46919235-46945289	3.54012	8.70072	1.29734	1.52959	0.0006	0.00935947

TEP1	chr14:20833825-20881579	2.47639	6.08316	1.29658	2.04343	0.00005	0.00136643
BAZ1A	chr14:35221936-35344853	2.17842	5.34739	1.29555	1.82985	0.00025	0.00483727
NCKAP1L	chr12:54891494-54936899	2.75336	6.75802	1.29541	1.78741	0.00005	0.00136643
IKZF2	chr2:213864410-214016333	4.08906	10.0344	1.29511	2.08288	0.00005	0.00136643
GPR133	chr12:131438451-131626008	0.769735	1.88786	1.29432	1.36265	0.00245	0.0257982
CD74	chr5:149781199-149792499	84.718	207.267	1.29075	1.9077	0.00005	0.00136643
RASL12	chr15:65337707-65360388	10.7556	26.268	1.28822	1.98281	0.00005	0.00136643
SERPINH1	chr11:75273100-75283849	5.65592	13.78	1.28475	1.71271	0.0006	0.00935947
HAP1	chr17:39878890-39890898	3.58698	8.73217	1.28357	1.7549	0.00005	0.00136643
TAL1	chr1:47681961-47698007	1.64816	4.01068	1.283	1.49206	0.0021	0.0232225
CPLX3	chr15:75118950-75124136	2.21388	5.36549	1.27713	1.38712	0.0022	0.0239231
TLR1	chr4:38797875-38806412	2.59378	6.28029	1.27578	1.53399	0.00095	0.0131856
SLCO2B1	chr11:74862031-74917445	16.3311	39.4522	1.27249	2.14054	0.00005	0.00136643
FANCB	chrX:14861528-14891184	3.50576	8.45632	1.2703	1.72844	0.0001	0.00238525
FGD2	chr6:36973422-36996845	2.18375	5.26437	1.26945	1.56358	0.0007	0.010395
SLC5A11	chr16:24857183-24922949	5.09455	12.2813	1.26944	1.51406	0.00125	0.0158974
GAB3	chrX:153903526-153979858	0.886626	2.13675	1.26902	1.44546	0.00215	0.0235978
FGFRL1	chr4:1005609-1020686	6.96372	16.7805	1.26886	1.91565	0.00005	0.00136643
C10orf90	chr10:128113573-128210010	5.78557	13.927	1.26736	1.85749	0.00005	0.00136643
C18orf54	chr18:51884286-51908405	1.09831	2.64275	1.26676	1.47838	0.00225	0.024287
MAFIP	chr4_gl000194_random:53588-11!	5.86228	14.0524	1.26129	1.54796	0.00085	0.0120733
GLI2	chr2:121554866-121750229	0.522223	1.24997	1.25916	1.3315	0.0026	0.0269589
SHROOM1	chr5:132157832-132166590	3.91119	9.35783	1.25857	1.63432	0.0004	0.00687883
IFITM1	chr11:313990-315272	37.5657	89.8743	1.25849	1.76488	0.00025	0.00483727
PIRT	chr17:10725791-10741418	4.65953	11.115	1.25426	1.75025	0.0001	0.00238525
GYPC	chr2:127413510-127454251	4.05923	9.643	1.24827	1.35798	0.00495	0.0433451
PLSCR4	chr3:145910123-145968966	18.4075	43.6661	1.24622	2.01604	0.00005	0.00136643
PIK3CG	chr7:106505722-106549423	0.915647	2.17134	1.24572	1.38849	0.00295	0.0296802
PLEC	chr8:144989314-145050913	33.3271	78.7472	1.24053	1.39592	0.0025	0.0262151
C1R	chr12:7187514-7245043	9.71313	22.9462	1.24025	1.71039	0.0002	0.00407561
CMAHP	chr6:25081294-25138620	1.73898	4.10678	1.23977	1.27999	0.00555	0.0473065
PODN	chr1:53527723-53551174	1.48805	3.51324	1.23938	1.38133	0.00395	0.0368006
SEMA3E	chr7:82993221-83278479	1.7663	4.17014	1.23937	1.6003	0.00065	0.00986869
RELL1	chr4:37455551-37687999	6.73339	15.8873	1.23847	1.62064	0.00045	0.00751151
PIEZO1	chr16:88772890-88851372	4.36463	10.2914	1.23751	1.78249	0.00015	0.00330506
LINC00320	chr21:22114907-22175426	14.2368	33.5513	1.23674	1.85043	0.00005	0.00136643
FLI1	chr11:128556429-128683162	2.37938	5.59703	1.23407	1.54868	0.00105	0.0140962
PIP4K2A	chr10:22823765-23003503	54.2948	127.4	1.23048	1.89936	0.0002	0.00407561
LAMA5	chr20:60884115-60942368	3.57623	8.37126	1.227	1.78234	0.00005	0.00136643
ABCA1	chr9:107543283-107690527	10.4133	24.1518	1.2137	1.97467	0.00005	0.00136643
GRIK3	chr1:37261127-37499844	3.31672	7.69146	1.2135	1.86927	0.0001	0.00238525
ARRDC2	chr19:18111940-18124911	13.322	30.8654	1.21217	1.84768	0.00005	0.00136643
ADARB2	chr10:1223252-1779670	5.99502	13.8833	1.21151	1.97007	0.00005	0.00136643
ARHGEF10	chr8:1772148-1906807	9.18541	21.2698	1.21139	1.99057	0.00005	0.00136643
GPIHBP1	chr8:144295067-144299044	4.52631	10.4791	1.21111	1.4478	0.0007	0.010395
SHROOM4	chrX:50334642-50557044	4.41691	10.1793	1.20453	1.81173	0.00005	0.00136643
LOC389765	chr9:88420916-88457794	5.28476	12.1705	1.20348	1.5967	0.0004	0.00687883
RAB3IL1	chr11:61664767-61687741	2.51491	5.76068	1.19573	1.4396	0.0019	0.0216212

KIAA1755	chr20:36838906-36917348	4.39924	10.0702	1.19476	1.82979	0.00005	0.00136643
CAPG	chr2:85621870-85641197	10.5509	24.1338	1.19369	1.62676	0.0009	0.0126208
TRIM59	chr3:160153290-160167626	5.0245	11.4928	1.19368	1.7333	0.00005	0.00136643
C1S	chr12:7167979-7178335	10.8393	24.7789	1.19284	1.68043	0.0002	0.00407561
PLXND1	chr3:129274055-129325582	7.01622	16.033	1.19227	1.9516	0.00005	0.00136643
GBP3	chr1:89472359-89488549	1.95834	4.47123	1.19104	1.40669	0.00175	0.0203076
CD93	chr20:23059992-23066977	3.25325	7.42423	1.19036	1.43046	0.0045	0.0404495
UAP1L1	chr9:139971952-139978990	4.13718	9.41491	1.1863	1.57161	0.0009	0.0126208
SLC9A9	chr3:142984063-143567373	8.10598	18.4456	1.18622	1.79927	0.00015	0.00330506
F13A1	chr6:6144310-6320924	4.50616	10.2422	1.18455	1.31614	0.00575	0.048449
HAPLN2	chr1:156589085-156595517	12.8663	29.186	1.18168	1.54608	0.00125	0.0158974
TLE4	chr9:82186687-82341796	11.3435	25.7293	1.18155	1.95299	0.00005	0.00136643
NPNT	chr4:106816596-106892828	2.88592	6.52647	1.17727	1.52161	0.00125	0.0158974
ANKRD18B	chr9:33524410-33573001	1.27875	2.88457	1.17363	1.26529	0.00555	0.0473065
PRKD3	chr2:37477645-37544222	5.44498	12.2725	1.17243	1.87762	0.00005	0.00136643
ADIPOR2	chr12:1800246-1897845	28.933	65.1782	1.17168	1.92096	0.0001	0.00238525
BARD1	chr2:215590369-215674435	3.00122	6.74132	1.16748	1.69685	0.00015	0.00330506
AMER2	chr13:25735816-25746421	43.847	98.3804	1.16589	1.63011	0.00085	0.0120733
FOXO4	chrX:70315998-70323384	15.358	34.3862	1.16284	1.90173	0.00005	0.00136643
TSPO	chr22:43547519-43559248	12.106	27.0933	1.16222	1.36652	0.00415	0.0381398
ARL6IP6	chr2:153574406-153617767	5.30315	11.8641	1.16169	1.45746	0.00275	0.0281336
CDC42EP2	chr11:65082288-65089900	6.35353	14.2128	1.16156	1.52114	0.001	0.0136643
GAB1	chr4:144257982-144395718	15.9407	35.6225	1.16007	1.95063	0.00005	0.00136643
BACE2	chr21:42539727-42654461	1.76594	3.9391	1.15743	1.49861	0.002	0.0224405
SALL1	chr16:51169885-51185183	6.82517	15.2183	1.15687	1.88821	0.00015	0.00330506
QDPR	chr4:17488015-17513857	119.398	266.171	1.15657	1.63239	0.0015	0.0182223
MTHFD2	chr2:74425689-74442424	4.90378	10.9247	1.15563	1.28849	0.00535	0.0460471
USP54	chr10:75257295-75335433	16.7751	37.331	1.15405	1.90476	0.00005	0.00136643
MTSS1L	chr16:70695106-70719954	81.9669	182.256	1.15286	1.56814	0.00015	0.00330506
OSBPL11	chr3:125247701-125314381	10.4726	23.2818	1.15258	1.85429	0.00005	0.00136643
BGN	chrX:152713122-152775004	9.21675	20.4884	1.15248	1.41794	0.00345	0.0333771
SIK1	chr21:44834397-44847002	3.42392	7.60389	1.15109	1.35074	0.0058	0.048777
CTSC	chr11:88026759-88070941	5.90101	13.0923	1.14968	1.44983	0.0018	0.0207648
SLC45A3	chr1:205626980-205649630	4.09783	9.08533	1.14868	1.50509	0.00085	0.0120733
ELF1	chr13:41506054-41593508	5.70788	12.653	1.14845	1.71483	0.00035	0.00620045
ALOX5	chr10:45869623-45941567	2.91267	6.45218	1.14744	1.42088	0.00215	0.0235978
PRKCH	chr14:61788514-62017698	3.15836	6.98763	1.14563	1.53484	0.00075	0.0109258
ROBO3	chr11:124735304-124751370	1.47616	3.26544	1.14543	1.36925	0.00155	0.0185989
TRPV3	chr17:3413795-3461289	2.31716	5.11973	1.14371	1.51691	0.00115	0.0149934
ADAMTS10	chr19:8645123-8675620	3.43569	7.57358	1.14038	1.49284	0.0006	0.00935947
TRIM56	chr7:100728785-100733889	4.23852	9.33129	1.13852	1.53238	0.00105	0.0140962
TMEM176B	chr7:150488375-150502208	11.8477	26.0826	1.13848	1.32868	0.0033	0.0321732
ERAP2	chr5:96211643-96255406	1.38135	3.03848	1.13727	1.43725	0.0034	0.0330021
RENBP	chrX:153200721-153210232	4.83496	10.6208	1.13531	1.35369	0.00195	0.0220762
DYSF	chr2:71680752-71913893	3.7047	8.12986	1.13387	1.63827	0.0012	0.015451
JAKMIP3	chr10:133918312-133998313	6.18265	13.5623	1.13331	1.76217	0.0002	0.00407561
PLXNB3	chrX:153029650-153044801	9.89413	21.6983	1.13294	1.74892	0.00015	0.00330506
GPR179	chr17:36481492-36499693	0.929795	2.02938	1.12606	1.38335	0.0035	0.0336944

PALLD	chr4:169418216-169849608	21.0793	45.9952	1.12566	1.84219	0.00005	0.00136643
CSF3R	chr1:36931643-36948915	2.11385	4.60844	1.12441	1.40938	0.0027	0.0277348
QRICH2	chr17:74270129-74303761	1.89792	4.13447	1.12328	1.49649	0.001	0.0136643
RNASET2	chr6:167343003-167370077	15.7812	34.2703	1.11876	1.61261	0.0005	0.00814556
PRUNE2	chr9:79226291-79521003	20.5596	44.5908	1.11694	1.52778	0.0012	0.015451
PRRX1	chr1:170633312-170708541	6.80836	14.7345	1.11381	1.59131	0.00035	0.00620045
TGFB1	chr19:41836435-41859838	4.03187	8.7248	1.11367	1.40434	0.00255	0.026597
NUPR1	chr16:28548661-28550495	26.8457	58.0921	1.11365	1.61143	0.0006	0.00935947
TRIM47	chr17:73870244-73874656	9.27152	20.0562	1.11317	1.59237	0.00045	0.00751151
CHST6	chr16:75507021-75528926	7.89274	17.0629	1.11227	1.8312	0.00005	0.00136643
NOTCH3	chr19:15270443-15311792	5.04788	10.9088	1.11174	1.61632	0.0002	0.00407561
SHE	chr1:154451953-154474526	1.90027	4.10614	1.11157	1.46694	0.0017	0.0198318
ZBTB16	chr11:113930430-114121397	27.6421	59.6877	1.11057	1.70471	0.00005	0.00136643
ZNF652	chr17:47366567-47457456	11.8215	25.481	1.10801	1.73408	0.00025	0.00483727
SOX10	chr22:38368318-38380539	11.7169	25.2473	1.10754	1.65905	0.00025	0.00483727
CNTN2	chr1:205012339-205047171	38.2552	82.3763	1.10657	1.43494	0.003	0.0299609
MYO9B	chr19:17186590-17324104	8.63297	18.5767	1.10557	1.75164	0.0001	0.00238525
STAT5A	chr17:40439564-40463960	2.07748	4.46785	1.10475	1.40831	0.0035	0.0336944
COLGALT1	chr19:17666510-17693965	8.20303	17.6291	1.10373	1.75365	0.0001	0.00238525
SLC22A23	chr6:3269206-3456793	13.0177	27.9642	1.1031	1.38445	0.00185	0.0212581
RAPGEF3	chr12:48128452-48152889	7.56505	16.2504	1.10305	1.77931	0.0001	0.00238525
FOSL2	chr2:28607275-28637516	14.3656	30.828	1.10162	1.65186	0.00055	0.00876569
PHF19	chr9:123617928-123657174	6.59672	14.1456	1.10053	1.33398	0.0022	0.0239231
ATP11A	chr13:113344642-113541482	7.97281	17.0947	1.10039	1.80408	0.00005	0.00136643
BIRC3	chr11:102188180-102210135	1.48727	3.18732	1.09968	1.43715	0.0013	0.0163446
SH3TC2	chr5:148361712-148442737	2.84598	6.09196	1.09798	1.74908	0.00005	0.00136643
PXK	chr3:58318616-58411854	23.2207	49.682	1.09731	1.79118	0.00005	0.00136643
MYRF	chr11:61520120-61555989	17.043	36.457	1.09701	1.62805	0.00025	0.00483727
CYBB	chrX:37639269-37672714	6.69956	14.3296	1.09686	1.58147	0.00025	0.00483727
HIP1	chr7:75162618-75368290	9.41541	20.0767	1.09242	1.7722	0.00005	0.00136643
JMJD6	chr17:74708913-74722881	7.89294	16.826	1.09205	1.3899	0.0023	0.0246907
TIE1	chr1:43766565-43788781	2.77355	5.90437	1.09005	1.41707	0.00145	0.0177865
PTPRC	chr1:198608097-198726605	5.74628	12.2205	1.0886	1.32826	0.0006	0.00935947
CLDND1	chr3:98234316-98241910	136.944	290.22	1.08357	1.63731	0.0006	0.00935947
LIFR	chr5:38475064-38671318	41.6892	88.3269	1.08318	1.5232	0.00085	0.0120733
WASF2	chr1:27730733-27816678	15.8872	33.6593	1.08314	1.85449	0.0001	0.00238525
ACSM5	chr16:20420855-20452281	2.69469	5.69706	1.0801	1.22719	0.00585	0.0490104
IGDCC4	chr15:65673824-65715410	3.76173	7.93412	1.07667	1.60124	0.0005	0.00814556
GPRC5B	chr16:19870292-19896151	151.693	319.496	1.07465	1.52555	0.0007	0.010395
DNA2	chr10:70173820-70231878	1.91143	4.02347	1.07379	1.32697	0.0049	0.0430141
FMN1	chr15:33057744-33486934	3.93605	8.28444	1.07365	1.33426	0.00375	0.0355766
TMEM235	chr17:76227390-76237068	6.42383	13.4992	1.07137	1.34275	0.0029	0.0292774
CNTNAP3	chr9:39072763-39288300	1.81476	3.81093	1.07037	1.40101	0.0016	0.0190433
SLC44A1	chr9:108006893-108159628	37.1396	77.8979	1.06863	1.55367	0.00085	0.0120733
TRABD	chr22:50624359-50638027	3.2829	6.88516	1.06852	1.26795	0.00365	0.0347776
YBX3	chr12:10851675-10875953	9.54521	20.0052	1.06753	1.30187	0.00595	0.0496124
HSPB8	chr12:119616594-119632551	73.1083	153.104	1.0664	1.63581	0.00035	0.00620045
CCDC144CP	chr17:20224486-20305504	4.47142	9.35411	1.06487	1.61288	0.0002	0.00407561

SPSB1	chr1:9352940-9429590	3.58947	7.50851	1.06476	1.37963	0.00155	0.0185989
CLMN	chr14:95648275-95786245	9.48352	19.8362	1.06464	1.65372	0.00065	0.00986869
PML	chr15:74287013-74340155	4.11978	8.61287	1.06393	1.45022	0.00145	0.0177865
CDK18	chr1:205473683-205501921	15.875	33.1691	1.06309	1.54015	0.00065	0.00986869
COL12A1	chr6:75794041-75915623	1.17321	2.45058	1.06265	1.42683	0.0017	0.0198318
SLC7A5P2	chr16:21529229-21531765	5.26211	10.9882	1.06224	1.30019	0.006	0.0498407
CMKLR1	chr12:108681820-108733094	1.46876	3.06458	1.06109	1.37678	0.00345	0.0333771
APOL6	chr22:36044423-36064456	3.01306	6.28391	1.06043	1.65196	0.0002	0.00407561
SAMD4A	chr14:55034329-55260033	16.5398	34.4715	1.05946	1.72876	0.00015	0.00330506
ECE1	chr1:21543739-21672034	10.9023	22.6638	1.05575	1.72652	0.00025	0.00483727
ADAM28	chr8:24151579-24212726	3.30829	6.87427	1.05512	1.27416	0.00455	0.0407742
EFNA1	chr1:155100348-155107386	7.04107	14.6304	1.05511	1.45624	0.00145	0.0177865
HRH3	chr20:60790016-60795323	4.76739	9.89646	1.05371	1.29089	0.0049	0.0430141
DHX34	chr19:47852537-47885961	3.40559	7.04236	1.04815	1.45692	0.001	0.0136643
KIF1C	chr17:4901242-4931694	24.1924	49.9886	1.04705	1.56776	0.00065	0.00986869
STOM	chr9:124101265-124132582	52.1406	107.732	1.04697	1.60116	0.0004	0.00687883
LOC646214	chr15:21932513-21940739	1.96534	4.05978	1.04662	1.47505	0.00115	0.0149934
LRRC55	chr11:56949220-56959188	2.26323	4.67327	1.04605	1.40379	0.0022	0.0239231
FRYL	chr4:48499379-48782316	17.9731	37.086	1.04504	1.62389	0.00055	0.00876569
NDRG1	chr8:134249413-134309547	62.7204	129.265	1.04333	1.57049	0.00065	0.00986869
FAM222A	chr12:110152186-110211292	4.2459	8.74621	1.04259	1.35461	0.0026	0.0269589
TOB2	chr22:41829491-41843027	17.8502	36.7401	1.04141	1.75452	0.0002	0.00407561
RNF19A	chr8:101269286-101348446	30.3478	62.4157	1.04032	1.5923	0.00035	0.00620045
APBB1IP	chr10:26727265-26856732	3.82592	7.86857	1.04029	1.35186	0.00215	0.0235978
LOC10012991	chr4:773936-775636	7.05647	14.5043	1.03946	1.24997	0.0041	0.0378777
NPL	chr1:182758583-182799519	6.33208	13.0115	1.03904	1.39677	0.00285	0.028905
PCDH18	chr4:138440073-138453629	2.18146	4.4752	1.03666	1.41136	0.0012	0.015451
S1PR3	chr9:91605777-91620069	4.70544	9.64854	1.03598	1.36671	0.0046	0.0411177
CARD8	chr19:48711342-48761450	5.12872	10.5155	1.03585	1.04875	0.0056	0.0474801
HAUS5	chr19:36103645-36116251	1.47189	3.01512	1.03454	1.23441	0.0047	0.0418842
PAN2	chr12:56704212-56727837	7.01027	14.3581	1.03433	1.34331	0.003	0.0299609
CSF1R	chr5:149432853-149492935	14.1638	28.9814	1.03292	1.70316	0.0001	0.00238525
EMILIN1	chr2:27301434-27309265	2.90165	5.93162	1.03156	1.34159	0.0016	0.0190433
CLDN11	chr3:170136652-170152479	41.6544	85.1199	1.03103	1.63	0.00025	0.00483727
OTUD7B	chr1:149912228-149982686	13.5787	27.6889	1.02797	1.75	0.00005	0.00136643
PTPRT	chr20:40701391-41818557	5.28171	10.7559	1.02605	1.54099	0.0006	0.00935947
GFPT2	chr5:179727699-179780315	6.50279	13.2334	1.02506	1.53191	0.001	0.0136643
HEYL	chr1:40089102-40105348	1.52946	3.11211	1.02487	1.23517	0.00515	0.0446089
DDX60L	chr4:169277885-169401665	1.70873	3.47554	1.02431	1.31055	0.00445	0.0401639
CREB5	chr7:28338939-28865511	4.67923	9.50997	1.02317	1.64621	0.00025	0.00483727
MAOB	chrX:43625856-43741721	45.3068	92.0604	1.02285	1.55497	0.0005	0.00814556
MAPK4	chr18:48086483-48258196	18.8466	38.289	1.02263	1.6737	0.00025	0.00483727
ATP10A	chr15:25923859-26108349	2.76451	5.6105	1.02111	1.39941	0.00155	0.0185989
IL17RB	chr3:53880576-53899827	7.16347	14.5213	1.01944	1.44341	0.00195	0.0220762
CHORDC1	chr11:89933596-89956532	17.2676	34.997	1.01917	1.63807	0.0003	0.00551994
MOB3B	chr9:27325206-27529850	7.26622	14.688	1.01536	1.63181	0.0002	0.00407561
FRMD5	chr15:44162958-44487492	5.89642	11.8772	1.01028	1.55676	0.00055	0.00876569
BST2	chr19:17513747-17516457	10.1106	20.3347	1.00807	1.30335	0.002	0.0224405

CSF1	chr1:110453232-110473616	7.76047	15.5607	1.0037	1.50468	0.0015	0.0182223
BAHCC1	chr17:79373520-79433358	3.0054	6.02589	1.00362	1.43293	0.00145	0.0177865
ST18	chr8:53023391-53322439	9.51009	19.0407	1.00155	1.60747	0.00045	0.00751151
ESAM	chr11:124623018-124632223	5.86948	11.7306	0.998976	1.2903	0.003	0.0299609
CCDC88B	chr11:64107689-64125006	2.15168	4.29899	0.998536	1.27506	0.0041	0.0378777
MT1E	chr16:56659584-56661024	84.8828	169.386	0.996768	1.56174	0.00045	0.00751151
SNRNP48	chr6:7590431-7612200	3.69002	7.35031	0.994174	1.4027	0.00205	0.0228705
NACC2	chr9:138898382-138987131	33.1973	66.1026	0.99364	1.6161	0.0003	0.00551994
RNF213	chr17:78234659-78411884	9.85714	19.6145	0.992682	1.28695	0.0019	0.0216212
TJP2	chr9:71736179-71870124	31.3924	62.4649	0.99263	1.63164	0.0004	0.00687883
ITGAM	chr16:31271287-31344213	2.37823	4.71182	0.986395	1.283	0.0057	0.0480506
RHOBTB3	chr5:95066849-95132071	42.8283	84.6941	0.983696	1.48275	0.00095	0.0131856
FAM53B	chr10:126307862-126432930	13.8374	27.3251	0.981654	1.6267	0.0002	0.00407561
SRGAP1	chr12:64238540-64541613	3.41817	6.73884	0.979278	1.55722	0.0008	0.0115682
PARP10	chr8:145051319-145060635	4.33731	8.53528	0.976638	1.34427	0.0022	0.0239231
MOB3C	chr1:47073386-47082563	3.77527	7.427	0.976199	1.32093	0.0038	0.0358579
LRRC1	chr6:53659777-53788919	6.00802	11.8188	0.976127	1.40391	0.002	0.0224405
RASGRP3	chr2:33661415-33789798	6.55839	12.8985	0.975793	1.51084	0.0012	0.015451
DFNB31	chr9:117164359-117267736	6.31082	12.4015	0.974618	1.36607	0.00255	0.026597
TFEB	chr6:41651715-41703997	6.02336	11.8308	0.973907	1.35462	0.0037	0.035159
LDLRAD3	chr11:35965611-36252841	5.28449	10.3772	0.973582	1.34399	0.0028	0.0284799
ANKRD40	chr17:48770550-48785270	43.9581	86.2847	0.972976	1.57659	0.00055	0.00876569
KHNYN	chr14:24895739-24912111	6.35683	12.4736	0.972502	1.36135	0.00225	0.024287
GIMAP4	chr7:150264457-150271041	5.52955	10.8473	0.972102	1.3156	0.0028	0.0284799
ASPA	chr17:3377403-3402700	13.6446	26.7614	0.97183	1.41655	0.003	0.0299609
RELA	chr11:65421066-65430443	9.02717	17.6996	0.971372	1.37414	0.00225	0.024287
KCTD12	chr13:77454303-77460540	27.9145	54.722	0.971107	1.57844	0.0001	0.00238525
RALGDS	chr9:135973106-136024607	35.4204	69.2462	0.967152	1.50945	0.00165	0.0194937
ARAP2	chr4:36067619-36245979	27.8239	54.3526	0.966022	1.55888	0.0003	0.00551994
PREX1	chr20:47240792-47444420	31.0352	60.5799	0.964935	1.49686	0.0005	0.00814556
SYNGR2	chr17:76164670-76169009	19.3355	37.7383	0.964775	1.47521	0.00125	0.0158974
PLEKHH1	chr14:68000007-68067017	24.9469	48.68	0.964468	1.31782	0.0056	0.0474801
CENPJ	chr13:25456411-25497027	2.6261	5.1229	0.964039	1.34866	0.005	0.0436961
LINC00844	chr10:60759277-60761377	34.5826	67.372	0.9621	1.4289	0.00125	0.0158974
ARHGEF1	chr19:42387266-42411604	6.56126	12.7742	0.961182	1.40613	0.0016	0.0190433
SUN2	chr22:39130718-39152024	66.1509	128.6	0.959056	1.39714	0.00225	0.024287
ATF7	chr12:53901639-54020199	8.50911	16.5384	0.958743	1.09428	0.0013	0.0163446
LINC00639	chr14:39218542-39386086	4.35371	8.44868	0.956481	1.29801	0.00335	0.0326065
GNA12	chr7:2767740-2883959	29.4379	57.0718	0.955101	1.58213	0.00035	0.00620045
ZFHX3	chr16:72816785-73092534	3.2665	6.33081	0.954647	1.55802	0.0004	0.00687883
PAPLN	chr14:73704204-73741347	5.70677	11.0473	0.952946	1.46747	0.0009	0.0126208
STARD9	chr15:42867856-43013196	3.23424	6.25994	0.952719	1.51815	0.0006	0.00935947
LAMP2	chrX:119560002-119603204	92.0046	178.018	0.952246	1.3428	0.0033	0.0321732
ITPRIPL2	chr16:19125253-19132952	4.80353	9.28507	0.950816	1.49178	0.0009	0.0126208
GNB4	chr3:179113875-179169371	8.06175	15.5733	0.949907	1.5597	0.00045	0.00751151
SCARA3	chr8:27491576-27534286	18.9444	36.5883	0.949613	1.44297	0.0018	0.0207648
CDC25B	chr20:3767418-3786768	8.7024	16.7953	0.948569	1.44062	0.00405	0.0375536
PLXNA3	chrX:153686620-153701989	4.74118	9.12987	0.945348	1.47246	0.0012	0.015451

CD4	chr12:6898637-6929976	3.89205	7.49221	0.944863	1.31041	0.0036	0.0344315
LINC01105	chr2:6072818-6120350	8.7155	16.7747	0.944633	1.54064	0.00015	0.00330506
SCRIB	chr8:144873089-144897549	9.38286	18.0503	0.943923	1.49451	0.0008	0.0115682
VWA1	chr1:1370902-1378262	7.97958	15.3375	0.942679	1.45018	0.00095	0.0131856
MXI1	chr10:111967362-112047123	32.4972	62.3812	0.940797	1.58058	0.00045	0.00751151
PCSK6	chr15:101844132-102030187	10.7668	20.6497	0.939532	1.34026	0.00115	0.0149934
REL	chr2:61108629-61155291	3.13131	6.00035	0.938283	1.44619	0.00185	0.0212581
UGT8	chr4:115519610-115598202	31.1421	59.64	0.937417	1.51384	0.0011	0.0145132
ARHGAP29	chr1:94634462-94703307	4.7806	9.1546	0.937307	1.51146	0.0008	0.0115682
TNS1	chr2:218664511-218808796	19.6056	37.5011	0.935669	1.35093	0.00235	0.024984
KCNMB4	chr12:70760061-70828072	21.189	40.5276	0.93559	1.54019	0.00025	0.00483727
RXRA	chr9:137218308-137332432	18.8112	35.9713	0.93526	1.54655	0.00065	0.00986869
TRIM22	chr11:5710816-5732093	7.92115	15.1449	0.935048	1.36492	0.00305	0.0303571
ARHGAP42	chr11:100558406-100861656	3.67627	6.99124	0.927308	1.35767	0.0011	0.0145132
FMNL3	chr12:50017196-50101197	4.53842	8.63	0.927171	1.26239	0.00505	0.0440021
TTYH2	chr17:72209695-72258157	26.9612	51.2059	0.925426	1.42266	0.00155	0.0185989
STARD13	chr13:33677271-34250972	7.7095	14.6364	0.924848	1.46611	0.0015	0.0182223
CLK1	chr2:201717731-201729467	51.2821	97.3338	0.924485	1.51732	0.0011	0.0145132
PLEKHO2	chr15:65134081-65160201	8.71168	16.5165	0.922888	1.44899	0.0009	0.0126208
NYNRIN	chr14:24867991-24888494	2.26421	4.28773	0.921204	1.34397	0.0018	0.0207648
PXN	chr12:120639093-120703574	7.95739	15.0586	0.920221	1.37208	0.0046	0.0411178
ETV6	chr12:11802787-12079107	2.36675	4.47782	0.919887	1.28716	0.00435	0.0394838
CALD1	chr7:134464163-134655480	16.1095	30.4779	0.919849	1.495	0.0012	0.015451
RBM48	chr7:92158086-92166823	10.2758	19.4321	0.919197	1.33416	0.00265	0.0273487
MAP3K11	chr11:65365225-65381720	7.56304	14.2953	0.918506	1.36543	0.0028	0.0284799
NOTCH1	chr9:139388895-139440238	7.88443	14.9018	0.918405	1.4439	0.00155	0.0185989
EHBP1L1	chr11:65343508-65360116	3.95811	7.4786	0.917955	1.3836	0.00215	0.0235978
MAF	chr16:79627744-79634622	8.15817	15.4111	0.917652	1.397	0.00095	0.0131856
MGST1	chr12:16500075-16530123	47.518	89.5918	0.914893	1.28314	0.00415	0.0381398
C1orf198	chr1:230972864-231005335	38.9231	73.2342	0.91189	1.53415	0.00065	0.00986869
GREM1	chr15:33010204-33026870	5.57249	10.4769	0.910815	1.2911	0.0043	0.0391105
ARHGAP23	chr17:36584719-36668628	16.6466	31.1999	0.906317	1.4529	0.00075	0.0109258
CYP1B1	chr2:38294745-38303323	5.12241	9.59674	0.905721	1.26296	0.00305	0.0303571
ENGASE	chr17:77071018-77084685	5.66693	10.5971	0.90303	1.35436	0.00255	0.026597
CYP2J2	chr1:60358979-60392423	8.19729	15.312	0.901442	1.31873	0.00255	0.026597
SLC16A9	chr10:61410521-61469649	10.2419	19.1204	0.900627	1.38397	0.0019	0.0216212
C21orf91	chr21:19149720-19191703	9.56737	17.8591	0.900462	1.46515	0.00125	0.0158974
CARHSP1	chr16:8946798-8962869	9.72779	18.1353	0.898614	1.3711	0.0023	0.0246907
RPS6KA1	chr1:26856248-26901520	5.38104	10.0196	0.896875	1.26999	0.006	0.0498407
ABCA9	chr17:66970772-67057136	3.84202	7.14955	0.895988	1.32633	0.00415	0.0381398
EMX2OS	chr10:119243803-119309057	11.8982	22.1183	0.894502	1.27888	0.0052	0.0449757
LHFPL2	chr5:77781037-77944648	9.3139	17.3019	0.893476	1.40484	0.0013	0.0163446
DIP2A	chr21:47878861-47989926	9.4264	17.5039	0.892894	1.36429	0.0013	0.0163446
POLE	chr12:133200347-133264110	2.52491	4.67873	0.889882	1.30383	0.00235	0.024984
SNX33	chr15:75941347-75950968	5.91725	10.9612	0.889401	1.3149	0.0025	0.0262151
STK10	chr5:171469073-171615346	2.18603	4.04937	0.889386	1.25332	0.00515	0.0446089
RGCC	chr13:42031541-42045013	35.3974	65.5284	0.888476	1.42055	0.001	0.0136643
NFKB1	chr4:103422485-103538459	4.98068	9.22014	0.888445	1.34971	0.0025	0.0262151

NUP188	chr9:131707808-131769375	6.71811	12.4186	0.886375	1.3439	0.00365	0.0347776
AXL	chr19:41725103-41767672	10.091	18.636	0.885022	1.4599	0.00105	0.0140962
CCP110	chr16:19535178-19564728	25.3482	46.7647	0.883538	1.43151	0.00155	0.0185989
ARHGAP31	chr3:119013219-119138323	6.71814	12.3914	0.883207	1.48071	0.0007	0.010395
KIAA1958	chr9:115249247-115427591	5.13191	9.46059	0.882434	1.42891	0.0016	0.0190433
SAMD9L	chr7:92759367-92777680	2.42304	4.46493	0.881819	1.29193	0.0031	0.0306643
CNTFR	chr9:34551429-34590138	12.7795	23.5423	0.88142	1.34754	0.0028	0.0284799
KLF6	chr10:3818187-3827473	9.37046	17.2493	0.880345	1.41987	0.001	0.0136643
PLA2G16	chr11:63341943-63381941	56.016	103.004	0.878783	1.4634	0.00145	0.0177865
ERBB3	chr12:56473808-56497291	11.539	21.2073	0.878044	1.15513	0.00155	0.0185989
GNA13	chr17:63005406-63052920	23.7486	43.6042	0.876623	1.48505	0.00135	0.0168292
RYR1	chr19:38924339-39078204	4.56038	8.36961	0.876008	1.43826	0.00165	0.0194937
MTUS1	chr8:17501302-17658426	28.2237	51.7505	0.874666	1.32428	0.0043	0.0391105
CPOX	chr3:98298289-98312455	11.3133	20.7406	0.874442	1.33955	0.00235	0.024984
KLK6	chr19:51461886-51472929	13.4811	24.7139	0.874382	1.25785	0.00475	0.0422443
PTTG1IP	chr21:46269499-46293818	57.5729	105.511	0.873931	1.39367	0.00255	0.026597
GOLIM4	chr3:167727653-167813417	24.2341	44.3973	0.87343	1.43739	0.00165	0.0194937
PODXL	chr7:131185020-131241376	13.5922	24.8843	0.872455	1.41716	0.0019	0.0216212
AEBP1	chr7:44143959-44154164	18.5701	33.9448	0.870207	1.4721	0.001	0.0136643
PLD1	chr3:171318194-171528284	5.82377	10.6441	0.870032	1.37807	0.00275	0.0281336
PLXNB1	chr3:48445260-48471460	33.5777	61.1915	0.865826	1.30728	0.004	0.0371878
INF2	chr14:105155942-105185947	16.5055	30.0686	0.865314	1.29634	0.0026	0.0269589
TMEM165	chr4:56262079-56292342	26.0729	47.463	0.864255	1.37066	0.0024	0.0253778
CD109	chr6:74403625-74538041	3.05208	5.55537	0.864093	1.23744	0.006	0.0498407
RGMA	chr15:93586635-93632443	19.3669	35.2443	0.863794	1.2994	0.00365	0.0347776
IL13RA1	chrX:117861558-117928496	9.2211	16.7798	0.863713	1.34113	0.0013	0.0163446
EEF2K	chr16:22217591-22300066	5.39619	9.81108	0.862471	1.42083	0.00135	0.0168292
SP1	chr12:53773978-53810226	9.51436	17.2972	0.862358	1.46426	0.0013	0.0163446
CPQ	chr8:97657454-98155731	13.927	25.2236	0.856892	1.34782	0.0034	0.0330021
FRMD4B	chr3:69217933-69435455	6.11632	11.0751	0.856585	1.37166	0.0024	0.0253778
PARP14	chr3:122399671-122449687	7.05153	12.7606	0.855684	1.42637	0.00125	0.0158974
HMBOX1	chr8:28747910-28910242	15.7955	28.5518	0.854065	1.44091	0.0017	0.0198318
TMTC2	chr12:83080933-83528067	13.9965	25.2711	0.85242	1.41786	0.0013	0.0163446
KIAA0930	chr22:45588122-45636650	33.0036	59.4214	0.848359	1.31075	0.0037	0.035159
SMOX	chr20:4129425-4168394	22.0225	39.6481	0.848274	1.35986	0.00225	0.024287
RAB30	chr11:82684174-82782965	13.1702	23.7018	0.84772	1.37378	0.0029	0.0292774
BHLHE41	chr12:26272958-26278003	18.0933	32.4805	0.844119	1.39768	0.0014	0.0173787
OMA1	chr1:58946390-59012446	11.151	20.0171	0.844062	1.27205	0.005	0.0436961
ACACB	chr12:109577201-109706030	8.00948	14.3625	0.842526	1.35858	0.0026	0.0269589
SLC7A5	chr16:87863628-87903100	21.5058	38.5554	0.842206	1.36221	0.0015	0.0182223
SWAP70	chr11:9685627-9774507	9.96066	17.8519	0.841764	1.41142	0.00125	0.0158974
SLC31A2	chr9:115913237-115926422	16.8926	30.2674	0.84138	1.30827	0.0039	0.0365472
DEPTOR	chr8:120885894-121063157	9.20365	16.4617	0.838836	1.27004	0.00375	0.0355766
LPAR1	chr9:113636053-113800365	52.7572	94.2933	0.837786	1.34649	0.003	0.0299609
PRRG1	chrX:37208527-37316548	11.822	21.1136	0.836696	1.39916	0.00155	0.0185989
RNPEPL1	chr2:241508003-241518149	8.26128	14.7373	0.83504	1.2435	0.00555	0.0473065
ZNF395	chr8:28203101-28243977	10.1102	18.0107	0.833039	1.37603	0.0013	0.0163446
SH3BP2	chr4:2794749-2842823	4.90999	8.7427	0.832357	1.3399	0.0041	0.0378777

WWTR1	chr3:149235021-149421060	15.321	27.2333	0.829857	1.30381	0.003	0.0299609
FOXN2	chr2:48541794-48606434	11.8271	20.9889	0.827527	1.41152	0.00155	0.0185989
PPP1R3E	chr14:23765129-23772057	5.77358	10.2299	0.825258	1.25983	0.00285	0.028905
CLIC4	chr1:25071759-25170815	47.3442	83.8816	0.825168	1.34627	0.0024	0.0253778
AGRN	chr1:955502-991499	6.94587	12.299	0.824316	1.28295	0.00355	0.0340827
SYNM	chr15:99645285-99675800	32.6525	57.6996	0.821368	1.24613	0.00505	0.0440021
ST6GAL1	chr3:186648314-186796341	14.7463	26.0505	0.820956	1.32684	0.00315	0.0310023
MAP4K5	chr14:50885210-50999376	17.1209	30.1492	0.816364	1.38966	0.00215	0.0235978
RNF130	chr5:179382066-179499118	35.4679	62.3673	0.814275	1.37823	0.0015	0.0182223
FYCO1	chr3:45959390-46037316	5.09268	8.9497	0.813414	1.34806	0.0021	0.0232225
PADI2	chr1:17393255-17445948	42.4649	74.5812	0.812542	1.29813	0.00245	0.0257982
ZCCHC24	chr10:81142082-81205383	39.3427	68.9012	0.808432	1.31111	0.00295	0.0296802
PALD1	chr10:72238563-72328206	7.06713	12.3767	0.808427	1.2913	0.0029	0.0292774
FAM167A	chr8:11197145-11324276	11.5996	20.2938	0.806969	1.23495	0.0056	0.0474801
P4HA1	chr10:74766979-74856732	14.2481	24.9228	0.806701	1.25963	0.00565	0.0476976
UTRN	chr6:144612872-145174170	12.7774	22.3435	0.80626	1.30173	0.00165	0.0194937
ADCY7	chr16:50300450-50352045	4.51984	7.90249	0.806036	1.2298	0.0046	0.0411178
SEMA6A	chr5:115779250-115910551	8.47995	14.8079	0.804234	1.28054	0.00425	0.0387958
FAM20C	chr7:192968-300740	10.5128	18.3012	0.799795	1.24591	0.00385	0.0362521
CRTAP	chr3:33155449-33189265	16.5789	28.8473	0.799088	1.32677	0.0028	0.0284799
ATG4C	chr1:63249776-63330941	12.0943	20.9964	0.795817	1.28335	0.0044	0.039835
CBFB	chr16:67063049-67134958	14.9598	25.9161	0.792757	1.26179	0.00485	0.0427669
GRAMD3	chr5:125695787-125829853	34.4292	59.5082	0.789456	1.29145	0.00545	0.0466799
ATN1	chr12:7033625-7051484	19.9122	34.3796	0.787899	1.23293	0.0058	0.048777
CSPG4	chr15:75966662-76005189	5.66124	9.77172	0.787495	1.25515	0.00365	0.0347776
ZNF621	chr3:40566368-40581285	6.53988	11.2816	0.786634	1.30089	0.004	0.0371878
VEZF1	chr17:56048909-56065615	12.8517	22.1452	0.78503	1.32472	0.0022	0.0239231
PFKFB3	chr10:6186842-6277507	41.6257	71.7213	0.784926	1.2349	0.00465	0.0415015
DIP2B	chr12:50898767-51142450	24.2759	41.6992	0.780493	1.25999	0.0047	0.0418842
FNBP1	chr9:132649465-132805473	16.1871	27.7713	0.778746	1.31684	0.0028	0.0284799
CTDSP2	chr12:58213709-58240747	32.2927	55.3687	0.77786	1.28188	0.00505	0.0440021
PER1	chr17:8043787-8055753	11.8294	20.1827	0.770743	1.23361	0.00535	0.0460471
PHLPP1	chr18:60382671-60647676	27.413	46.7111	0.768908	1.2545	0.00395	0.0368006
LINC01000	chr7:128281294-128301052	8.10304	13.7426	0.762124	1.21052	0.00495	0.0433451
KIAA1551	chr12:32112352-32146043	8.73418	14.7967	0.760529	1.31193	0.0028	0.0284799
MEGF10	chr5:126626455-126796910	17.03	28.8307	0.759525	1.26662	0.0045	0.0404495
MAML1	chr5:179159850-179204287	6.99033	11.8217	0.758009	1.21498	0.0047	0.0418842
HIP1R	chr12:123320038-123347507	25.6154	43.229	0.754987	1.20784	0.0055	0.0470395
FOXN3	chr14:89622515-90085494	11.4901	19.3901	0.754933	1.27169	0.00455	0.0407742
GLTP	chr12:110288747-110318293	19.6856	33.2141	0.75466	1.24003	0.00405	0.0375536
C2CD2	chr21:43305218-43373999	8.88793	14.9575	0.750954	1.24585	0.0059	0.0492421
SPTLC2	chr14:77972339-78083110	17.4759	29.3863	0.74978	1.23985	0.0048	0.0425388
DENND4B	chr1:153901976-153919154	11.3567	19.0134	0.74347	1.21447	0.00485	0.0427669
CDH20	chr18:59157774-59222365	15.9763	26.7129	0.741602	1.22469	0.00565	0.0476976
ROCK1	chr18:18529702-18691812	11.6524	19.4654	0.74029	1.27295	0.00405	0.0375536
TSC22D4	chr7:100064141-100076902	33.6335	56.1409	0.739151	1.24119	0.0059	0.0492421
CAPN2	chr1:223889294-223963720	28.5244	47.5329	0.736729	1.23448	0.00565	0.0476976
ZMIZ1	chr10:80828791-81076285	15.2024	25.1611	0.726893	1.18454	0.0059	0.0492421

RALGAPA2	chr20:20370271-20693266	6.2638	10.3379	0.722839	1.21722	0.00455	0.0407742
CREBRF	chr5:172483354-172566291	15.1012	24.8463	0.718367	1.18102	0.0056	0.0474801
IVNS1ABP	chr1:185265521-185286461	19.2619	31.5732	0.712947	1.2267	0.00395	0.0368006
CYCS	chr7:25158269-25164980	31.125	18.996	-0.712379	-1.20839	0.0057	0.0480506
PRKAA2	chr1:57110989-57181008	10.3946	6.25697	-0.732304	-1.19078	0.00545	0.0466799
NCAN	chr19:19322781-19363061	37.836	22.7486	-0.73398	-1.19842	0.0039	0.0365472
UBE2N	chr12:93802087-93836026	41.195	24.7677	-0.734008	-1.201	0.0052	0.0449757
IARS	chr9:94972489-95056038	32.8889	19.7332	-0.736974	-1.22531	0.0056	0.0474801
KLHDC3	chr6:42981840-42989036	75.384	45.2211	-0.737262	-1.26505	0.00385	0.0362521
EHD3	chr2:31456879-31491260	39.7597	23.8447	-0.737637	-1.22414	0.00485	0.0427669
SLC30A9	chr4:41992522-42089551	45.6774	27.3122	-0.741936	-1.24535	0.00525	0.0453636
GPCPD1	chr20:5525079-5591672	11.5924	6.93078	-0.74209	-1.19375	0.006	0.0498407
KIAA0368	chr9:114122972-114247025	47.7456	28.5164	-0.743577	-1.20447	0.00565	0.0476976
PRICKLE2	chr3:64053639-64211131	36.417	21.7241	-0.745313	-1.21012	0.00535	0.0460471
SCN2B	chr11:118033518-118047337	30.3069	18.0395	-0.748481	-1.23598	0.00435	0.0394838
PITPNA	chr17:1420212-1466110	39.222	23.3422	-0.748723	-1.23799	0.00515	0.0446089
WDR7	chr18:54318615-54697036	40.0476	23.7998	-0.750768	-1.22767	0.0056	0.0474801
HIST1H4E	chr6:26204872-26205249	442.547	262.613	-0.752893	-1.26446	0.0043	0.0391105
AREL1	chr14:75127954-75179807	16.7976	9.94335	-0.756454	-1.28262	0.00425	0.0387958
FARSA	chr19:13033283-13044558	28.1188	16.6361	-0.757218	-1.21713	0.0049	0.0430141
YPEL5	chr2:30369749-30383399	71.8692	42.4834	-0.758475	-1.23294	0.00545	0.0466799
WDR17	chr4:176986984-177103979	20.7918	12.2785	-0.759877	-1.26903	0.00445	0.0401639
SMAP2	chr1:40839377-40888998	42.1317	24.8254	-0.763085	-1.29485	0.005	0.0436961
DDX41	chr5:176938577-176943967	24.839	14.6147	-0.765187	-1.25002	0.00555	0.0473065
ATP13A2	chr1:17312452-17338467	46.6878	27.4417	-0.766676	-1.28975	0.00415	0.0381398
SHISA7	chr19:55940104-55954230	19.6944	11.5621	-0.76838	-1.22218	0.00395	0.0368006
DDX24	chr14:94517267-94547558	89.7222	52.6558	-0.768874	-1.25353	0.00425	0.0387958
SDHA	chr5:218355-256814	58.9182	34.5594	-0.769636	-1.29501	0.00265	0.0273487
LRRN2	chr1:204586302-204654597	25.3662	14.8742	-0.770098	-1.28306	0.0039	0.0365472
COX8A	chr11:63742078-63744015	195.844	114.578	-0.773377	-1.25976	0.00395	0.0368006
BASP1	chr5:17130136-17276954	112.644	65.8815	-0.773827	-1.25556	0.00485	0.0427669
DYRK2	chr12:68042511-68056444	12.0004	7.0181	-0.773928	-1.24407	0.00555	0.0473065
PSMC3	chr11:47440319-47448024	48.9837	28.6435	-0.774093	-1.30312	0.0019	0.0216212
VAMP2	chr17:8062464-8066293	145.518	85.0461	-0.774879	-1.28135	0.0029	0.0292774
ATP6AP1	chrX:153656977-153664863	77.2103	45.1056	-0.775487	-1.31435	0.0033	0.0321732
TSPYL2	chrX:53111541-53117728	73.633	42.9587	-0.777402	-1.29251	0.0042	0.0384589
P4HTM	chr3:49027340-49044581	38.7877	22.6195	-0.778033	-1.2934	0.00415	0.0381398
SCAMP5	chr15:75287875-75313836	87.5817	51.062	-0.77838	-1.27934	0.0031	0.0306643
ZDBF2	chr2:207139364-207179150	18.7366	10.8909	-0.782736	-1.30468	0.00335	0.0326065
PRDX3	chr10:120927214-120938377	68.9965	40.0991	-0.782951	-1.21874	0.0056	0.0474801
COX6B1	chr19:36139124-36149686	166.344	96.5568	-0.784724	-1.31665	0.00215	0.0235978
TMEM9	chr1:201103898-201140710	51.5566	29.9169	-0.785194	-1.32496	0.00505	0.0440021
HIGD1A	chr3:42824399-42846027	68.1339	39.535	-0.785243	-1.2376	0.00515	0.0446089
NDUFS1	chr2:206987802-207024243	60.734	35.2355	-0.785475	-1.28421	0.0038	0.0358579
SSTR2	chr17:71161159-71168062	15.6802	9.09508	-0.785788	-1.22064	0.00485	0.0427669
GPRIN1	chr5:176022802-176037131	28.2053	16.3533	-0.786379	-1.25277	0.00525	0.0453636
COX5B	chr2:98262520-98264657	272.343	157.83	-0.787052	-1.32819	0.00305	0.0303571
DDX1	chr2:15731744-15771235	36.9723	21.3646	-0.791222	-1.33355	0.00235	0.024984

NEDD8	chr14:24683142-24701576	109.324	63.1429	-0.791921	-1.21326	0.00565	0.0476976
PGAP1	chr2:197697727-197791454	28.0172	16.1641	-0.79352	-1.2421	0.0031	0.0306643
CSRNP3	chr2:166326156-166545917	29.1992	16.8361	-0.794369	-1.26841	0.00455	0.0407742
PIN1	chr19:9945882-9960365	76.506	44.0828	-0.795357	-1.27635	0.0039	0.0365472
CACNG4	chr17:64960979-65029518	12.0549	6.94072	-0.796463	-1.21325	0.00535	0.0460471
OCRL	chrX:128674251-128726530	24.8627	14.3	-0.797966	-1.30967	0.0041	0.0378777
REPS2	chrX:16964813-17171403	27.963	16.0825	-0.798029	-1.30394	0.00315	0.0310023
SCOC	chr4:141178439-141303710	97.6804	56.1708	-0.798249	-1.27246	0.0048	0.0425388
TMEM246	chr9:104237607-104249475	38.3053	22.0258	-0.798349	-1.29142	0.0034	0.0330021
NDUFB9	chr8:125551342-125562227	111.405	64.0585	-0.798355	-1.29347	0.00455	0.0407742
IMMT	chr2:86371054-86422893	24.174	13.8878	-0.799641	-1.29456	0.0046	0.0411178
RTF1	chr15:41709301-41775761	16.3321	9.38245	-0.799675	-1.34737	0.0021	0.0232225
MAPK8IP2	chr22:51039113-51049979	67.6494	38.8387	-0.800581	-1.33726	0.00255	0.026597
NDUFB8	chr10:102283485-102289680	154.639	88.7148	-0.801661	-1.35164	0.0031	0.0306643
USP5	chr12:6961284-6975795	43.5984	25.0118	-0.801668	-1.35331	0.0022	0.0239231
MAP3K9	chr14:71189242-71275888	14.4688	8.29029	-0.803448	-1.31722	0.0036	0.0344315
SCAI	chr9:127704887-127905838	12.502	7.15809	-0.804513	-1.34013	0.0022	0.0239231
STMN3	chr20:62271057-62284963	140.33	80.3341	-0.804736	-1.29046	0.0034	0.0330021
KIAA1324	chr1:109656584-109749403	10.4574	5.97884	-0.806592	-1.26952	0.0054	0.0463417
BEX4	chrX:102470019-102472128	134.822	76.9899	-0.808312	-1.34271	0.0027	0.0277348
FAM81A	chr15:59730371-59815751	12.618	7.19719	-0.809975	-1.22002	0.00565	0.0476976
PNMAL1	chr19:46969747-46974820	60.731	34.5827	-0.812382	-1.28997	0.0046	0.0411178
GOLGA8B	chr15:34817483-34875771	13.0216	7.41402	-0.812581	-1.25916	0.0055	0.0470395
NCKAP1	chr2:183789578-183903586	118.322	67.3549	-0.812861	-1.23422	0.00535	0.0460471
COX6A1	chr12:120875892-120878545	256.026	145.403	-0.816233	-1.37582	0.00255	0.026597
LINGO1	chr15:77905368-77924709	43.9063	24.8677	-0.820158	-1.3444	0.00205	0.0228705
ATP6V1F	chr7:128502856-128505903	105.566	59.7215	-0.821825	-1.32949	0.0026	0.0269589
PIP4K2C	chr12:57984941-57997211	23.109	13.0598	-0.823323	-1.33802	0.0027	0.0277348
KCTD17	chr22:37447775-37459430	36.671	20.7228	-0.823423	-1.28644	0.0041	0.0378777
NDUFA9	chr12:4758263-4796720	31.5085	17.8037	-0.823565	-1.26341	0.0041	0.0378777
HAGH	chr16:1859103-1877195	50.241	28.381	-0.823939	-1.33017	0.0035	0.0336944
NDUFV1	chr11:67374322-67380012	81.3048	45.8813	-0.825432	-1.40779	0.00165	0.0194937
SHANK2	chr11:70313960-70935842	22.2434	12.5477	-0.825959	-1.27479	0.00485	0.0427669
LGMN	chr14:93170151-93215047	18.4706	10.4167	-0.826328	-1.22653	0.00585	0.0490104
INPP5F	chr10:121485558-121588662	50.8915	28.6933	-0.826709	-1.25522	0.00425	0.0387958
ADAM22	chr7:87563565-87832204	27.7757	15.6598	-0.826755	-1.34113	0.0016	0.0190433
ZBTB18	chr1:244212240-244220780	83.9567	47.3188	-0.827233	-1.35005	0.00275	0.0281336
GARS	chr7:30634180-30673648	32.1692	18.1303	-0.827274	-1.34849	0.0023	0.0246907
LMBRD2	chr5:36103413-36152015	40.6774	22.8978	-0.82902	-1.38345	0.00175	0.0203076
NDFIP1	chr5:141488323-141534008	107.225	60.356	-0.829068	-1.30395	0.00215	0.0235978
CNTN3	chr3:74311721-74570343	17.641	9.92982	-0.829092	-1.32951	0.0021	0.0232225
G3BP2	chr4:76567952-76598667	76.8005	43.2186	-0.829465	-1.28758	0.0044	0.039835
ENTPD6	chr20:25165307-25207360	61.2223	34.4084	-0.831295	-1.3728	0.00225	0.024287
MYH10	chr17:8377522-8534079	35.4782	19.9345	-0.831663	-1.33546	0.0027	0.0277348
ASNS	chr7:97481428-97501854	34.4698	19.3417	-0.83362	-1.36267	0.00355	0.0340827
SEPW1	chr19:48281841-48287943	444.418	249.243	-0.834364	-1.34189	0.00315	0.0310023
DYNLT3	chrX:37698088-37706889	34.3495	19.2543	-0.835107	-1.28788	0.0027	0.0277348
LSM4	chr19:18417039-18434001	25.8961	14.5145	-0.835237	-1.27793	0.00445	0.0401639

ZFPM2	chr8:106331146-106816767	14.3636	8.04781	-0.835754	-1.34128	0.0018	0.0207648
CDS1	chr4:85504056-85572493	12.8432	7.19512	-0.83592	-1.28309	0.00325	0.0317912
COX7C	chr5:85913783-85916583	474.815	265.92	-0.836374	-1.37743	0.00175	0.0203076
GRIN1	chr9:140033608-140064491	87.4203	48.8995	-0.838149	-1.30937	0.0039	0.0365472
RAB6B	chr3:133543079-133614691	99.2306	55.4587	-0.839372	-1.2545	0.0044	0.039835
PPARGC1A	chr4:23793643-23891700	7.68564	4.29266	-0.840294	-1.27083	0.00485	0.0427669
CEP41	chr7:130033611-130081051	6.24355	3.48674	-0.84049	-1.22069	0.0059	0.0492421
DIAPH2	chrX:95939661-96855597	11.8394	6.61078	-0.8407	-1.28738	0.00405	0.0375536
PLEKHB2	chr2:131862419-131907425	73.0392	40.7714	-0.841114	-1.38482	0.00295	0.0296802
CSPG5	chr3:47603727-47621730	37.8224	21.1103	-0.841292	-1.34876	0.0024	0.0253778
TRUB1	chr10:116697951-116737439	16.5476	9.23407	-0.841582	-1.27916	0.0036	0.0344315
CA11	chr19:49141271-49185502	96.7973	53.9899	-0.842278	-1.387	0.002	0.0224405
AVL9	chr7:32535175-32758780	21.2108	11.8276	-0.842642	-1.25937	0.00475	0.0422443
RIMBP2	chr12:130880680-131002410	17.8384	9.94675	-0.842685	-1.36079	0.0021	0.0232225
NDUFS5	chr1:39491966-39500308	196.594	109.601	-0.842955	-1.38254	0.00255	0.026597
UG0898H09	chr8:63161500-63903628	31.3995	17.4816	-0.84491	-1.2066	0.0049	0.0430141
ATP6V0C	chr16:2563726-2570224	118.729	66.0765	-0.845457	-1.37764	0.0019	0.0216212
KIFAP3	chr1:169890469-170043879	62.5524	34.8059	-0.845735	-1.41512	0.0015	0.0182223
PCDH7	chr4:30722029-31148423	31.1677	17.324	-0.84728	-1.29355	0.0049	0.0430141
WARS	chr14:100800124-100842680	37.0665	20.587	-0.848382	-1.38264	0.0032	0.0313892
SLC9A7	chrX:46458685-46618607	15.574	8.64508	-0.849193	-1.36653	0.00215	0.0235978
GRM1	chr6:146348917-146758734	10.7647	5.97542	-0.849201	-1.25484	0.0057	0.0480506
CADM3	chr1:159141376-159172932	120.94	67.1199	-0.849475	-1.31885	0.00265	0.0273487
ARHGEF28	chr5:72921982-73237818	5.07656	2.81713	-0.849628	-1.25468	0.00585	0.0490104
SLC25A4	chr4:186064416-186071538	31.8792	17.6807	-0.850442	-1.43444	0.001	0.0136643
WDR47	chr1:109512837-109584850	44.0286	24.4122	-0.850837	-1.39068	0.00155	0.0185989
NLGN4X	chrX:5808066-6146923	15.0321	8.32986	-0.851684	-1.40971	0.00195	0.0220762
PNMAL2	chr19:46984044-47104457	39.3542	21.794	-0.852585	-1.4268	0.00125	0.0158974
PTPRG	chr3:61547242-62304622	29.3209	16.2169	-0.85443	-1.39906	0.002	0.0224405
PODXL2	chr3:127348001-127391653	36.5983	20.2346	-0.85495	-1.43142	0.00175	0.0203076
VSNL1	chr2:17721806-17837706	163.061	90.1101	-0.855654	-1.2267	0.00475	0.0422443
ACSL4	chrX:108884563-108976621	30.1136	16.6318	-0.856466	-1.40048	0.00115	0.0149934
SLC25A23	chr19:6440074-6459781	67.1735	37.0809	-0.857215	-1.30663	0.00405	0.0375536
HSPA12A	chr10:118430702-118502085	55.1227	30.4192	-0.857663	-1.36253	0.00185	0.0212581
CETN2	chrX:151995870-151999301	48.2241	26.6008	-0.858283	-1.30112	0.0042	0.0384589
FAM126A	chr7:22980877-23053770	19.9848	11.0176	-0.859092	-1.43641	0.0015	0.0182223
COX7A2L	chr2:42577644-42588356	83.4903	46.025	-0.859189	-1.38052	0.002	0.0224405
OCIAD1	chr4:48833059-48863834	80.5017	44.3575	-0.85984	-1.43795	0.0019	0.0216212
NOS1	chr12:117645946-117799607	7.16381	3.9464	-0.860192	-1.34709	0.0028	0.0284799
SYNGR1	chr22:39745953-39781593	124.275	68.4561	-0.86028	-1.27708	0.0026	0.0269589
FAM115A	chr7:143548460-143599278	13.0831	7.2055	-0.860532	-1.45207	0.00135	0.0168292
USP11	chrX:47092313-47107727	104.395	57.4709	-0.861149	-1.378	0.00205	0.0228705
FRMPD4	chrX:12156584-12742642	14.2039	7.81808	-0.861402	-1.41539	0.0012	0.015451
SNCB	chr5:176047209-176057557	82.1627	45.1541	-0.863625	-1.38473	0.0023	0.0246907
OLA1	chr2:174937174-175113365	17.6739	9.6792	-0.868662	-1.38143	0.00205	0.0228705
SCN4B	chr11:118004091-118023630	18.2746	10.0028	-0.869434	-1.40196	0.0025	0.0262151
DLGAP1	chr18:3496029-4455266	53.2962	29.1632	-0.869884	-1.33812	0.0045	0.0404495
FIBP	chr11:65647283-65656010	26.7301	14.6207	-0.870459	-1.30441	0.0038	0.0358579

PLCH2	chr1:2407753-2436964	11.1482	6.08834	-0.872691	-1.25436	0.0026	0.0269589
SEH1L	chr18:12947982-12987536	21.2802	11.6134	-0.873722	-1.31056	0.00305	0.0303571
ARHGAP10	chr4:148653452-148993927	8.72629	4.76052	-0.874249	-1.25076	0.0038	0.0358579
AFG3L2	chr18:12328942-12377275	25.2289	13.743	-0.876378	-1.44872	0.00105	0.0140962
ADAM23	chr2:207308367-207485854	16.1183	8.76748	-0.878465	-1.44536	0.0007	0.010395
WDR61	chr15:78575577-78591940	22.639	12.3129	-0.878636	-1.21898	0.00445	0.0401639
NDRG3	chr20:35280168-35374541	52.2118	28.3964	-0.878668	-1.45334	0.0011	0.0145132
ACP1	chr2:264868-278282	48.2571	26.2213	-0.879999	-1.30743	0.0039	0.0365472
AP2S1	chr19:47341422-47354203	58.8696	31.9842	-0.880164	-1.34713	0.00275	0.0281336
SIPA1L3	chr19:38397860-38699012	13.7777	7.47873	-0.881466	-1.2419	0.00495	0.0433451
STS	chrX:7137471-7272682	7.98117	4.33126	-0.881813	-1.39474	0.00165	0.0194937
MKL2	chr16:14165195-14360630	56.8607	30.8485	-0.882228	-1.32477	0.0031	0.0306643
LYNX1	chr8:143845755-143859640	84.9019	46.0191	-0.883564	-1.34534	0.00235	0.024984
CX3CL1	chr16:57406413-57418956	42.8674	23.2274	-0.884051	-1.45918	0.0009	0.0126208
ARHGEF3	chr3:56761445-57113336	21.986	11.911	-0.884296	-1.44032	0.0019	0.0216212
AMPH	chr7:38423296-38671167	34.5651	18.6989	-0.886363	-1.39753	0.00135	0.0168292
BAI2	chr1:32192717-32229648	62.9838	34.0564	-0.887054	-1.36059	0.00175	0.0203076
KATNB1	chr16:57769659-57791162	16.3567	8.84419	-0.887078	-1.39527	0.0019	0.0216212
C5orf22	chr5:31532372-31555165	10.5768	5.71892	-0.887084	-1.31076	0.0028	0.0284799
RIMKLA	chr1:42846467-42889900	5.86153	3.16778	-0.887805	-1.41256	0.0017	0.0198318
UBL5	chr19:9938555-9940797	145.676	78.7211	-0.887942	-1.35263	0.0038	0.0358579
GABRA4	chr4:46920916-46996424	14.7038	7.94569	-0.887947	-1.38082	0.0021	0.0232225
OPALIN	chr10:98102974-98119122	41.5199	22.4351	-0.888045	-1.36329	0.0031	0.0306643
CCDC104	chr2:55746730-55772216	41.679	22.5184	-0.888217	-1.3932	0.00305	0.0303571
MXRA7	chr17:74671808-74707056	62.9224	33.9944	-0.888278	-1.28242	0.00445	0.0401639
C2CD2L	chr11:118978059-118987834	23.723	12.8117	-0.888831	-1.39992	0.0018	0.0207648
FZD1	chr7:90893782-90898132	9.47665	5.11755	-0.888922	-1.25175	0.0039	0.0365472
NDUFA8	chr9:124906337-124922098	55.8441	30.1521	-0.889146	-1.37875	0.00105	0.0140962
HTR1A	chr5:63255874-63258119	18.1465	9.79606	-0.889415	-1.20548	0.0059	0.0492421
TTC19	chr17:15879874-15932723	22.6319	12.2173	-0.889432	-1.28875	0.0048	0.0425388
CASD1	chr7:94139169-94186328	38.9211	21.004	-0.889893	-1.50108	0.00075	0.0109258
RAP2B	chr3:152880000-152888413	14.6525	7.90292	-0.890691	-1.48292	0.0004	0.00687883
PPIA	chr7:44836240-44842716	104.312	56.1913	-0.892488	-1.47072	0.0011	0.0145132
PCNXL2	chr1:233119881-233431459	25.348	13.6539	-0.892559	-1.48314	0.00055	0.00876569
JAKMIP1	chr4:6027925-6202318	34.0679	18.3471	-0.892863	-1.48337	0.00135	0.0168292
SLC25A11	chr17:4840425-4843462	45.0934	24.2778	-0.893279	-1.46598	0.0013	0.0163446
MRPL28	chr16:417383-420569	39.3023	21.1516	-0.893847	-1.38499	0.0019	0.0216212
KCNF1	chr2:11052062-11054351	17.7969	9.56386	-0.895964	-1.32336	0.0024	0.0253778
SH3GL2	chr9:17578951-17797126	75.6143	40.5875	-0.897625	-1.41225	0.00165	0.0194937
NRSN2	chr20:327369-335512	31.476	16.8941	-0.89773	-1.4685	0.00135	0.0168292
NAA20	chr20:19997933-20014273	35.4149	19.0044	-0.898022	-1.23453	0.00465	0.0415015
SLC22A17	chr14:23815519-23822121	207.069	111.103	-0.898216	-1.41893	0.0016	0.0190433
TTLL1	chr22:43435522-43485434	10.7132	5.74684	-0.898551	-1.19382	0.006	0.0498407
TMEM178B	chr7:140774031-141180179	21.4914	11.5254	-0.898941	-1.49191	0.00085	0.0120733
RSPH3	chr6:159398265-159421198	10.6477	5.70928	-0.899166	-1.2368	0.00515	0.0446089
TUBG2	chr17:40811265-40819024	29.7192	15.9342	-0.899266	-1.38481	0.002	0.0224405
KCNJ3	chr2:155555092-155714864	34.8209	18.6687	-0.89933	-1.18635	0.00345	0.0333771
ACSL6	chr5:131285666-131347761	33.1888	17.7855	-0.899996	-1.45829	0.00105	0.0140962

EFR3B	chr2:25264972-25382004	45.5509	24.4013	-0.900523	-1.45794	0.00065	0.00986869
DNM1L	chr12:32832133-32898584	41.8425	22.4038	-0.901224	-1.45281	0.0024	0.0253778
COPG1	chr3:128968452-128996616	40.5584	21.7042	-0.902025	-1.52174	0.0009	0.0126208
EFNB3	chr17:7608519-7614693	19.1921	10.2694	-0.902162	-1.37389	0.0017	0.0198318
SVEP1	chr9:113127528-113342160	2.44963	1.31057	-0.902367	-1.27679	0.0044	0.039835
PFKP	chr10:3109711-3178997	96.3102	51.518	-0.902611	-1.41872	0.0017	0.0198318
LY6H	chr8:144239330-144242053	140.777	75.3007	-0.902677	-1.45798	0.00095	0.0131856
SLC4A7	chr3:27414211-27525911	26.2201	14.0198	-0.903205	-1.43213	0.00205	0.0228705
CHCHD10	chr22:24105207-24110141	83.3327	44.5391	-0.90381	-1.31018	0.00385	0.0362521
ADCY9	chr16:4012649-4166186	17.5542	9.37414	-0.905056	-1.3475	0.00315	0.0310023
MYT1L	chr2:1792884-2335045	38.0896	20.3309	-0.905726	-1.40529	0.0017	0.0198318
ATP5C1	chr10:7830092-7849762	86.1034	45.9461	-0.906127	-1.47644	0.00065	0.00986869
LAPTM4B	chr8:98787808-98864830	38.3152	20.4283	-0.907347	-1.28421	0.0039	0.0365472
NRGN	chr11:124609828-124617102	108.164	57.5885	-0.909367	-1.40047	0.0017	0.0198318
GRAMD1B	chr11:123396343-123498479	35.545	18.9114	-0.910392	-1.47025	0.00135	0.0168292
SPTBN2	chr11:66452719-66488870	51.8526	27.5562	-0.912043	-1.424	0.0011	0.0145132
RUNDC3A	chr17:42385926-42396038	142.583	75.7369	-0.912732	-1.47184	0.00105	0.0140962
ATP6AP2	chrX:40440215-40465888	108.292	57.4836	-0.913704	-1.51664	0.001	0.0136643
PNMA2	chr8:26362195-26371483	118.121	62.6973	-0.913784	-1.31094	0.00325	0.0317912
DRP2	chrX:100474932-100519485	9.63533	5.11295	-0.914178	-1.46943	0.00145	0.0177865
B4GALNT1	chr12:58012187-58027022	39.3346	20.872	-0.914227	-1.39942	0.00195	0.0220762
NOS1AP	chr1:162039580-162339813	18.6258	9.88267	-0.914328	-1.4066	0.00185	0.0212581
DTD1	chr20:18568555-18744560	26.8374	14.2395	-0.914346	-1.35218	0.00205	0.0228705
DGKZ	chr11:46354454-46402104	40.4211	21.4431	-0.914597	-1.41968	0.00215	0.0235978
ULK4	chr3:41288089-42003660	6.58387	3.49199	-0.914886	-1.33532	0.002	0.0224405
ATP5F1	chr1:111982511-112004525	68.5967	36.3584	-0.915852	-1.35049	0.00265	0.0273487
PTPN5	chr11:18749474-18814268	30.9292	16.3766	-0.917334	-1.42923	0.0021	0.0232225
ATP6V1B2	chr8:20054703-20079207	108.944	57.6375	-0.918502	-1.44366	0.00105	0.0140962
ATP6V0B	chr1:44440601-44443972	112.483	59.4932	-0.91891	-1.49776	0.0009	0.0126208
PPP1R2	chr3:195241217-195270224	24.4752	12.9437	-0.919077	-1.4204	0.00135	0.0168292
DGKI	chr7:137074384-137531609	15.7224	8.31055	-0.919809	-1.48349	0.0006	0.00935947
CCSER1	chr4:91048683-92523370	14.1985	7.50382	-0.920041	-1.38256	0.00185	0.0212581
GSS	chr20:33516235-33543601	15.3091	8.08297	-0.921427	-1.3143	0.0048	0.0425388
SYNPO	chr5:149980641-150038792	99.5801	52.5392	-0.922463	-1.31	0.0034	0.0330021
SLC9A6	chrX:135067582-135129428	45.1658	23.793	-0.924694	-1.51813	0.0006	0.00935947
CCNC	chr6:99990262-100016690	19.9512	10.5063	-0.925226	-1.28814	0.0037	0.035159
NDN	chr15:23930553-23932450	35.1162	18.4824	-0.925986	-1.46676	0.00145	0.0177865
FBXL2	chr3:33318933-33428757	15.2949	8.04433	-0.92701	-1.42665	0.0011	0.0145132
HK1	chr10:71029755-71161637	78.7117	41.3377	-0.92912	-1.52541	0.00085	0.0120733
PCDH1	chr5:141232675-141257975	39.9333	20.9626	-0.929774	-1.5679	0.0005	0.00814556
RYR2	chr1:237205701-237997288	31.7608	16.6695	-0.930038	-1.36679	0.00225	0.024287
MOXD1	chr6:132617193-132722673	13.2661	6.96141	-0.930295	-1.30308	0.0035	0.0336944
ACTR10	chr14:58666832-58702353	25.6402	13.4539	-0.930387	-1.42125	0.00065	0.00986869
SYT12	chr11:66790189-66818334	10.3938	5.4537	-0.930413	-1.36671	0.00295	0.0296802
COPS8	chr2:237994083-238007489	40.4784	21.2342	-0.930761	-1.50419	0.0008	0.0115682
CDR2	chr16:22357256-22385938	10.2925	5.39625	-0.931558	-1.25238	0.0048	0.0425388
MAGEH1	chrX:55478521-55480001	49.4983	25.9508	-0.9316	-1.51485	0.00075	0.0109258
IMP3	chr15:75931425-75932664	28.6924	15.0371	-0.932145	-1.31742	0.0032	0.0313892

VLDLR	chr9:2535654-2654485	11.0401	5.78384	-0.932652	-1.3526	0.0033	0.0321732
HINT1	chr5:130494975-130501041	201.651	105.429	-0.935588	-1.54062	0.00045	0.00751151
KCNH1	chr1:210851656-211307457	9.19593	4.80716	-0.935812	-1.39557	0.0018	0.0207648
LNX1	chr4:54326436-54457753	13.4839	7.03909	-0.937778	-1.32602	0.0036	0.0344315
CDH22	chr20:44802371-44937137	10.1656	5.30283	-0.938856	-1.29009	0.00375	0.0355766
NOL6	chr9:33461350-33473941	12.3305	6.42657	-0.940111	-1.52392	0.0009	0.0126208
ALDOA	chr16:30064410-30081741	625.2	325.744	-0.940579	-1.2981	0.00415	0.0381398
WASL	chr7:123321980-123389125	48.5969	25.3006	-0.941693	-1.5828	0.0005	0.00814556
PTPRN2	chr7:157331749-158380482	59.4176	30.9323	-0.941778	-1.54394	0.001	0.0136643
KIAA0319	chr6:24544331-24646383	14.6787	7.64015	-0.942055	-1.52041	0.00095	0.0131856
ICA1	chr7:8152814-8302242	13.4967	7.02071	-0.942915	-1.34143	0.00495	0.0433451
AP2M1	chr3:183892633-183901879	143.777	74.7784	-0.943138	-1.52601	0.00105	0.0140962
CXADR	chr21:18885223-18965897	16.9463	8.80831	-0.944033	-1.4774	0.0006	0.00935947
NT5E	chr6:86159301-86205509	8.41466	4.37187	-0.944655	-1.33955	0.00335	0.0326065
KIAA1045	chr9:34958191-34982541	35.5372	18.4532	-0.945457	-1.54695	0.0003	0.00551994
CDH13	chr16:82660398-83830215	19.694	10.2236	-0.945854	-1.25065	0.0049	0.0430141
GDAP1	chr8:75262617-75279335	45.6053	23.6621	-0.946624	-1.5109	0.00045	0.00751151
ME3	chr11:86152149-86383678	22.1309	11.4811	-0.946797	-1.4803	0.00155	0.0185989
TMEM67	chr8:94767071-94831460	9.31547	4.83244	-0.946876	-1.27572	0.0037	0.035159
GPR61	chr1:110082493-110088455	6.24363	3.2378	-0.947373	-1.18551	0.006	0.0498407
FGD1	chrX:54471886-54522599	7.01389	3.63639	-0.947709	-1.29177	0.00415	0.0381398
LONRF2	chr2:100889752-100939195	64.877	33.6012	-0.949195	-1.24933	0.0054	0.0463417
TRUB2	chr9:131071395-131084697	24.8749	12.8766	-0.949946	-1.44088	0.00145	0.0177865
MAN1A1	chr6:119498365-119670931	20.908	10.8116	-0.951473	-1.56739	0.00015	0.00330506
EXOC6	chr10:94594469-94819251	16.183	8.36824	-0.951487	-1.52305	0.00075	0.0109258
ATP8A2	chr13:25946208-26595420	27.4865	14.2117	-0.951643	-1.57148	0.0003	0.00551994
ACP2	chr11:47260852-47290584	20.8421	10.7757	-0.951722	-1.25737	0.00285	0.028905
VDAC3	chr8:42249278-42263455	56.8927	29.4125	-0.951813	-1.53573	0.00075	0.0109258
LRFN5	chr14:42076763-42373752	16.1567	8.35186	-0.951963	-1.50074	0.0008	0.0115682
GABRB2	chr5:160715435-160975130	37.388	19.325	-0.952108	-1.49128	0.00065	0.00986869
OSTM1	chr6:108362612-108395941	21.157	10.9349	-0.952195	-1.20212	0.00505	0.0440021
TTC40	chr10:134621895-134756089	2.63216	1.3596	-0.953069	-1.2741	0.00235	0.024984
KCNQ5	chr6:73331570-73908573	11.9322	6.16275	-0.953212	-1.53895	0.00085	0.0120733
UQCRC1	chr3:48636431-48647098	71	36.6576	-0.953707	-1.6081	0.0007	0.010395
GSTA4	chr6:52842745-52860178	30.2411	15.5888	-0.955998	-1.4005	0.00055	0.00876569
GHITM	chr10:85899184-85913311	114.754	59.1446	-0.956222	-1.54644	0.00075	0.0109258
KCND2	chr7:119913721-120390387	14.8899	7.67431	-0.956227	-1.56087	0.0005	0.00814556
NDUFA3	chr19:54606159-54610281	168.44	86.8078	-0.956343	-1.41452	0.0007	0.010395
DGKH	chr13:42614171-42803891	13.2635	6.83189	-0.957106	-1.50328	0.00085	0.0120733
AFAP1	chr4:7755816-7941653	13.6278	7.01377	-0.958291	-1.53558	0.00055	0.00876569
DLD	chr7:107531551-107561643	27.8457	14.321	-0.959323	-1.5558	0.00055	0.00876569
KIAA1211L	chr2:99410308-99552684	23.0587	11.8508	-0.960329	-1.52555	0.00085	0.0120733
GPI	chr19:34855644-34893318	105.872	54.3779	-0.961222	-1.54599	0.00065	0.00986869
PCDH20	chr13:61983818-61989655	15.1458	7.77634	-0.961751	-1.43397	0.00195	0.0220762
PAM	chr5:102201526-102366808	41.1315	21.1061	-0.962582	-1.51459	0.00145	0.0177865
PSMD8	chr19:38865189-38874464	65.0086	33.3561	-0.962682	-1.61601	0.00015	0.00330506
UQCRC2	chr16:21964608-21994668	86.2754	44.2664	-0.962737	-1.62148	0.0003	0.00551994
SLC25A12	chr2:172639914-172750816	20.4092	10.4591	-0.964464	-1.56682	0.0004	0.00687883

TCEAL4	chrX:102840418-102842655	71.3132	36.5381	-0.964768	-1.43034	0.0035	0.0336944
ATP6V1D	chr14:67804580-67826720	68.4829	35.083	-0.964974	-1.58457	0.0003	0.00551994
GRIA3	chrX:122318095-122624766	53.4115	27.355	-0.965348	-1.48887	0.0007	0.010395
SLC12A5	chr20:44650328-44688789	60.6532	31.0597	-0.965541	-1.481	0.0009	0.0126208
CALN1	chr7:71244475-71877360	28.6172	14.6459	-0.96638	-1.54726	0.0005	0.00814556
TRAF5	chr1:211499956-211548286	6.41425	3.28041	-0.967404	-1.25514	0.00515	0.0446089
RAB36	chr22:23487512-23506531	8.22527	4.20611	-0.967577	-1.36862	0.0031	0.0306643
ACADSB	chr10:124768428-124817806	16.2464	8.30731	-0.96767	-1.60288	0.00015	0.00330506
MAP2K4	chr17:11924134-12047148	50.2868	25.7123	-0.967722	-1.60214	0.00075	0.0109258
RPGRIP1L	chr16:53633817-53737771	6.8436	3.49837	-0.968074	-1.45824	0.00125	0.0158974
SLC25A3	chr12:98987402-98995778	125.045	63.8323	-0.970094	-1.42569	0.00155	0.0185989
NRP1	chr10:33466418-33623833	19.6105	10.0063	-0.970715	-1.40885	0.0049	0.0430141
ARPC1A	chr7:98923495-98963885	58.8615	30.0262	-0.971103	-1.63736	0.00015	0.00330506
COX7B	chrX:77154960-77160881	150.427	76.7203	-0.971379	-1.52811	0.00055	0.00876569
SLC4A3	chr2:220492291-220506702	33.1194	16.8567	-0.97436	-1.59667	0.00035	0.00620045
FAM65B	chr6:24804508-25042396	20.0832	10.2158	-0.975185	-1.59415	0.0007	0.010395
ACTR1B	chr2:98272401-98280561	52.8261	26.835	-0.977138	-1.63838	0.0002	0.00407561
TSPYL1	chr6:116596021-116601280	109.397	55.5651	-0.977328	-1.50031	0.00045	0.00751151
GREB1L	chr18:18822202-19102791	5.82432	2.95715	-0.977881	-1.4243	0.00145	0.0177865
ITPR1	chr3:4535031-4889524	36.253	18.3962	-0.978694	-1.46967	0.0011	0.0145132
SIDT1	chr3:113251217-113348422	8.03711	4.07633	-0.979405	-1.45012	0.0014	0.0173787
EEF1A2	chr20:62119364-62130668	199.077	100.894	-0.980476	-1.56307	0.00045	0.00751151
SLITRK3	chr3:164904507-164914469	12.6238	6.39736	-0.980603	-1.53155	0.00075	0.0109258
TSPAN13	chr7:16793350-16824161	39.9483	20.2405	-0.980891	-1.4625	0.00075	0.0109258
AMIGO1	chr1:110049445-110052336	16.1318	8.16577	-0.982244	-1.48953	0.00125	0.0158974
TCEAL5	chrX:102528617-102531797	22.0392	11.1528	-0.982666	-1.40598	0.0015	0.0182223
KCNN2	chr5:113698015-113832197	22.8005	11.526	-0.984167	-1.51964	0.00065	0.00986869
LRRC7	chr1:70225857-70589171	38.8271	19.6147	-0.985131	-1.58612	0.0005	0.00814556
CADPS2	chr7:121958477-122526813	16.3581	8.26118	-0.985588	-1.61256	0.0003	0.00551994
PPFIA2	chr12:81651753-82153109	40.6905	20.532	-0.986822	-1.53414	0.00115	0.0149934
CSMD3	chr8:113235158-114449242	22.4178	11.3031	-0.987927	-1.55085	0.001	0.0136643
RAPGEFL1	chr17:38334241-38351906	27.8488	14.0347	-0.988622	-1.57415	0.0005	0.00814556
PKM	chr15:72491369-72523727	319.562	160.944	-0.989535	-1.374	0.00235	0.024984
PFKM	chr12:48436680-48540187	58.69	29.5176	-0.991539	-1.58291	0.0003	0.00551994
DIRAS1	chr19:2714564-2721390	26.6637	13.4089	-0.991682	-1.61822	0.0002	0.00407561
LPPR5	chr1:99355800-99614408	9.32431	4.68797	-0.992035	-1.3722	0.0031	0.0306643
JPH1	chr8:75146938-75233562	10.1896	5.11982	-0.992939	-1.46138	0.00105	0.0140962
TOMM20	chr1:235272657-235292256	115.99	58.2608	-0.993398	-1.5769	0.0005	0.00814556
HOMER1	chr5:78669646-78809659	32.9009	16.5216	-0.993769	-1.63079	0.0003	0.00551994
CDC40	chr6:110501623-110553422	15.5979	7.82183	-0.995777	-1.5847	0.00065	0.00986869
FAM49A	chr2:16730729-16847134	44.3136	22.2002	-0.997176	-1.5885	0.00025	0.00483727
QSOX1	chr1:180123967-180169859	24.2534	12.1484	-0.99742	-1.42717	0.00205	0.0228705
FBXO34	chr14:55738020-55820329	28.0296	14.0299	-0.998448	-1.67802	0.0002	0.00407561
MYL12B	chr18:3262110-3278282	116.545	58.3342	-0.998468	-1.64587	0.0002	0.00407561
MEIS3	chr19:47906374-47922785	38.9622	19.4943	-0.999021	-1.54644	0.0009	0.0126208
PFN2	chr3:149682690-149688741	231.909	115.964	-0.99988	-1.57334	0.0005	0.00814556
TARBP1	chr1:234527058-234614849	20.8145	10.4056	-1.00023	-1.66879	0.0002	0.00407561
C8orf34	chr8:69215702-69731258	10.9346	5.45617	-1.00295	-1.35789	0.00295	0.0296802

MAP1LC3A	chr20:33134687-33148149	70.4847	35.1516	-1.00372	-1.57926	0.00045	0.00751151
OPCML	chr11:132284874-133402403	31.5575	15.7308	-1.00439	-1.64235	0.0001	0.00238525
SGIP1	chr1:66999824-67210768	26.9607	13.4382	-1.00451	-1.66073	0.00035	0.00620045
GABBR2	chr9:101050363-101471479	48.7957	24.3086	-1.00529	-1.57285	0.0004	0.00687883
PCMT1	chr6:150070830-150132557	98.8784	49.1923	-1.00722	-1.60692	0.0004	0.00687883
TCEAL2	chrX:101380659-101382684	56.2024	27.956	-1.00747	-1.64102	0.0002	0.00407561
PNMA3	chrX:152224765-152228827	15.2851	7.60111	-1.00784	-1.56176	0.00085	0.0120733
OXCT1	chr5:41730166-41872338	47.0464	23.3727	-1.00926	-1.67746	0.0001	0.00238525
NDUFB1	chr14:92582467-92588153	123.431	61.2672	-1.01052	-1.40677	0.0011	0.0145132
OTUB1	chr11:63753324-63765892	46.3109	22.9785	-1.01107	-1.66722	0.0004	0.00687883
AGAP2	chr12:58118075-58135944	94.0541	46.6638	-1.01119	-1.38473	0.00175	0.0203076
B3GNT1	chr11:66112842-66115161	120.047	59.543	-1.0116	-1.67181	0.0003	0.00551994
DYNC2LI1	chr2:44001177-44037149	25.0539	12.4192	-1.01246	-1.34035	0.0038	0.0358579
STOML1	chr15:74275558-74286963	30.1493	14.9431	-1.01264	-1.57637	0.00105	0.0140962
GUCY1B3	chr4:156680124-156728794	45.6813	22.6298	-1.01338	-1.63344	0.00035	0.00620045
ISPD	chr7:16127151-16460947	7.34914	3.64061	-1.0134	-1.37216	0.0021	0.0232225
NETO2	chr16:47115430-47177936	13.1124	6.49401	-1.01375	-1.49127	0.00105	0.0140962
SLC9A7P1	chr12:98847618-98850923	4.15845	2.05632	-1.01598	-1.20588	0.00475	0.0422443
C16orf45	chr16:15528324-15682116	87.7981	43.3739	-1.01736	-1.67186	0.00005	0.00136643
CAMKK2	chr12:121675494-121736111	33.1962	16.3937	-1.01788	-1.55462	0.0005	0.00814556
SCG3	chr15:51973549-52013223	48.4918	23.9456	-1.01798	-1.64933	0.0001	0.00238525
ATP5O	chr21:35275756-35288158	114.713	56.6029	-1.01908	-1.66065	0.0003	0.00551994
SNURF	chr15:25068793-25223729	344.767	169.977	-1.02029	-1.37815	0.0006	0.00935947
MRPS18A	chr6:43638933-43655549	22.0392	10.8626	-1.0207	-1.34614	0.00315	0.0310023
TIMM17A	chr1:201924618-201939789	27.6207	13.6036	-1.02176	-1.4937	0.00065	0.00986869
UBE2QL1	chr5:6448735-6496834	15.1033	7.43551	-1.02236	-1.64313	0.0001	0.00238525
NDUFB5	chr3:179322574-179342288	72.9296	35.902	-1.02244	-1.55636	0.0006	0.00935947
MRC2	chr17:60704761-60770962	6.73267	3.31365	-1.02276	-1.34908	0.00205	0.0228705
LYPLA2	chr1:24117645-24122029	21.7422	10.6953	-1.02351	-1.44911	0.00155	0.0185989
RNF112	chr17:19314490-19320589	18.2206	8.96075	-1.02388	-1.61305	0.00045	0.00751151
UQCRH	chr1:46769379-46782447	118.479	58.2168	-1.02512	-1.54486	0.0008	0.0115682
B4GALT6	chr18:29202208-29264686	19.9756	9.80887	-1.02608	-1.62368	0.00035	0.00620045
DOC2B	chr17:6010-31421	24.598	12.076	-1.0264	-1.31788	0.00275	0.0281336
YWHAG	chr7:75956107-75988342	331.309	162.536	-1.02742	-1.34399	0.00145	0.0177865
PRKAR1B	chr7:588833-826116	109.162	53.5486	-1.02754	-1.58913	0.0004	0.00687883
RTN4R	chr22:20228937-20255816	13.5387	6.63897	-1.02806	-1.33141	0.0025	0.0262151
ARPC5L	chr9:127631483-127639696	33.3208	16.3334	-1.0286	-1.45619	0.00145	0.0177865
GLRB	chr4:157997276-158093242	27.3988	13.4216	-1.02956	-1.61356	0.00035	0.00620045
NDUFA5	chr7:123177051-123241705	28.6239	14.0086	-1.03091	-1.66864	0.00025	0.00483727
BCAS4	chr20:49411430-49493714	14.4041	7.04586	-1.03163	-1.24842	0.0056	0.0474801
KCNK1	chr1:233749749-233808258	41.3971	20.2345	-1.03271	-1.61817	0.0004	0.00687883
EEF1B2	chr2:207024317-207027653	128.584	62.8117	-1.0336	-1.39693	0.00315	0.0310023
ADRBK2	chr22:25960860-26125258	28.9867	14.147	-1.0349	-1.72159	0.00025	0.00483727
NT5DC3	chr12:104166080-104234975	30.024	14.6348	-1.03671	-1.70476	0.00015	0.00330506
PRDX2	chr19:12907633-12912724	204.639	99.7456	-1.03675	-1.78307	0.0001	0.00238525
FAM212B	chr1:112264685-112310199	14.8671	7.24633	-1.0368	-1.47043	0.0016	0.0190433
SNAP91	chr6:84262604-84419127	63.9722	31.1781	-1.03691	-1.62826	0.0008	0.0115682
MAPRE3	chr2:27193524-27250087	59.697	29.0879	-1.03724	-1.74887	0.00005	0.00136643

SYNJ1	chr21:34001068-34100351	41.5459	20.2292	-1.03827	-1.67016	0.00055	0.00876569
PNCK	chrX:152935187-152939816	37.6819	18.3283	-1.0398	-1.58666	0.00105	0.0140962
PPP3CB	chr10:75196185-75255782	82.6694	40.2024	-1.04007	-1.67123	0.00035	0.00620045
PEG10	chr7:94285636-94299006	110.599	53.7697	-1.04047	-1.31566	0.00505	0.0440022
SLC8A1	chr2:40144773-40739575	82.6083	40.1325	-1.04151	-1.51337	0.00105	0.0140962
NDUFAB1	chr16:23592334-23607639	71.4248	34.6788	-1.04237	-1.49547	0.00115	0.0149934
SNTB1	chr8:121547984-121824309	18.1769	8.82033	-1.0432	-1.32647	0.00275	0.0281336
ARF5	chr7:127228405-127231759	76.1994	36.947	-1.04432	-1.69267	0.00005	0.00136643
PPME1	chr11:73882107-73965748	34.5343	16.7416	-1.04459	-1.70259	0.0001	0.00238525
KCTD16	chr5:143550436-143856944	23.0285	11.1605	-1.04502	-1.68726	0.00005	0.00136643
SLC39A10	chr2:196521531-196602426	43.1773	20.9194	-1.04544	-1.53524	0.001	0.0136643
ALPK1	chr4:113218498-113363764	6.52296	3.15932	-1.04591	-1.4285	0.002	0.0224405
TBC1D30	chr12:65218351-65274798	8.12654	3.93564	-1.04604	-1.66121	0.00015	0.00330506
ATL1	chr14:50999799-51099784	45.5188	22.0318	-1.04688	-1.6397	0.0003	0.00551994
ATP6V0E2	chr7:149564782-149577801	144.812	70.0863	-1.04697	-1.65428	0.0003	0.00551994
NDUFA1	chrX:119004494-119010629	157.452	76.1678	-1.04766	-1.48472	0.0014	0.0173787
SLC2A13	chr12:40148822-40499661	28.0427	13.5507	-1.04926	-1.58347	0.0002	0.00407561
USMG5	chr10:105127723-105156270	176.106	85.0671	-1.04977	-1.42278	0.00275	0.0281336
SPRED3	chr19:38880839-38890523	3.66985	1.77216	-1.05021	-1.31738	0.0048	0.0425388
SV2B	chr15:91643181-91844539	52.2816	25.2334	-1.05097	-1.45616	0.00115	0.0149934
SV2A	chr1:149874871-149889434	76.5285	36.9262	-1.05135	-1.57517	0.0004	0.00687883
MDH2	chr7:75677336-75696827	59.5431	28.7139	-1.05219	-1.73252	0.00005	0.00136643
SYDE2	chr1:85623355-85666728	5.8019	2.79611	-1.0531	-1.38682	0.0033	0.0321732
ROBO2	chr3:77089293-77699114	8.66484	4.17583	-1.05311	-1.6631	0.00025	0.00483727
KIAA1467	chr12:13197314-13236383	30.2209	14.5573	-1.0538	-1.76619	0.0001	0.00238525
B3GALT1	chr2:168675181-168727366	10.5994	5.09765	-1.05608	-1.38676	0.00285	0.028905
HTR2A	chr13:47405676-47471211	9.45046	4.5408	-1.05744	-1.45075	0.0013	0.0163446
ARHGAP18	chr6:129898239-130031370	10.1654	4.88302	-1.05782	-1.46056	0.0003	0.00551994
KPNA2	chr17:66031847-66042970	21.2754	10.2167	-1.05826	-1.60531	0.00065	0.00986869
CACNA1E	chr1:181452685-181775921	43.0476	20.6575	-1.05927	-1.45209	0.00165	0.0194937
PRPS1	chrX:106871653-106894256	24.6671	11.8348	-1.05956	-1.59247	0.0006	0.00935947
CDK14	chr7:90225675-90839905	46.0771	22.0706	-1.06192	-1.72747	0.0002	0.00407561
FAM162A	chr3:122103022-122128961	43.0144	20.5907	-1.06282	-1.52801	0.00095	0.0131856
ATPIF1	chr1:28562601-28564616	117.081	56.0394	-1.06299	-1.43269	0.00235	0.024984
INA	chr10:105036919-105050108	34.3688	16.4368	-1.06417	-1.66096	0.0002	0.00407561
YWHAB	chr20:43514239-43537175	177.761	84.9537	-1.06519	-1.63955	0.0005	0.00814556
LINC00643	chr14:62584074-62606691	12.9456	6.18123	-1.0665	-1.6065	0.0003	0.00551994
MCTP1	chr5:94042288-94620279	27.3334	13.0508	-1.06652	-1.72881	0.00005	0.00136643
PGBD5	chr1:230457391-230561674	62.3017	29.7225	-1.06772	-1.75234	0.00005	0.00136643
NCEH1	chr3:172348434-172429008	29.1178	13.8883	-1.06803	-1.7419	0.00025	0.00483727
SNCA	chr4:90645249-90763142	57.3074	27.3321	-1.06813	-1.69813	0.00005	0.00136643
ADAM11	chr17:42836567-42859214	21.3057	10.1612	-1.06817	-1.68436	0.00005	0.00136643
COX7A2	chr6:75947390-75953644	134.935	64.2876	-1.06965	-1.67756	0.0002	0.00407561
SLITRK4	chrX:142710594-142723926	15.3525	7.30965	-1.0706	-1.67924	0.0001	0.00238525
HPRT1	chrX:133594174-133634698	32.3579	15.3908	-1.07205	-1.51779	0.00065	0.00986869
ARL6	chr3:97483364-97520086	13.5168	6.41503	-1.07523	-1.38617	0.0027	0.0277348
PGRMC1	chrX:118370207-118378429	106.327	50.4583	-1.07534	-1.74393	0.0002	0.00407561
CYP46A1	chr14:100150754-100193638	37.7387	17.8967	-1.07635	-1.71071	0.00005	0.00136643

CAMKK1	chr17:3763616-3796337	40.2825	19.0699	-1.07885	-1.7489	0.0002	0.00407561
LARGE	chr22:33669061-34316416	43.1869	20.4318	-1.07978	-1.72331	0.0001	0.00238525
UROS	chr10:127477146-127511837	39.1858	18.5328	-1.08025	-1.59127	0.00065	0.00986869
FAM3C	chr7:120988904-121036422	17.9467	8.47101	-1.08311	-1.47614	0.0011	0.0145132
SH2D5	chr1:21046224-21059133	12.9831	6.12614	-1.08359	-1.63075	0.00025	0.00483727
ANKRD42	chr11:82905290-82960013	9.76086	4.59825	-1.08592	-1.45467	0.002	0.0224405
PDE2A	chr11:72287183-72385497	64.7501	30.473	-1.08735	-1.66859	0.00035	0.00620045
PDHB	chr3:58413356-58419579	46.4731	21.8494	-1.0888	-1.70926	0.00005	0.00136643
PREPL	chr2:44502596-44589001	145.028	68.1371	-1.08982	-1.49191	0.00065	0.00986869
COL26A1	chr7:101006100-101202304	8.4629	3.96927	-1.09228	-1.40363	0.00155	0.0185989
SUB1	chr5:32585604-32604185	76.0514	35.6533	-1.09294	-1.75621	0.00005	0.00136643
DNAH6	chr2:84743578-85046713	6.23004	2.91958	-1.09348	-1.80956	0.00005	0.00136643
TPI1	chr12:6976583-6982521	237.575	111.243	-1.09466	-1.76557	0.00005	0.00136643
FARSB	chr2:223436161-223520827	20.1488	9.43372	-1.09479	-1.64949	0.0001	0.00238525
GABRA2	chr4:46251580-46392056	54.5706	25.5314	-1.09585	-1.72161	0.0001	0.00238525
RTN4RL1	chr17:1837970-1928178	13.3707	6.2554	-1.0959	-1.53941	0.00075	0.0109258
EPDR1	chr7:37960162-37991542	60.0983	28.1165	-1.09591	-1.72853	0.00025	0.00483727
FH	chr1:241660856-241683085	29.8859	13.9645	-1.09771	-1.71309	0.00015	0.00330506
LOC10065282	chr2:202937977-203061886	2.6467	1.23384	-1.10104	-1.32762	0.0022	0.0239231
RALYL	chr8:85095452-85834078	20.8078	9.69438	-1.10191	-1.57124	0.00115	0.0149934
SH3RF1	chr4:170015406-170192249	21.6879	10.0961	-1.1031	-1.83501	0.00005	0.00136643
TCTEX1D1	chr1:67218139-67244730	6.32673	2.94465	-1.10336	-1.21076	0.00585	0.0490104
TNFAIP8L3	chr15:51348798-51397473	12.2447	5.69372	-1.10472	-1.49097	0.0017	0.0198318
KCNT2	chr1:196194909-196577561	8.04675	3.74095	-1.105	-1.66855	0.00075	0.0109258
PNO1	chr2:68385004-68403094	13.6332	6.33744	-1.10515	-1.45904	0.0015	0.0182223
MAP2K1	chr15:66679210-66790146	77.2076	35.8865	-1.1053	-1.80659	0.0001	0.00238525
IDH3G	chrX:153051220-153063967	37.0545	17.2193	-1.10562	-1.33635	0.00535	0.0460471
TTC8	chr14:89290496-89344340	13.2687	6.15166	-1.10898	-1.54312	0.0012	0.015451
APOO	chrX:23851464-23926057	18.6391	8.64042	-1.10916	-1.38336	0.00435	0.0394838
PSD	chr10:104162373-104182893	190.645	88.3168	-1.11013	-1.51711	0.0013	0.0163446
CAMK2B	chr7:44256748-44365230	101.646	47.0146	-1.11237	-1.71913	0.00015	0.00330506
SCG2	chr2:224461657-224467217	46.4135	21.4637	-1.11264	-1.68041	0.00025	0.00483727
HCN1	chr5:45255051-45696220	10.8528	5.0129	-1.11435	-1.78435	0.00005	0.00136643
AGBL4	chr1:48998526-50489626	8.45814	3.9026	-1.1159	-1.36703	0.0038	0.0358579
HN1	chr17:73131337-73150778	31.0869	14.3436	-1.1159	-1.68627	0.00025	0.00483727
UQCRFS1	chr19:29698166-29704136	61.3632	28.3071	-1.11621	-1.75752	0.00025	0.00483727
RGS7BP	chr5:63801773-63908121	32.9641	15.1933	-1.11746	-1.83183	0.00005	0.00136643
CISD1	chr10:60028861-60049019	21.1053	9.72748	-1.11747	-1.58727	0.0007	0.010395
CACNB1	chr17:37329708-37353956	28.7409	13.2421	-1.11797	-1.81417	0.00005	0.00136643
STXBP1	chr9:130374485-130454995	140.79	64.8183	-1.11907	-1.695	0.0003	0.00551994
BDH1	chr3:197236653-197300194	13.3899	6.16289	-1.11946	-1.64339	0.0004	0.00687883
PGAM1	chr10:99186026-99193198	30.8523	14.1988	-1.1196	-1.70195	0.00025	0.00483727
DNAH9	chr17:11501747-11873065	7.45388	3.42856	-1.12039	-1.42412	0.00395	0.0368006
CPNE6	chr14:24540045-24547309	68.6696	31.5721	-1.12102	-1.84651	0.00005	0.00136643
COX6C	chr8:100890222-100906242	142.024	65.2301	-1.12253	-1.82174	0.00005	0.00136643
NAPA	chr19:47987538-48018515	56.9855	26.1576	-1.12337	-1.878	0.00005	0.00136643
LUZP2	chr11:24518515-25104186	16.155	7.4027	-1.12586	-1.73869	0.0001	0.00238525
PPP2R1A	chr19:52693054-52729678	134.484	61.5698	-1.12714	-1.84315	0.00005	0.00136643

CMAS	chr12:22199109-22218606	52.5062	24.0158	-1.12851	-1.76711	0.00005	0.00136643
PRDX5	chr11:64085559-64089295	215.127	98.2705	-1.13036	-1.81377	0.0001	0.00238525
RTN1	chr14:60062693-60337557	313.079	142.922	-1.1313	-1.49456	0.00135	0.0168292
GABRB3	chr15:26788693-27018935	54.3804	24.8235	-1.13138	-1.70563	0.00005	0.00136643
ACVR1C	chr2:158383278-158485399	8.77547	4.0028	-1.13247	-1.73158	0.0001	0.00238525
CRMP1	chr4:5822490-5894810	57.9677	26.4254	-1.13333	-1.92226	0.0002	0.00407561
C8orf46	chr8:67405490-67430759	103.383	47.1284	-1.13334	-1.80976	0.00005	0.00136643
ARNTL2	chr12:27485786-27599567	6.65696	3.03328	-1.13398	-1.67963	0.0007	0.010395
ATP5G3	chr2:176040985-176046490	39.1997	17.8593	-1.13417	-1.82759	0.00005	0.00136643
TUSC3	chr8:15397595-15624158	26.4754	12.0535	-1.13521	-1.81994	0.00015	0.00330506
XK	chrX:37545132-37591383	6.7555	3.07251	-1.13664	-1.46497	0.0011	0.0145132
NECAB1	chr8:91803920-91997485	55.669	25.3036	-1.13753	-1.68385	0.0001	0.00238525
GALNT9	chr12:132680916-132905905	21.9828	9.99181	-1.13756	-1.69826	0.00035	0.00620045
ATP6V0D1	chr16:67471916-67515089	87.3821	39.7038	-1.13806	-1.94151	0.00005	0.00136643
gen-01	chr2:17935176-17966632	3.0701	1.39269	-1.14041	-1.38323	0.0025	0.0262151
GRM7	chr3:6902801-7783218	13.1296	5.95342	-1.14103	-1.72936	0.0001	0.00238525
CPT1C	chr19:50194364-50216988	27.951	12.6397	-1.14494	-1.82464	0.00025	0.00483727
SYT16	chr14:62462540-62568427	22.9298	10.3678	-1.14511	-1.79656	0.0001	0.00238525
KCNA4	chr11:30031287-30038577	14.8596	6.71787	-1.14532	-1.71343	0.00005	0.00136643
LRP12	chr8:105501458-105601252	21.3023	9.62865	-1.1456	-1.84018	0.0002	0.00407561
NEGR1	chr1:71868624-72748277	42.2479	19.0827	-1.14662	-1.67743	0.0001	0.00238525
SYN2	chr3:12045833-12233532	118.534	53.5352	-1.14674	-1.56997	0.00085	0.0120733
SLCO1C1	chr12:20848288-20906320	22.0276	9.93961	-1.14805	-1.45624	0.00155	0.0185989
DMXL2	chr15:51739920-51914967	40.1943	18.1275	-1.14881	-1.80654	0.0001	0.00238525
SLC45A1	chr1:8378144-8404227	8.04967	3.629	-1.14936	-1.37829	0.00255	0.026597
NIPSNAP1	chr22:29950797-29977326	29.9174	13.4831	-1.14984	-1.81493	0.0001	0.00238525
GDA	chr9:74729510-74867140	58.9731	26.5591	-1.15085	-1.73352	0.00015	0.00330506
MPP7	chr10:28339922-28571067	3.53216	1.58933	-1.15213	-1.36302	0.00295	0.0296802
NEFM	chr8:24771273-24776606	79.5042	35.7309	-1.15386	-1.79099	0.0001	0.00238525
SLIT1	chr10:98757794-99052430	41.5773	18.6725	-1.15488	-1.70842	0.00025	0.00483727
C1orf95	chr1:226736500-226796915	47.2853	21.2325	-1.15512	-1.76338	0.00025	0.00483727
CYFIP2	chr5:156693089-156822606	107.768	48.3845	-1.15531	-1.68139	0.0002	0.00407561
FRRS1L	chr9:111899580-111929571	60.5757	27.1935	-1.15548	-1.73435	0.00015	0.00330506
FUCA1	chr1:24171571-24194859	10.1833	4.56995	-1.15595	-1.34858	0.0031	0.0306643
AP3B2	chr15:83211950-83378660	28.4195	12.7479	-1.15663	-1.65605	0.00035	0.00620045
C14orf2	chr14:104378624-104387903	110.826	49.6327	-1.15894	-1.74554	0.00015	0.00330506
PRICKLE1	chr12:42852139-42983572	12.4009	5.55351	-1.15897	-1.7281	0.00025	0.00483727
MOAP1	chr14:93648540-93651249	79.2241	35.4768	-1.15906	-1.91729	0.00005	0.00136643
ME2	chr18:48405431-48476162	28.2025	12.6118	-1.16104	-1.5833	0.0004	0.00687883
NDFIP2	chr13:80051498-80130212	49.0296	21.9136	-1.16183	-1.90511	0.0001	0.00238525
NDRG4	chr16:58497548-58547523	249.497	111.459	-1.16251	-1.67972	0.0007	0.010395
CTAGE5	chr14:39734475-39820397	20.23	9.01935	-1.1654	-1.43814	0.0022	0.0239231
ZNF804A	chr2:185463092-185804214	3.5473	1.58147	-1.16546	-1.43779	0.00235	0.024984
INPP5J	chr22:31518908-31530683	5.59879	2.49534	-1.16588	-1.34945	0.0042	0.0384589
PHACTR1	chr6:12717036-13295818	57.9519	25.7928	-1.16789	-1.90914	0.0001	0.00238525
KCNA1	chr12:5019072-5027422	11.6132	5.16569	-1.16873	-1.83245	0.0001	0.00238525
GABARAPL1	chr12:10365488-10375724	153.75	68.3789	-1.16896	-1.94756	0.00005	0.00136643
PRKCE	chr2:45879042-46415129	48.4867	21.5139	-1.17232	-1.88834	0.00005	0.00136643

HOPX	chr4:57514153-57547872	83.668	37.0754	-1.17421	-1.77825	0.0001	0.00238525
ZNF831	chr20:57766074-57834167	5.61933	2.48898	-1.17485	-1.74608	0.00015	0.00330506
CLSTN2	chr3:139654026-140286919	47.4958	21.0347	-1.17503	-1.88503	0.00005	0.00136643
FOCAD	chr9:20658307-20995954	36.7906	16.2935	-1.17504	-1.71511	0.00045	0.00751151
GABRD	chr1:1950767-1962192	18.502	8.17381	-1.1786	-1.62305	0.00015	0.00330506
KIAA1751	chr1:1884751-1935276	1.63852	0.723647	-1.17903	-1.25689	0.0051	0.0443283
CA10	chr17:49707673-50237377	22.0762	9.74746	-1.17939	-1.65814	0.00025	0.00483727
PCYOX1L	chr5:148737569-148749221	7.09569	3.13144	-1.18012	-1.44841	0.0015	0.0182223
TMEM59L	chr19:18723681-18731849	148.829	65.6601	-1.18057	-1.89554	0.00005	0.00136643
LRRIQ1	chr12:85430098-85638883	3.08642	1.36113	-1.18113	-1.42612	0.00185	0.0212581
RTN4RL2	chr11:57228009-57245012	10.3997	4.58392	-1.18189	-1.43798	0.0021	0.0232225
PIM2	chrX:48770458-48776413	10.3657	4.5689	-1.1819	-1.46225	0.00235	0.024984
MRPL21	chr11:68658745-68671303	24.2995	10.6863	-1.18516	-1.43228	0.0043	0.0391105
EXTL1	chr1:26348270-26362954	21.2522	9.34562	-1.18525	-1.90522	0.00005	0.00136643
GABRA5	chr15:27111865-27194357	55.0622	24.1887	-1.18673	-1.84766	0.00005	0.00136643
AK5	chr1:77747661-78025654	168.174	73.8601	-1.18708	-1.72915	0.00025	0.00483727
SPTB	chr14:65213000-65289866	15.8724	6.969	-1.1875	-1.83119	0.00005	0.00136643
CCKBR	chr11:6280903-6293357	6.63802	2.91422	-1.18764	-1.3265	0.0041	0.0378777
MLLT11	chr1:151032150-151040973	58.1696	25.5198	-1.18865	-1.88415	0.00005	0.00136643
CDHR3	chr7:105603656-105676877	3.31713	1.45465	-1.18927	-1.55269	0.00065	0.00986869
SLC6A7	chr5:149569519-149590635	8.91318	3.90774	-1.1896	-1.59041	0.00055	0.00876569
HTR7P1	chr12:13153375-13157764	2.86515	1.25606	-1.18971	-1.31908	0.0048	0.0425388
STIM2	chr4:26862312-27027003	15.4631	6.77629	-1.19026	-1.75978	0.00025	0.00483727
NPTN	chr15:73735498-73925753	157.478	68.9494	-1.19154	-1.7868	0.0001	0.00238525
PLCB1	chr20:8112911-8865547	32.1116	14.0529	-1.19223	-1.97336	0.00005	0.00136643
NDUFA4	chr7:10971579-10979813	109.651	47.974	-1.19259	-1.9553	0.00005	0.00136643
ADD2	chr2:70889215-70995375	59.1765	25.8893	-1.19267	-1.90921	0.00005	0.00136643
SPEF2	chr5:35617988-35814713	6.26814	2.74175	-1.19294	-1.48939	0.0007	0.010395
RIMS1	chr6:72596405-73112845	31.6402	13.8343	-1.1935	-1.90868	0.0001	0.00238525
NECAP1	chr12:8234806-8250373	58.6422	25.6024	-1.19566	-1.93426	0.00005	0.00136643
SLIT2	chr4:20255186-20622184	13.2559	5.78728	-1.19568	-1.91282	0.00005	0.00136643
CHSY3	chr5:129240522-129522327	5.29324	2.30822	-1.19737	-1.47249	0.00195	0.0220762
DAP	chr5:10679341-10761387	32.6965	14.2384	-1.19935	-1.5415	0.0017	0.0198318
TUNAR	chr14:96343108-96391908	9.03335	3.93305	-1.19961	-1.57909	0.0002	0.00407561
TMEM169	chr2:216946588-216967506	3.17816	1.3835	-1.19987	-1.34721	0.0043	0.0391105
MAP7D2	chrX:20024830-20135114	19.6199	8.53583	-1.20071	-1.83483	0.00005	0.00136643
DPCD	chr10:103348088-103369410	20.5417	8.93304	-1.20133	-1.46616	0.00195	0.0220762
MIR22HG	chr17:1614797-1619566	4.85332	2.11001	-1.20172	-1.27409	0.0054	0.0463417
MEST	chr7:130126015-130371406	42.8557	18.6298	-1.20188	-1.74505	0.00015	0.00330506
STXBP5L	chr3:120627049-121143608	30.2355	13.113	-1.20525	-1.9397	0.00005	0.00136643
TRAPPC2L	chr16:88923505-88927520	26.5192	11.4915	-1.20647	-1.46081	0.0014	0.0173787
PKP2	chr12:32943679-33049780	9.32265	4.03807	-1.20707	-1.66681	0.00015	0.00330506
DGAT2	chr11:75479777-75512581	5.24448	2.27048	-1.2078	-1.40355	0.0016	0.0190433
CCDC113	chr16:58283839-58328951	11.3948	4.93251	-1.20798	-1.90226	0.00005	0.00136643
RAB3B	chr1:52373627-52456436	20.2182	8.74384	-1.20932	-1.645	0.0002	0.00407561
CIRBP	chr19:1267469-1274809	180.792	78.1613	-1.20981	-1.70266	0.00005	0.00136643
RBM20	chr10:112404154-112599229	0.980943	0.423672	-1.21122	-1.21148	0.00515	0.0446089
LANCL2	chr7:55433140-55501435	30.7448	13.269	-1.21229	-2.01516	0.00005	0.00136643

LY6E	chr8:144099901-144103827	89.8392	38.7576	-1.21286	-1.91337	0.00005	0.00136643
PHYHIP	chr8:22077215-22089851	164.301	70.8572	-1.21336	-1.74928	0.0002	0.00407561
C1orf216	chr1:36179476-36184790	58.1714	25.0685	-1.21444	-1.98685	0.00005	0.00136643
SEZ6	chr17:27281946-27333458	23.4095	10.0801	-1.21559	-1.97702	0.00005	0.00136643
SEZ6L2	chr16:29882479-29910585	52.3163	22.5229	-1.21587	-1.95378	0.00005	0.00136643
GABRA1	chr5:161274196-161326965	44.3161	19.0691	-1.21659	-1.85392	0.0001	0.00238525
SLIT3	chr5:168088737-168728133	10.5536	4.5396	-1.2171	-1.94726	0.00005	0.00136643
SPRY1	chr4:124317949-124324915	8.07493	3.47242	-1.21751	-1.38072	0.00225	0.024287
ATP5B	chr12:57031958-57039852	234.265	100.697	-1.21812	-1.90373	0.00005	0.00136643
LINC01279	chr3:112315640-112320816	3.32259	1.42812	-1.21819	-1.28371	0.0037	0.035159
PNMA5	chrX:152157367-152162671	8.48489	3.64647	-1.21839	-1.68914	0.00045	0.00751151
WSCD2	chr12:108523510-108644313	23.853	10.2504	-1.21848	-1.98037	0.00005	0.00136643
ENO2	chr12:7023613-7032859	200.633	86.082	-1.22078	-1.84243	0.0001	0.00238525
SNX10	chr7:26331514-26413949	39.999	17.1545	-1.22137	-1.82089	0.0001	0.00238525
KCNQ3	chr8:133133104-133493004	22.7926	9.77037	-1.22208	-1.97229	0.00005	0.00136643
DNAL4	chr22:39174512-39190161	24.0731	10.3179	-1.22227	-1.734	0.0001	0.00238525
GRIA2	chr4:158141735-158287226	162.806	69.7562	-1.22276	-1.60948	0.00115	0.0149934
NCDN	chr1:36023392-36032380	286.543	122.751	-1.22302	-1.64054	0.00075	0.0109258
LOC728730	chr2:39664556-39828484	7.53543	3.22759	-1.22323	-1.51402	0.0012	0.015451
PDE1A	chr2:183004761-183387572	33.3967	14.302	-1.22349	-1.82931	0.00005	0.00136643
PIFO	chr1:111889194-111895639	14.1639	6.06182	-1.2244	-1.48748	0.00065	0.00986869
ANXA6	chr5:150480266-150537443	70.8078	30.2876	-1.22518	-2.06618	0.00005	0.00136643
SYT13	chr11:45261852-45307884	35.637	15.2348	-1.22601	-1.78965	0.00005	0.00136643
MICAL2	chr11:12132122-12285337	63.5013	27.1344	-1.22666	-1.70217	0.0001	0.00238525
C14orf79	chr14:105452615-105461855	7.99219	3.41507	-1.22667	-1.49436	0.00125	0.0158974
SCN8A	chr12:51985019-52206648	33.9283	14.4936	-1.22707	-1.93319	0.0001	0.00238525
FILIP1	chr6:76017799-76203545	6.01847	2.57041	-1.2274	-1.69361	0.0002	0.00407561
PLXNA4	chr7:131808090-132333447	26.5991	11.3502	-1.22866	-1.30278	0.00005	0.00136643
CNR1	chr6:88849584-88875767	32.6309	13.9091	-1.23022	-1.95209	0.00005	0.00136643
DGKB	chr7:14184673-14881075	33.667	14.3386	-1.23143	-1.80264	0.00005	0.00136643
THY1	chr11:119252487-119369944	224.274	95.4151	-1.23297	-1.80896	0.0002	0.00407561
FAM19A2	chr12:62102028-62586620	13.2925	5.65404	-1.23326	-1.77007	0.00005	0.00136643
LIN7B	chr19:49617617-49622397	19.1074	8.10912	-1.23651	-1.37561	0.0035	0.0336944
RASGRF2	chr5:80243511-81047072	26.7132	11.3349	-1.23678	-1.57941	0.00065	0.00986869
VDAC1	chr5:133307565-133340824	107.782	45.7274	-1.23698	-2.03395	0.00005	0.00136643
LMO4	chr1:87794150-87814607	39.833	16.8658	-1.23986	-2.09146	0.00005	0.00136643
RARRES2	chr7:150035406-150038763	56.9025	24.0843	-1.2404	-1.45549	0.0021	0.0232225
ACTL6B	chr7:100240725-100254084	16.2767	6.88565	-1.24114	-1.64905	0.0003	0.00551994
CNTN6	chr3:1134341-1445292	2.34313	0.99016	-1.2427	-1.29281	0.0059	0.0492421
RAB3C	chr5:57878938-58147406	58.8589	24.8724	-1.24272	-1.84631	0.00005	0.00136643
HS6ST2	chrX:131760037-132095423	11.013	4.6505	-1.24376	-1.6447	0.0005	0.00814556
TNFSF10	chr3:172223297-172241297	8.58824	3.62336	-1.24504	-1.35892	0.0022	0.0239231
LYPD1	chr2:133174146-133429070	20.6585	8.70208	-1.2473	-1.66786	0.00025	0.00483727
MAGEE1	chrX:75648045-75651746	24.5243	10.3302	-1.24735	-2.02123	0.00005	0.00136643
SMYD2	chr1:214454564-214510477	13.7794	5.79975	-1.24845	-1.65937	0.00065	0.00986869
OLFM1	chr9:137967088-138013030	233.244	98.0347	-1.25048	-1.6269	0.00035	0.00620045
KCNS3	chr2:18059113-18114225	3.98806	1.67616	-1.25052	-1.29583	0.0058	0.048777
CYP26B1	chr2:72356366-72374991	17.3761	7.30295	-1.25055	-1.44236	0.00445	0.0401639

HYDIN	chr16:70841286-71264625	7.23327	3.03415	-1.25335	-1.30838	0.0045	0.0404495
CHRNB2	chr1:154540256-154552353	18.2269	7.6406	-1.25431	-2.04136	0.00005	0.00136643
CACNA1H	chr16:1203240-1275254	10.6043	4.44152	-1.25553	-2.01097	0.00005	0.00136643
RBM3	chrX:48432740-48439553	13.7902	5.77536	-1.25566	-1.89512	0.00005	0.00136643
CDH9	chr5:26880708-27038689	13.5103	5.65251	-1.25709	-1.7591	0.00005	0.00136643
ATP5A1	chr18:43664109-43684199	239.155	99.9026	-1.25935	-1.85238	0.00005	0.00136643
DCC	chr18:49866541-51062273	6.18589	2.57742	-1.26305	-1.95762	0.00005	0.00136643
CGREF1	chr2:27309610-27341995	21.2744	8.86109	-1.26356	-1.52809	0.003	0.0299609
TRPC4	chr13:38210772-38443939	8.7401	3.63767	-1.26463	-1.75964	0.00025	0.00483727
GABRG2	chr5:161494647-161582545	40.1612	16.6764	-1.268	-1.94812	0.00005	0.00136643
ACOT7	chr1:6324331-6453826	71.1278	29.4981	-1.26979	-2.04321	0.00005	0.00136643
SLC1A6	chr19:15060844-15121455	9.40697	3.89834	-1.27087	-1.50418	0.0029	0.0292774
ATP5G1	chr17:46970147-46973232	91.8857	38.0514	-1.27189	-1.84633	0.0001	0.00238525
CCK	chr3:42299317-42307662	103.29	42.7369	-1.27315	-1.82081	0.00015	0.00330506
RGS2	chr1:192778168-192781407	27.4322	11.3371	-1.27481	-1.71133	0.00015	0.00330506
SLC8A2	chr19:47931278-47975307	30.8804	12.7566	-1.27545	-2.0135	0.00005	0.00136643
MARCH4	chr2:217122584-217236750	4.74804	1.95983	-1.27661	-1.56944	0.00105	0.0140962
NAP1L2	chrX:72432136-72434710	46.1122	19.0309	-1.27681	-2.0528	0.00005	0.00136643
TENM2	chr5:166711842-167691162	17.0689	7.0441	-1.27688	-2.07457	0.00005	0.00136643
GAD1	chr2:171673199-171717659	25.7574	10.6265	-1.27733	-1.91955	0.00005	0.00136643
PPP3R1	chr2:68405988-68479651	198.862	82.0227	-1.27767	-1.99442	0.00005	0.00136643
CADPS	chr3:62384020-62861064	55.9802	23.0758	-1.27854	-1.99718	0.00005	0.00136643
SYNPR	chr3:63263913-63602597	62.6091	25.7602	-1.28123	-2.01116	0.00005	0.00136643
SLC24A4	chr14:92788924-92967825	18.4253	7.5764	-1.28211	-1.6773	0.00115	0.0149934
LRRC9	chr14:60386430-60530277	2.31156	0.948918	-1.28451	-1.4361	0.0017	0.0198318
LINC00634	chr22:42348190-42354946	12.891	5.28659	-1.28595	-1.56144	0.00065	0.00986869
NETO1	chr18:70409548-70534810	19.6217	8.04648	-1.28602	-1.90384	0.00005	0.00136643
ARHGEF9	chrX:62854847-63005426	65.1284	26.6831	-1.28736	-2.06815	0.00005	0.00136643
SAMD5	chr6:147829827-147891157	2.95056	1.20797	-1.2884	-1.54159	0.0003	0.00551994
KCNIP2	chr10:103578824-103603677	44.2179	18.0899	-1.28945	-1.87386	0.0001	0.00238525
EPHA6	chr3:96533424-97467786	31.87	13.0302	-1.29034	-1.89409	0.0001	0.00238525
LINC00087	chrX:134229014-134232733	23.2109	9.48408	-1.29122	-2.00912	0.00005	0.00136643
ATP2B1	chr12:89981825-90049844	98.051	39.9825	-1.29416	-1.86306	0.0001	0.00238525
RAB27B	chr18:52495707-52562747	15.0217	6.12434	-1.29442	-1.89746	0.00005	0.00136643
TMEM35	chrX:100333835-100351355	33.2609	13.5579	-1.29469	-1.93408	0.00005	0.00136643
OTUB2	chr14:94492723-94515276	2.22024	0.90487	-1.29494	-1.40033	0.0013	0.0163446
SNCG	chr10:88718287-88723017	117.601	47.92	-1.2952	-1.97387	0.00005	0.00136643
LINC00839	chr10:42970938-42990785	3.78804	1.54236	-1.29631	-1.36473	0.00265	0.0273487
SPINT2	chr19:38755097-38783254	42.1408	17.1562	-1.29649	-2.00631	0.00005	0.00136643
LPPR2	chr19:11466061-11476374	70.4194	28.6608	-1.29689	-2.06806	0.00005	0.00136643
REEP1	chr2:86441119-86565206	26.7714	10.8916	-1.29747	-2.09515	0.00005	0.00136643
ATP1A1	chr1:116915794-116961244	189.407	77.0465	-1.29769	-1.74671	0.0001	0.00238525
TUBB3	chr16:89988416-90002505	107.747	43.7945	-1.29882	-2.15184	0.00005	0.00136643
MFSD4	chr1:205538111-205572046	52.1671	21.1832	-1.30022	-2.09561	0.00005	0.00136643
FAR2	chr12:29301935-29488549	9.87451	4.003	-1.30263	-1.77774	0.0004	0.00687883
CDK5	chr7:150750898-150755052	32.1186	13.0186	-1.30284	-1.81527	0.0002	0.00407561
PARM1	chr4:75858284-75975325	22.1088	8.96098	-1.30289	-2.1266	0.00005	0.00136643
CACNA2D1	chr7:81579417-82073031	40.5141	16.4177	-1.30317	-2.08994	0.00005	0.00136643

LOC728554	chr5:177302261-177311269	6.96675	2.82225	-1.30364	-1.45662	0.0015	0.0182223
VAV3	chr1:108113781-108537229	2.86477	1.15977	-1.30458	-1.43423	0.00115	0.0149934
PAK3	chrX:110187512-110464173	49.4216	19.9277	-1.31037	-2.13558	0.00005	0.00136643
SULT4A1	chr22:44220386-44258378	68.2928	27.5368	-1.31037	-2.13898	0.00005	0.00136643
GOT2	chr16:58741034-58768261	68.8365	27.7227	-1.31211	-2.01136	0.00005	0.00136643
ERICH3	chr1:75033794-75139422	15.2423	6.13674	-1.31253	-2.14213	0.00005	0.00136643
TMEM200A	chr6:130687425-130764210	9.09715	3.65246	-1.31655	-1.59836	0.00085	0.0120733
PSMA5	chr1:109941652-109969108	16.2047	6.50066	-1.31776	-1.96182	0.00005	0.00136643
STX1A	chr7:73113534-73134017	54.2589	21.74	-1.31951	-2.09959	0.00005	0.00136643
MMD	chr17:53469973-53499341	87.6584	35.0694	-1.32168	-2.12131	0.00005	0.00136643
KCNJ6	chr21:38996784-39288741	17.6242	7.03461	-1.32501	-1.9177	0.00005	0.00136643
PAK1	chr11:77033059-77185108	49.677	19.8263	-1.32516	-1.98669	0.00005	0.00136643
CHRM1	chr11:62676150-62689012	34.8099	13.8881	-1.32565	-2.16304	0.00005	0.00136643
NDST3	chr4:118955499-119179789	8.47147	3.37747	-1.32667	-1.99014	0.00005	0.00136643
HMGCR	chr5:74632992-74657926	39.5871	15.7796	-1.32697	-2.1629	0.00005	0.00136643
NGFRAP1	chrX:102631250-102633092	281.972	112.283	-1.32841	-2.20903	0.00005	0.00136643
TMEM198	chr2:220408744-220415317	13.5538	5.39405	-1.32926	-1.72571	0.0002	0.00407561
LINC00889	chrX:137696891-137699799	9.20496	3.66166	-1.32991	-1.66917	0.00055	0.00876569
NWD2	chr4:37246689-37451087	4.9521	1.96887	-1.33067	-1.89331	0.00005	0.00136643
HMGCS1	chr5:43287571-43313614	36.8008	14.6121	-1.33257	-2.07904	0.00005	0.00136643
RGS7	chr1:240938813-241520530	16.7974	6.65897	-1.33487	-1.9118	0.00005	0.00136643
CCDC80	chr3:112323232-112359990	11.3886	4.51374	-1.3352	-1.49756	0.00315	0.0310023
ATP6V1E1	chr22:18074902-18111588	135.562	53.7254	-1.33528	-2.16312	0.00005	0.00136643
RSPH1	chr21:43892596-43916464	12.2703	4.85846	-1.3366	-1.50062	0.00215	0.0235978
MAGED1	chrX:51546154-51645450	90.2303	35.7123	-1.33719	-2.17077	0.00005	0.00136643
ITGA8	chr10:15555947-15762334	6.03533	2.38806	-1.33759	-1.83748	0.00015	0.00330506
PCSK2	chr20:17206751-17465222	33.4631	13.2286	-1.33891	-2.08627	0.00005	0.00136643
PTGES	chr9:132500614-132515344	3.74368	1.47946	-1.33939	-1.26769	0.0049	0.0430141
OLFM3	chr1:102268122-102462790	13.0944	5.17261	-1.33999	-1.81959	0.00015	0.00330506
CALM3	chr19:47104511-47114039	560.308	221.103	-1.3415	-1.81908	0.00015	0.00330506
C6orf118	chr6:165693152-165723111	5.80236	2.28779	-1.34269	-1.47088	0.00155	0.0185989
ATP6V1A	chr3:113465865-113530905	123.657	48.7485	-1.34291	-1.98225	0.00005	0.00136643
NUP93	chr16:56764016-56878861	23.9456	9.43653	-1.34343	-2.17125	0.00005	0.00136643
TUBB4B	chr9:140135710-140142244	197.473	77.7419	-1.34489	-2.1678	0.00005	0.00136643
ARHGEF25	chr12:58003962-58011028	39.5005	15.5457	-1.34535	-2.14286	0.00005	0.00136643
ATP1B1	chr1:169075946-169396670	453.404	178.413	-1.34558	-1.78069	0.0001	0.00238525
RHBDD2	chr7:75508316-75518244	155.823	61.3154	-1.34558	-2.20493	0.00005	0.00136643
ADAMTS3	chr4:73146685-73434516	2.65675	1.04517	-1.34592	-1.46185	0.002	0.0224405
CLSPN	chr1:36197712-36235551	0.928888	0.365135	-1.34708	-1.46431	0.00205	0.0228705
NR4A3	chr9:102584136-102629173	6.81956	2.67949	-1.34772	-1.78542	0.0004	0.00687883
PRKCB	chr16:23847299-24231932	72.2649	28.3676	-1.34905	-1.81197	0.0002	0.00407561
RBP4	chr10:95351592-95360993	20.442	8.02244	-1.34942	-1.55413	0.00055	0.00876569
NEFL	chr8:24808468-24814383	161.836	63.3626	-1.35283	-1.93403	0.00005	0.00136643
MOSPD1	chrX:134021661-134049297	8.05292	3.15191	-1.35329	-1.48846	0.0005	0.00814556
PRMT8	chr12:3490514-3703138	4.5803	1.79106	-1.35463	-1.41401	0.0023	0.0246907
PTPRN	chr2:220154344-220174295	84.0542	32.8589	-1.35504	-2.18157	0.00005	0.00136643
LINC00086	chrX:134555867-134560225	25.5149	9.97212	-1.35537	-2.14208	0.00005	0.00136643
ELOVL4	chr6:80624528-80657315	17.1163	6.67876	-1.35772	-1.96679	0.00005	0.00136643

RASL10A	chr22:29708921-29711745	16.0827	6.26294	-1.3606	-1.52028	0.00245	0.0257982
C9orf16	chr9:130922538-130926207	129.295	50.2821	-1.36255	-2.13359	0.00005	0.00136643
NME5	chr5:137450860-137475132	13.6331	5.29773	-1.36366	-1.53525	0.0013	0.0163446
C10orf35	chr10:71390002-71393355	16.2953	6.32724	-1.36481	-1.64467	0.0005	0.00814556
TESC	chr12:117476727-117537251	17.06	6.62319	-1.36502	-1.67186	0.0004	0.00687883
RIN1	chr11:66099541-66104000	8.42678	3.27034	-1.36554	-1.65917	0.0006	0.00935947
BFSP1	chr20:17474549-17549865	1.5858	0.614878	-1.36684	-1.29674	0.0042	0.0384589
NMNAT2	chr1:183217371-183387634	42.4693	16.462	-1.36728	-2.20928	0.00005	0.00136643
C11orf70	chr11:101918168-101955291	4.3851	1.69968	-1.36734	-1.00949	0.00345	0.0333771
FAM131A	chr3:184053716-184079439	77.7252	30.1101	-1.36813	-2.15492	0.00005	0.00136643
YWHAZ	chr8:101930803-101965623	377.324	146.158	-1.36828	-1.7853	0.00035	0.00620045
DNAH10	chr12:124247041-124420267	1.91804	0.741313	-1.37148	-1.85829	0.0001	0.00238525
CNGA3	chr2:98962617-99015064	2.67262	1.03235	-1.37232	-1.33659	0.0035	0.0336944
ITFG1	chr16:47177978-47495015	72.2517	27.8909	-1.37324	-2.21812	0.00005	0.00136643
UNC13C	chr15:54305100-54920806	23.9755	9.24776	-1.37438	-2.03255	0.00005	0.00136643
TBC1D9	chr4:141541935-141677471	25.4431	9.81221	-1.37462	-2.19749	0.00005	0.00136643
LOC283070	chr10:12875132-12877545	48.6258	18.7508	-1.37477	-2.23898	0.00005	0.00136643
CHN1	chr2:175664041-175870107	351.569	135.268	-1.37798	-1.92193	0.00005	0.00136643
LDB2	chr4:16503164-16900424	34.1617	13.12	-1.3806	-2.21155	0.00005	0.00136643
RNF128	chrX:105937067-106040246	3.88171	1.49033	-1.38106	-1.48686	0.00085	0.0120733
ANXA4	chr2:69969126-70053596	21.2468	8.15217	-1.38199	-1.58271	0.0006	0.00935947
SLC17A7	chr19:49932654-49944808	241.279	92.4619	-1.38377	-1.93676	0.00005	0.00136643
STYK1	chr12:10771537-10826891	3.75602	1.43892	-1.38422	-1.50513	0.00065	0.00986869
PGM2L1	chr11:74041360-74109502	62.374	23.8695	-1.38578	-1.99784	0.00005	0.00136643
QPCT	chr2:37571752-37600465	8.31986	3.17961	-1.38771	-1.51601	0.0011	0.0145132
PCDP1	chr2:120302007-120414237	5.75044	2.19625	-1.38863	-1.58096	0.0016	0.0190433
PTK2B	chr8:27168998-27316908	81.3696	31.057	-1.38957	-2.21697	0.00005	0.00136643
NAPB	chr20:23355155-23402156	170.923	65.2331	-1.38967	-1.91248	0.00005	0.00136643
CNKSR2	chrX:21392535-21672813	67.541	25.6939	-1.39434	-2.16557	0.00005	0.00136643
TSPAN7	chrX:38420730-38548172	261.284	99.3849	-1.39452	-2.21033	0.00005	0.00136643
ITGA9	chr3:37493812-37903271	9.81524	3.72755	-1.3968	-1.98244	0.00005	0.00136643
LOC730101	chr6:52529198-52533951	2.15358	0.817169	-1.39803	-1.44696	0.0032	0.0313892
KIAA1257	chr3:128689781-128712986	2.44134	0.924804	-1.40045	-1.30258	0.0028	0.0284799
CCDC103	chr17:42977079-42981047	4.67769	1.77092	-1.4013	-1.41841	0.00545	0.0466799
LOC10028891	chr2:36581891-36582713	10.5403	3.98949	-1.40164	-1.34269	0.00385	0.0362521
TAGLN3	chr3:111717585-111732735	133.698	50.4625	-1.40569	-2.17259	0.00005	0.00136643
SEZ6L	chr22:26565439-26779563	69.8461	26.353	-1.40621	-2.09493	0.00005	0.00136643
UNC13A	chr19:17712136-17799008	49.6448	18.7245	-1.40671	-2.09748	0.0001	0.00238525
NT5DC1	chr6:116421998-116566853	29.1719	10.9973	-1.40742	-1.48864	0.00425	0.0387958
DNM1	chr9:130928343-131017527	188.371	71.008	-1.40753	-1.73513	0.00105	0.0140962
GRIN2B	chr12:13714409-14133022	65.9811	24.8668	-1.40783	-2.17276	0.00005	0.00136643
KCNG3	chr2:42669156-42721237	8.35517	3.14852	-1.40799	-1.7642	0.00035	0.00620045
SYT4	chr18:40847856-40857615	46.1433	17.3814	-1.40858	-2.22974	0.00005	0.00136643
VIP	chr6:153071931-153080902	7.08437	2.66729	-1.40927	-1.51481	0.00165	0.0194937
ECHDC3	chr10:11784355-11806065	6.63653	2.49839	-1.40943	-1.32924	0.00145	0.0177865
PLD3	chr19:40826966-40884397	217.396	81.837	-1.4095	-2.1796	0.00005	0.00136643
AP1S1	chr7:100797685-100804557	59.0109	22.2098	-1.40979	-2.07977	0.00005	0.00136643
DPP6	chr7:153584181-154686000	54.3448	20.4492	-1.4101	-2.36619	0.00005	0.00136643

PPL	chr16:4932507-4987136	8.2328	3.09018	-1.41369	-2.23657	0.00005	0.00136643
GRIN2A	chr16:9847261-10276611	37.5944	14.103	-1.41452	-2.06844	0.00005	0.00136643
SCART1	chr10:135267431-135281953	2.05394	0.770098	-1.41528	-1.23497	0.0058	0.048777
SYT1	chr12:79257772-79845788	261.981	98.1053	-1.41706	-1.77834	0.00035	0.00620045
PLS1	chr3:142315228-142432505	3.51239	1.31524	-1.41712	-1.29852	0.00395	0.0368006
FAM189A1	chr15:29412454-29862927	22.0355	8.24379	-1.41845	-1.99002	0.00005	0.00136643
TMEM215	chr9:32783496-32789199	1.36506	0.510565	-1.4188	-1.08724	0.0054	0.0463417
OSCP1	chr1:36883506-36916086	12.6003	4.70159	-1.42223	-1.73967	0.00015	0.00330506
PPP4R4	chr14:94640648-94746072	23.2476	8.66805	-1.4233	-1.92384	0.00005	0.00136643
HAR1A	chr20:61726844-61735737	2.71995	1.01239	-1.42581	-1.3269	0.00355	0.0340827
CAMK1G	chr1:209757044-209787284	17.1889	6.38003	-1.42984	-2.10985	0.00005	0.00136643
CENPF	chr1:214776531-214837914	1.08137	0.401115	-1.43078	-1.42086	0.002	0.0224405
EFHC2	chrX:44007127-44202923	5.61233	2.0815	-1.43097	-1.57284	0.00015	0.00330506
GAD2	chr10:26505235-26593491	10.5839	3.91676	-1.43414	-1.74633	0.0003	0.00551994
GLT1D1	chr12:129338080-129469509	11.6641	4.31261	-1.43545	-1.96633	0.00015	0.00330506
YWHAH	chr22:32329506-32353590	374.632	138.442	-1.43619	-1.91042	0.00005	0.00136643
CLSTN3	chr12:7282966-7311530	69.6264	25.7223	-1.43662	-2.34393	0.00005	0.00136643
PNPLA3	chr22:44319618-44343448	1.33168	0.491896	-1.43682	-1.28046	0.0019	0.0216212
TMEM130	chr7:98444110-98467673	126.318	46.6562	-1.43692	-2.24186	0.00005	0.00136643
KRT222	chr17:38811871-38821416	13.2481	4.88361	-1.43977	-1.78652	0.00015	0.00330506
ARMC3	chr10:23216952-23327452	7.81003	2.87845	-1.44004	-1.61165	0.001	0.0136643
C7orf63	chr7:89874487-89940377	4.97648	1.83089	-1.44258	-1.67925	0.00015	0.00330506
PPP3CA	chr4:101944586-102268628	159.885	58.81	-1.4429	-2.18894	0.00005	0.00136643
CAMKV	chr3:49895421-49907369	111.482	40.9501	-1.44488	-2.30497	0.00005	0.00136643
DUSP5	chr10:112257624-112271302	6.45268	2.36543	-1.44779	-1.61765	0.00045	0.00751151
CLEC2L	chr7:139208673-139229731	10.4814	3.8408	-1.44835	-1.58701	0.00035	0.00620045
DYNC1I1	chr7:95401817-95739634	57.7571	21.1027	-1.45257	-2.39363	0.00005	0.00136643
MET	chr7:116312458-116438440	14.2757	5.20471	-1.45567	-2.13501	0.00005	0.00136643
GOT1	chr10:101156626-101190530	75.0457	27.3065	-1.45853	-2.41865	0.00005	0.00136643
SCN2A	chr2:166095911-166248820	66.7806	24.2926	-1.45891	-2.07236	0.00005	0.00136643
ABRACL	chr6:139349818-139364439	15.8818	5.77353	-1.45985	-1.44539	0.0025	0.0262151
GNG2	chr14:52327021-52436518	70.919	25.7718	-1.46038	-2.17891	0.00005	0.00136643
CDH8	chr16:61685914-62070739	21.7171	7.88925	-1.46087	-2.33707	0.00005	0.00136643
OCLN	chr5:68788118-68853931	4.43548	1.61077	-1.46134	-1.55016	0.001	0.0136643
CD24	chrY:21094584-21438350	61.3058	22.2589	-1.46164	-2.19988	0.00005	0.00136643
TOMM34	chr20:43570770-43589114	31.1251	11.2839	-1.46381	-2.31289	0.00005	0.00136643
RASAL1	chr12:113536623-113574044	20.8125	7.54121	-1.46458	-2.23976	0.00005	0.00136643
RAB3A	chr19:18307610-18314874	97.1197	35.155	-1.46603	-2.4173	0.00005	0.00136643
CCDC170	chr6:151815174-151942328	3.30556	1.19496	-1.46794	-1.65125	0.00075	0.0109258
HUNK	chr21:33245627-33376377	3.11201	1.12491	-1.46803	-1.82995	0.0001	0.00238525
NOV	chr8:120428551-120436678	14.1765	5.1225	-1.46858	-2.07333	0.00005	0.00136643
PPP1R14C	chr6:150464187-150571528	6.53442	2.35724	-1.47096	-1.67219	0.00035	0.00620045
LINC00936	chr12:90102731-90105729	6.77604	2.4412	-1.47285	-1.65717	0.00035	0.00620045
DRD5	chr4:9783257-9785633	2.15255	0.77531	-1.4732	-1.34442	0.0015	0.0182223
SLC7A14	chr3:170177341-170303863	20.0286	7.19934	-1.47613	-2.38364	0.00005	0.00136643
CAMK1D	chr10:12391582-12871733	51.616	18.5513	-1.4763	-2.35103	0.00005	0.00136643
DUSP6	chr12:89741601-89746636	21.705	7.77754	-1.48064	-2.08175	0.00005	0.00136643
UCHL1	chr4:41258897-41270446	493.755	176.691	-1.48256	-2.25884	0.00005	0.00136643

CLYBL	chr13:100258917-100609419	6.41348	2.29079	-1.48526	-1.5167	0.0018	0.0207648
RFPL1S	chr22:29833003-29838444	23.4862	8.3886	-1.48531	-2.35623	0.00005	0.00136643
C11orf87	chr11:109292845-109299893	23.9242	8.54128	-1.48595	-2.33229	0.00005	0.00136643
CNTNAP2	chr7:145813452-148118088	38.8974	13.886	-1.48604	-2.18835	0.00005	0.00136643
C6orf165	chr6:88117689-88174191	4.32348	1.53828	-1.49088	-1.61815	0.00035	0.00620045
CAMK2A	chr5:149599053-149669403	465.202	165.39	-1.49198	-1.53461	0.0012	0.015451
NPY	chr7:24323806-24331484	38.9603	13.8101	-1.49628	-1.58094	0.00055	0.00876569
PRSS3	chr9:33750463-33799229	11.5308	4.08598	-1.49674	-1.54828	0.00175	0.0203076
ACTR3B	chr7:152456833-152552464	25.4208	9.00667	-1.49694	-2.20817	0.00005	0.00136643
HMP19	chr5:173472606-173536182	158.786	55.9269	-1.50547	-2.28738	0.00005	0.00136643
CHCHD6	chr3:126423062-126679263	32.3313	11.3842	-1.5059	-2.05721	0.00005	0.00136643
RASSF9	chr12:86198330-86230318	3.91626	1.37826	-1.50663	-1.49399	0.00225	0.024287
SCN3B	chr11:123499894-123525315	87.0702	30.5647	-1.51031	-2.27873	0.00005	0.00136643
PKD1L2	chr16:81134483-81253975	2.29285	0.803271	-1.51318	-1.41408	0.001	0.0136643
ZDHHC23	chr3:113666747-113681827	13.7841	4.82642	-1.51398	-2.22164	0.00005	0.00136643
RFK	chr9:79000432-79009444	46.1904	16.1517	-1.51591	-2.41075	0.00005	0.00136643
FAM101B	chr17:289770-295731	11.8076	4.12653	-1.51672	-1.75497	0.00035	0.00620045
CALY	chr10:135138927-135150475	72.9153	25.4396	-1.51915	-2.30449	0.00005	0.00136643
WDR66	chr12:122356462-122441832	5.00605	1.74501	-1.52044	-1.83769	0.00005	0.00136643
CKMT1A	chr15:43985083-43991420	13.9243	4.84534	-1.52294	-2.00992	0.00005	0.00136643
CLGN	chr4:141309606-141348815	2.28321	0.792796	-1.52604	-1.39365	0.0023	0.0246907
CACNG3	chr16:24266873-24373737	22.4143	7.77693	-1.52715	-2.195	0.00005	0.00136643
SLC4A10	chr2:162480844-162841786	65.9857	22.8777	-1.52821	-2.20129	0.00005	0.00136643
VWA3B	chr2:98703594-98929410	2.44994	0.849353	-1.52831	-1.71101	0.0001	0.00238525
FGF13	chrX:137713733-138287185	41.9195	14.5084	-1.53073	-2.42666	0.00005	0.00136643
TRPC5	chrX:111017541-111326004	4.74935	1.64095	-1.5332	-1.99493	0.00005	0.00136643
LMCD1	chr3:8543492-8609811	11.2517	3.88409	-1.53449	-1.68003	0.00025	0.00483727
ATP1A3	chr19:42470733-42498428	225.078	77.6795	-1.53482	-2.21349	0.00005	0.00136643
DLG3	chrX:69664704-69725339	28.4536	9.80528	-1.53698	-2.56309	0.00005	0.00136643
TNNT1	chr19:55644065-55660722	3.48523	1.19911	-1.53929	-1.45271	0.0017	0.0198318
LRGUK	chr7:133812104-133948933	3.02167	1.03803	-1.5415	-1.51009	0.00245	0.0257982
INPP5K	chr17:1397870-1420182	30.0202	10.2952	-1.54397	-1.91371	0.00015	0.00330506
EFCAB1	chr8:49627473-49647870	8.77937	3.00723	-1.54568	-1.71762	0.00085	0.0120733
BEX2	chrX:102564273-102565974	142.819	48.9187	-1.54573	-2.4303	0.00005	0.00136643
CHST8	chr19:34112860-34264414	7.23849	2.47896	-1.54595	-1.64633	0.00075	0.0109258
RASL11A	chr13:27844463-27847827	4.6232	1.58269	-1.54651	-1.52112	0.0013	0.0163446
AFF2	chrX:147582138-148082193	8.93118	3.04505	-1.55239	-2.52675	0.00005	0.00136643
WIPF3	chr7:29846169-29956682	23.4168	7.98042	-1.55301	-2.38358	0.00005	0.00136643
SULF1	chr8:70378858-70573147	13.0234	4.42702	-1.5567	-1.36101	0.0054	0.0463417
SLC15A4	chr12:129277738-129308541	26.0538	8.85077	-1.55762	-1.64684	0.0019	0.0216212
PDYN	chr20:1959401-1974931	24.4858	8.31805	-1.55763	-1.63507	0.00125	0.0158974
MDH1	chr2:63348534-63834330	211.224	71.7483	-1.55776	-2.34902	0.00005	0.00136643
ADAMTS16	chr5:5140442-5320412	2.45648	0.832241	-1.56152	-1.49673	0.00245	0.0257982
IL13RA2	chrX:114238537-114252207	5.2814	1.78921	-1.5616	-1.49436	0.0012	0.015451
VWC2	chr7:49813256-49952138	5.5618	1.88087	-1.56415	-1.47933	0.0026	0.0269589
IRF6	chr1:209958967-209979520	2.6053	0.881054	-1.56415	-1.63717	0.0006	0.00935947
GABRQ	chrX:151806636-151821825	11.3986	3.85283	-1.56487	-1.65164	0.00095	0.0131856
ST8SIA3	chr18:55019720-55036161	44.7559	15.1278	-1.56487	-2.33804	0.00005	0.00136643

MARVELD1	chr10:99473464-99477909	7.02227	2.36612	-1.56942	-1.72443	0.0002	0.00407561
WASF1	chr6:110421021-110501207	54.9165	18.4641	-1.57252	-2.5619	0.00005	0.00136643
GULP1	chr2:189156395-189460652	7.77098	2.60871	-1.57476	-1.89174	0.00035	0.00620045
FRK	chr6:116262692-116381921	4.9371	1.65634	-1.57566	-1.51063	0.0018	0.0207648
MIPEP	chr13:24304327-24471641	11.8852	3.98493	-1.57654	-1.79667	0.00005	0.00136643
KCTD4	chr13:45694630-45858239	24.3989	8.17209	-1.57804	-1.5422	0.005	0.0436961
DRD1	chr5:174867674-174871163	6.08876	2.03318	-1.58241	-1.8403	0.0001	0.00238525
PALMD	chr1:100111430-100231349	16.2206	5.40952	-1.58426	-2.07375	0.00005	0.00136643
ATP6V1H	chr8:54628102-54755871	109.101	36.3215	-1.58677	-2.45921	0.00005	0.00136643
YJEFN3	chr19:19639669-19648393	38.3135	12.7523	-1.58709	-1.89252	0.00005	0.00136643
POU3F1	chr1:38509522-38512450	5.15232	1.71169	-1.5898	-1.55659	0.0005	0.00814556
NELL1	chr11:20691096-21597229	16.4949	5.47611	-1.5908	-2.27747	0.00005	0.00136643
STC1	chr8:23699433-23712320	3.23946	1.07417	-1.59254	-1.36314	0.00195	0.0220762
ELMOD1	chr11:107461816-107537505	43.1631	14.2843	-1.59536	-2.46456	0.00005	0.00136643
FHL2	chr2:105977282-106055230	10.9382	3.61749	-1.59632	-1.78641	0.0002	0.00407561
EGR3	chr8:22545173-22550815	15.9622	5.27659	-1.59698	-2.18513	0.00005	0.00136643
SYT5	chr19:55684468-55691720	30.394	10.0084	-1.60257	-2.31155	0.00005	0.00136643
OCIAD2	chr4:48887396-48908845	19.3514	6.37158	-1.60271	-1.85668	0.00005	0.00136643
CHRM4	chr11:46406639-46408107	5.70785	1.87194	-1.60841	-1.60367	0.0015	0.0182223
F2RL1	chr5:76114832-76131140	2.9828	0.974884	-1.61337	-1.4019	0.0012	0.015451
GAP43	chr3:115342150-115440334	101.248	33.0671	-1.61442	-2.44866	0.00005	0.00136643
STMN2	chr8:80523048-80578410	161.341	52.6042	-1.61686	-2.45055	0.00005	0.00136643
EYA1	chr8:72109667-72274467	8.45095	2.75414	-1.61751	-1.62968	0.0011	0.0145132
FANK1	chr10:127585107-127698161	6.0628	1.97024	-1.62162	-1.57754	0.00105	0.0140962
SPATA17	chr1:217804694-218040484	4.98509	1.61643	-1.62481	-1.4913	0.00085	0.0120733
EFHB	chr3:19920965-19975706	2.97207	0.96346	-1.62517	-1.64899	0.0007	0.010395
BEX1	chrX:102317580-102319168	237.809	77.0726	-1.62551	-2.53073	0.00005	0.00136643
CCDC37	chr3:126113781-126155398	2.10366	0.680174	-1.62893	-1.50253	0.0006	0.00935947
TRHDE	chr12:72647286-73059422	11.6102	3.75321	-1.62919	-2.06043	0.00005	0.00136643
PRNCR1	chr8:128084938-128104840	0.34747	0.111922	-1.6344	-1.36309	0.0009	0.0126208
ALB	chr4:74269971-74287129	0.531294	0.171076	-1.63487	-1.20783	0.00325	0.0317912
C9orf116	chr9:138387025-138391761	10.544	3.39049	-1.63686	-1.44086	0.0035	0.0336944
CTSK	chr1:150768683-150780917	8.43888	2.70929	-1.63914	-1.52809	0.00115	0.0149934
SYTL3	chr6:159071045-159185908	4.7263	1.51549	-1.64093	-1.34364	0.0036	0.0344315
SPEF1	chr20:3758150-3762102	8.16419	2.61414	-1.64298	-1.8067	0.0002	0.00407561
CNIH3	chr1:224804178-224928249	17.5332	5.59438	-1.64804	-2.26356	0.00005	0.00136643
WDR96	chr10:105889645-105992120	9.9724	3.17913	-1.64931	-2.34404	0.00005	0.00136643
HOMER2	chr15:83517728-83621476	27.7707	8.84318	-1.65093	-2.15115	0.0001	0.00238525
RBFOX1	chr16:6069131-7763340	45.8453	14.5959	-1.65121	-2.53927	0.00005	0.00136643
HAPLN1	chr5:82934016-83016896	1.347	0.428014	-1.65402	-1.56129	0.00095	0.0131856
SVOP	chr12:109304654-109459045	43.3338	13.7664	-1.65434	-2.70091	0.00005	0.00136643
RHOD	chr11:66824288-66839488	4.16917	1.32261	-1.65637	-1.28377	0.0019	0.0216212
SLC6A20	chr3:45796940-45838035	8.22233	2.60344	-1.65913	-1.35849	0.0051	0.0443283
LRRC73	chr6:43474702-43478081	10.6658	3.37446	-1.66026	-1.73439	0.00025	0.00483727
RPE65	chr1:68894506-68915642	0.877091	0.276975	-1.66297	-1.26959	0.00265	0.0273487
TTC29	chr4:147628178-147867034	2.59899	0.820401	-1.66355	-1.52078	0.00075	0.0109258
FABP3	chr1:31838099-31845923	42.4572	13.3626	-1.66781	-2.44356	0.00005	0.00136643
DACH2	chrX:85403454-86087605	7.15873	2.25135	-1.66891	-1.85886	0.00045	0.00751151

EPHA4	chr2:222282746-222437010	47.2074	14.8331	-1.67019	-2.6961	0.00005	0.00136643
ENO4	chr10:118609022-118642112	1.9501	0.611131	-1.674	-1.4193	0.0051	0.0443283
SMOC2	chr6:168841830-169068674	6.48219	2.02542	-1.67826	-1.64189	0.0005	0.00814556
PTPRQ	chr12:80838125-81073968	0.56306	0.175678	-1.68036	-1.40094	0.00345	0.0333771
SCN11A	chr3:38887259-38995142	1.63698	0.510442	-1.68122	-1.45964	0.004	0.0371878
CLMP	chr11:122943032-123066007	5.63263	1.75535	-1.68205	-1.86282	0.0001	0.00238525
ANKRD33B	chr5:10564434-10657928	30.4653	9.4928	-1.68226	-2.61121	0.00005	0.00136643
CNIH2	chr11:66045671-66051685	84.2442	26.1827	-1.68596	-2.51033	0.00005	0.00136643
TMEM38A	chr19:16771937-16799816	22.6225	7.0235	-1.68749	-2.01998	0.00005	0.00136643
CCNO	chr5:54526980-54529508	3.61617	1.12199	-1.6884	-1.27221	0.0053	0.0457508
RGS14	chr5:176784843-176799599	32.1247	9.95582	-1.69007	-2.54267	0.00005	0.00136643
BCL11B	chr14:99635624-99738050	15.2222	4.7169	-1.69027	-2.66604	0.00005	0.00136643
ANKDD1B	chr5:74907300-74967671	0.709318	0.219693	-1.69094	-1.3483	0.0045	0.0404495
MAL2	chr8:120220609-120257914	29.4207	9.10227	-1.69253	-2.48446	0.00005	0.00136643
SLC17A6	chr11:22359666-22401046	8.81031	2.72165	-1.69471	-1.83052	0.00015	0.00330506
TUBA4A	chr2:220110191-220136910	72.8343	22.4819	-1.69585	-2.49288	0.00005	0.00136643
SNAP25	chr20:10004459-10288066	801.246	247.319	-1.69587	-1.99403	0.00005	0.00136643
PCDH19	chrX:99546641-99665271	20.5961	6.35615	-1.69614	-2.71985	0.00005	0.00136643
SUSD4	chr1:223394160-223537544	47.5774	14.6343	-1.70093	-2.55824	0.00005	0.00136643
MORN3	chr12:122089292-122107560	3.35436	1.03171	-1.701	-1.40517	0.0056	0.0474801
ATP2B3	chrX:152801579-152848387	26.2715	8.06974	-1.70291	-2.50189	0.00005	0.00136643
CCDC65	chr12:49297892-49315359	3.25503	0.998454	-1.7049	-1.68363	0.00025	0.00483727
TUBB2A	chr6:3153901-3157783	157.528	48.2456	-1.70714	-2.75045	0.00005	0.00136643
LBH	chr2:30454396-30482899	7.78189	2.37773	-1.71053	-2.04173	0.00005	0.00136643
DHRS11	chr17:34948225-34957233	6.70273	2.04273	-1.71425	-1.68333	0.0007	0.010395
PTH2R	chr2:209224568-209359231	3.78677	1.15364	-1.71477	-1.6395	0.0011	0.0145132
TRIM36	chr5:114460458-114516243	21.4562	6.53062	-1.7161	-2.17804	0.00005	0.00136643
PHACTR2	chr6:143929316-144152322	24.6821	7.49472	-1.71952	-1.69264	0.00285	0.028905
DCDC1	chr11:31284170-31391357	2.94191	0.892266	-1.72121	-1.58829	0.00025	0.00483727
AKAP5	chr14:64932216-64941221	24.3763	7.39136	-1.72157	-2.49381	0.00005	0.00136643
CPLX2	chr5:175223609-175311023	341.955	103.631	-1.72235	-2.04634	0.00005	0.00136643
DOK6	chr18:67068283-67516322	48.064	14.547	-1.72424	-2.69545	0.00005	0.00136643
PLEKHG4B	chr5:140372-190087	0.522969	0.15826	-1.72442	-1.49927	0.00065	0.00986869
SMPD3	chr16:68392229-68482409	19.4328	5.86251	-1.7289	-2.66552	0.00005	0.00136643
CAMK4	chr5:110559946-110820748	38.1281	11.4953	-1.72982	-2.70407	0.00005	0.00136643
TTC23L	chr5:34839268-34899564	1.94114	0.579817	-1.74324	-1.44171	0.0036	0.0344315
EFCAB12	chr3:129120163-129147494	2.40424	0.718129	-1.74326	-1.62566	0.00055	0.00876569
NSF	chr17_ctg5_hap1:195720-462618	87.3965	26.0451	-1.74657	-2.64262	0.00005	0.00136643
PLK2	chr5:57749809-57755966	70.9412	21.1341	-1.74705	-2.79315	0.00005	0.00136643
CAP2	chr6:17393735-17558023	72.0145	21.4209	-1.74927	-2.8405	0.00005	0.00136643
ZMYND10	chr3:50378536-50383156	9.67328	2.87089	-1.75251	-2.03623	0.00005	0.00136643
NEK10	chr3:27257096-27410912	7.1108	2.1097	-1.75297	-2.14218	0.00005	0.00136643
AGBL1	chr15:86685241-87572283	0.796468	0.235101	-1.76034	-1.4382	0.00355	0.0340827
EPCAM	chr2:47596286-47614167	6.49891	1.91558	-1.76242	-1.75054	0.00045	0.00751151
RLBP1	chr15:89753097-89764922	6.59449	1.94182	-1.76385	-1.79498	0.00015	0.00330506
ITGA4	chr2:182321618-182521834	2.96784	0.873719	-1.76417	-1.74939	0.00035	0.00620045
PPP1R32	chr11:61248584-61258400	5.91673	1.74035	-1.76542	-1.70171	0.0019	0.0216212
NSUN7	chr4:40751913-40812002	1.26531	0.372143	-1.76556	-1.35351	0.0051	0.0443283

EPHA5	chr4:66185280-66559104	23.1543	6.80969	-1.76562	-2.68118	0.00005	0.00136643
DNAH2	chr17:7623038-7737058	2.43663	0.715124	-1.76862	-2.43093	0.00005	0.00136643
SDK1	chr7:3341079-4308631	5.89654	1.72268	-1.77521	-2.0726	0.00015	0.00330506
ARMC4	chr10:28101092-28287984	2.48797	0.725865	-1.7772	-1.76518	0.0003	0.00551994
HS6ST3	chr13:96743092-97491816	23.4634	6.81186	-1.78429	-2.76936	0.00005	0.00136643
OGDHL	chr10:50942686-50970425	32.7673	9.50879	-1.78492	-2.80804	0.00005	0.00136643
KLK7	chr19:51479734-51487320	13.8473	4.01527	-1.78604	-2.08735	0.00015	0.00330506
AGPAT9	chr4:84457066-84527027	1.99663	0.578473	-1.78725	-1.55418	0.0023	0.0246907
IL1RAPL2	chrX:103810995-105011822	0.928299	0.268715	-1.78851	-1.29118	0.0059	0.0492421
KIT	chr4:55524094-55606881	28.0564	8.09786	-1.79272	-2.73693	0.00005	0.00136643
CDO1	chr5:115140429-115152405	51.7409	14.9099	-1.79504	-2.22577	0.00005	0.00136643
DNAH5	chr5:13690436-13944589	3.76833	1.08567	-1.79534	-2.4657	0.00005	0.00136643
SCG5	chr15:32933869-32989298	151.685	43.3448	-1.80714	-2.86003	0.00005	0.00136643
PPM1E	chr17:56833229-57184266	32.5084	9.27085	-1.81004	-1.72925	0.0004	0.00687883
CRABP2	chr1:156669399-156675608	9.16757	2.6142	-1.81017	-1.42497	0.0038	0.0358579
LRRC10B	chr11:61276271-61278490	8.9503	2.54988	-1.8115	-1.99126	0.00005	0.00136643
DIO2	chr14:80663867-80921810	19.471	5.54397	-1.81233	-2.60756	0.00005	0.00136643
C2orf80	chr2:209030070-209054773	17.9794	5.11786	-1.81274	-2.05348	0.00005	0.00136643
FHAD1	chr1:15573767-15724622	1.88384	0.536111	-1.81308	-1.83226	0.00045	0.00751151
ZCCHC12	chrX:117957786-117960931	75.288	21.4097	-1.81415	-2.1452	0.00005	0.00136643
DTHD1	chr4:36283236-36346407	3.76343	1.06794	-1.81722	-1.99447	0.0001	0.00238525
LYRM9	chr17:26205339-26220409	23.4107	6.64038	-1.81783	-2.4712	0.00005	0.00136643
WDR54	chr2:74648884-74652882	11.513	3.26324	-1.81889	-1.99476	0.00005	0.00136643
SLC4A2	chr7:150755298-150773614	48.673	13.7804	-1.82051	-1.77327	0.0012	0.015451
LAMP5	chr20:9485826-9511171	58.75	16.6282	-1.82096	-2.5302	0.00005	0.00136643
ENC1	chr5:73923230-74072737	327.289	92.5829	-1.82175	-1.96588	0.0001	0.00238525
TLL1	chr4:166794409-167025609	3.16571	0.894673	-1.8231	-1.73486	0.0004	0.00687883
CRYM	chr16:21269838-21329912	47.4385	13.3785	-1.82614	-2.65748	0.00005	0.00136643
ANO3	chr11:26353677-26684836	18.6745	5.25397	-1.82959	-2.74147	0.00005	0.00136643
EFCC1	chr3:128720471-128759585	1.72514	0.483751	-1.83438	-1.67841	0.00015	0.00330506
MAP3K19	chr2:135722060-135782248	2.21699	0.618294	-1.84224	-1.87121	0.00005	0.00136643
NTS	chr12:86268072-86276770	8.49943	2.36867	-1.84329	-1.54377	0.0005	0.00814556
C7orf57	chr7:48075107-48100894	2.54394	0.708553	-1.84411	-1.5911	0.0017	0.0198318
LAMB1	chr7:107564245-107643804	15.6616	4.35571	-1.84625	-2.86658	0.00005	0.00136643
NRN1	chr6:5998232-6007838	74.831	20.8041	-1.84677	-2.7495	0.00005	0.00136643
NID2	chr14:52471519-52535946	4.75074	1.31976	-1.84787	-1.74749	0.00015	0.00330506
AK7	chr14:96858447-96955764	3.89802	1.08098	-1.8504	-1.898	0.0002	0.00407561
FREM3	chr4:144498560-144621828	1.14241	0.316508	-1.85176	-1.49818	0.0023	0.0246907
ONECUT2	chr18:55102916-55158530	0.329634	0.0912258	-1.85335	-1.45691	0.0029	0.0292774
GABRA3	chrX:151335633-151619831	21.2591	5.87065	-1.85649	-2.77478	0.00005	0.00136643
C10orf67	chr10:23605519-23633772	0.787359	0.217133	-1.85844	-1.32444	0.00485	0.0427669
RTBDN	chr19:12936290-12946242	4.42378	1.21521	-1.86408	-1.62795	0.0004	0.00687883
RTP5	chr2:242811885-242815482	57.2337	15.6406	-1.87157	-2.66611	0.00005	0.00136643
VSTM2L	chr20:36531498-36573747	55.6721	15.1674	-1.87598	-2.83108	0.00005	0.00136643
C9orf24	chr9:34379016-34397849	21.4694	5.82285	-1.88249	-1.8899	0.0003	0.00551994
GRIA1	chr5:152870083-153193429	103.811	28.1265	-1.88396	-2.88832	0.00005	0.00136643
FKBP1B	chr2:24272583-24286550	31.3548	8.49087	-1.8847	-2.40885	0.00005	0.00136643
KCNC4-AS1	chr1:110751072-110752609	1.69447	0.457579	-1.88874	-1.30125	0.0013	0.0163446

LPPR4	chr1:99729847-99775138	97.6201	26.3074	-1.89171	-2.81002	0.00005	0.00136643
DRD2	chr11:113280316-113346001	1.68958	0.455255	-1.89192	-1.72512	0.0003	0.00551994
TTR	chr18:29171729-29178986	24.9561	6.72077	-1.89269	-1.48139	0.0001	0.00238525
KAZALD1	chr10:102820998-102825351	0.662553	0.17835	-1.89333	-1.41058	0.00095	0.0131856
CCDC180	chr9:100000707-100139577	3.92729	1.05387	-1.89783	-1.44763	0.003	0.0299609
GNG4	chr1:235710984-235814054	16.3307	4.38188	-1.89796	-2.58578	0.00005	0.00136643
CA14	chr1:150230217-150237480	5.44845	1.46102	-1.89887	-1.78189	0.0001	0.00238525
SV2C	chr5:75379304-75621416	3.4806	0.929258	-1.90518	-1.46235	0.00235	0.024984
STAC	chr3:36421978-36589498	1.87597	0.499719	-1.90845	-1.78564	0.0002	0.00407561
NLRP2	chr19:55476651-55512510	1.74889	0.465485	-1.90964	-1.85538	0.0001	0.00238525
C1QTNF5	chr11:119209643-119217383	33.4908	8.88877	-1.91371	-0.860276	0.0038	0.0358579
SLC4A5	chr2:74443368-74570534	5.73745	1.51499	-1.9211	-1.90656	0.0002	0.00407561
AGBL2	chr11:47681142-47736928	1.02348	0.270108	-1.92188	-1.60778	0.00175	0.0203076
ARC	chr8:143692404-143695833	8.12864	2.14417	-1.92259	-2.1535	0.00005	0.00136643
FAM160A1	chr4:152330397-152584784	2.73067	0.719196	-1.9248	-1.6965	0.00025	0.00483727
MUSK	chr9:113431050-113563278	1.25947	0.331372	-1.92629	-1.52043	0.0036	0.0344315
DNAH12	chr3:57327726-57530071	2.15686	0.567417	-1.92645	-2.03305	0.00005	0.00136643
HTR3A	chr11:113845796-113861034	1.07272	0.282042	-1.92729	-1.54881	0.001	0.0136643
OR1F1	chr16:3254246-3255185	0.893769	0.234607	-1.92966	-1.27413	0.00215	0.0235978
ICAM5	chr19:10400654-10407454	49.3259	12.9373	-1.93081	-2.98	0.00005	0.00136643
BARX2	chr11:129245880-129322174	1.201	0.31461	-1.93259	-1.36889	0.001	0.0136643
BTC	chr4:75671447-75719882	1.48034	0.387067	-1.93527	-1.37953	0.003	0.0299609
KCNV1	chr8:110979232-110986959	18.0836	4.72481	-1.93635	-2.52439	0.00005	0.00136643
FAM117A	chr17:47787686-47841518	10.0341	2.61293	-1.94118	-2.19461	0.00005	0.00136643
IL12RB2	chr1:67773046-67862583	5.74997	1.4942	-1.94418	-2.3072	0.00005	0.00136643
TRHR	chr8:110099652-110131812	6.3277	1.63916	-1.94873	-1.56184	0.0003	0.00551994
GPR83	chr11:94110476-94134585	6.26835	1.61855	-1.95338	-2.45612	0.00005	0.00136643
LRRC48	chr17:17876126-17920189	5.45905	1.40867	-1.95432	-2.13598	0.0001	0.00238525
FABP5	chr8:82192717-82197012	13.5919	3.50622	-1.95476	-1.70102	0.00005	0.00136643
EYA4	chr6:133562494-134210144	2.94096	0.749657	-1.97198	-1.98853	0.00015	0.00330506
RAB11FIP1	chr8:37716464-37757015	7.84691	1.9991	-1.97277	-2.05271	0.0001	0.00238525
FLJ41278	chr12:65277553-65371302	4.46797	1.13726	-1.97405	-2.01073	0.00015	0.00330506
OCA2	chr15:28000022-28344458	5.10446	1.29426	-1.97963	-1.90126	0.00005	0.00136643
CREG2	chr2:101964815-102003965	74.5956	18.9121	-1.97978	-3.16179	0.00005	0.00136643
PCDH8	chr13:53418108-53422775	14.9662	3.78881	-1.98189	-2.62604	0.00005	0.00136643
MYB	chr6:135502452-135540311	1.58388	0.40038	-1.98402	-1.65114	0.0005	0.00814556
SYP	chrX:49044264-49058913	219.791	55.5298	-1.9848	-3.04226	0.00005	0.00136643
CALB1	chr8:91070837-91095107	9.81658	2.47966	-1.98508	-2.28995	0.00005	0.00136643
WBSCR17	chr7:70597522-71178586	60.7785	15.326	-1.98758	-3.17948	0.00005	0.00136643
DNAJC5G	chr2:27498288-27504296	1.34931	0.339421	-1.99107	-1.51986	0.00045	0.00751151
SERTM1	chr13:37248048-37271975	27.2196	6.81939	-1.99693	-2.72206	0.00005	0.00136643
KCNK4	chr11:64058792-64067503	5.72748	1.42092	-2.01107	-1.99158	0.00005	0.00136643
LOC728084	chr12:89404902-89413469	0.714636	0.176925	-2.01407	-1.46606	0.0004	0.00687883
NELL2	chr12:44902057-45307711	149.928	36.9548	-2.02043	-3.06616	0.00005	0.00136643
PLA2G5	chr1:20396700-20418394	4.85857	1.19449	-2.02413	-1.80532	0.00015	0.00330506
GPD1	chr12:50497601-50505103	10.6983	2.6298	-2.02435	-2.54844	0.00005	0.00136643
BEX5	chrX:101408678-101410986	41.507	10.1624	-2.03011	-2.444	0.00005	0.00136643
C1QL3	chr10:16478941-16564004	54.448	13.269	-2.03682	-2.5081	0.00005	0.00136643

CACNG8	chr19:54466289-54493469	31.9341	7.73622	-2.0454	-3.25675	0.00005	0.00136643
LOC402160	chr4:2420671-2464690	0.89408	0.216091	-2.04877	-1.44283	0.00165	0.0194937
NPTXR	chr22:39214455-39240017	188.924	45.42	-2.05641	-2.58749	0.00005	0.00136643
RSPO3	chr6:127440047-127520626	7.42009	1.7819	-2.05802	-2.61503	0.00005	0.00136643
MORC1	chr3:108677086-108836993	0.446608	0.107244	-2.05812	-1.46674	0.00115	0.0149934
GPR26	chr10:125425870-125456913	8.61604	2.06347	-2.06195	-3.05655	0.00005	0.00136643
AMY2A	chr1:104159998-104168400	0.576148	0.137842	-2.06342	-1.42797	0.0026	0.0269589
ST6GALNAC2	chr17:74561460-74582145	7.83486	1.87064	-2.06638	-1.83384	0.00015	0.00330506
MCOLN3	chr1:85483764-85514223	6.24634	1.48649	-2.0711	-2.13464	0.00005	0.00136643
KCNAB1	chr3:155838336-156256927	32.6313	7.75654	-2.07277	-3.31466	0.00005	0.00136643
CYP1A1	chr15:75011882-75017877	0.313622	0.0740933	-2.08161	-1.37892	0.00095	0.0131856
BEST4	chr1:45249256-45253426	1.01103	0.238132	-2.086	-1.48927	0.00045	0.00751151
RIIAD1	chr1:151694012-151702082	4.05627	0.953357	-2.08906	-1.56267	0.0006	0.00935947
RSPH4A	chr6:116937641-116954148	5.0656	1.18784	-2.09239	-2.18213	0.00005	0.00136643
CCBE1	chr18:57098170-57364644	4.98108	1.16507	-2.09605	-2.67183	0.00005	0.00136643
CD36	chr7:80231503-80308593	6.34042	1.47936	-2.09961	-1.71132	0.0006	0.00935947
HRK	chr12:117298224-117319232	10.9955	2.56007	-2.10266	-2.05899	0.00005	0.00136643
KIRREL2	chr19:36347809-36358048	1.06992	0.248056	-2.10876	-1.72469	0.0009	0.0126208
FST	chr5:52776263-52782304	0.628834	0.145127	-2.11537	-1.41893	0.00105	0.0140962
IGFBP2	chr2:217498126-217529158	27.8041	6.37847	-2.12401	-2.15741	0.00005	0.00136643
SHISA6	chr17:11144739-11467380	19.6894	4.51018	-2.12616	-2.85687	0.00005	0.00136643
NNAT	chr20:36145818-36156333	177.89	40.7078	-2.12761	-1.85625	0.00165	0.0194937
ALDH1A3	chr15:101420008-101456830	6.61124	1.5076	-2.13267	-2.18489	0.00005	0.00136643
EGR1	chr5:137801180-137805004	15.1492	3.44252	-2.1377	-2.52151	0.00005	0.00136643
TPBGL	chr11:74951949-74954749	0.751724	0.170063	-2.14414	-1.47665	0.0012	0.015451
ODF3B	chr22:50968837-50971008	4.61359	1.04252	-2.14582	-1.72254	0.0005	0.00814556
FNDC5	chr1:33327868-33338093	15.5839	3.51862	-2.14697	-2.45397	0.00005	0.00136643
CHRNB4	chr15:78916635-78933587	0.302186	0.0680995	-2.14972	-1.26004	0.00565	0.0476976
LOC10272363	chrX:134945650-134975579	0.478641	0.107814	-2.1504	-1.14546	0.00315	0.0310023
PRKCG	chr19:54385466-54410901	86.2208	19.4137	-2.15096	-3.40885	0.00005	0.00136643
SLC22A25	chr11:62931295-62997124	0.411847	0.0926873	-2.15167	-1.17235	0.0051	0.0443283
PTPN3	chr9:112137973-112260593	7.59926	1.69173	-2.16736	-2.75455	0.00005	0.00136643
KLHDC8A	chr1:205305192-205326218	7.64151	1.69288	-2.17438	-2.55793	0.00005	0.00136643
MIR7-3HG	chr19:4769116-4772568	9.23096	2.02925	-2.18553	-1.74791	0.0007	0.010395
CABP7	chr22:30116343-30162969	33.6816	7.39128	-2.18807	-2.30481	0.0005	0.00814556
NEK2	chr1:211831598-211848972	0.621921	0.136042	-2.19268	-1.57843	0.00105	0.0140962
TMPRSS13	chr11:117771355-117800168	0.487005	0.106351	-2.1951	-1.38709	0.00355	0.0340827
ZBBX	chr3:166958076-167098085	8.10083	1.7444	-2.21534	-2.61979	0.00005	0.00136643
DNAAF3	chr19:55670028-55678090	1.60889	0.345484	-2.21937	-1.88595	0.0002	0.00407561
SPHKAP	chr2:228844669-229046361	40.7833	8.72516	-2.22473	-3.27642	0.00005	0.00136643
NEUROD1	chr2:182540832-182545392	5.77605	1.22827	-2.23346	-2.32972	0.00005	0.00136643
CCDC19	chr1:159842153-159869906	3.93379	0.834682	-2.23662	-1.93379	0.00055	0.00876569
VGF	chr7:100805789-100808852	14.8958	3.15641	-2.23855	-2.595	0.00005	0.00136643
GUCA1A	chr6:42123143-42147794	1.55754	0.329009	-2.24308	-1.73978	0.00005	0.00136643
LOC400891	chr22:21400248-21418457	0.574761	0.121378	-2.24346	-1.72475	0.00045	0.00751151
ANKRD26P3	chr13:19836939-19919113	2.94331	0.617518	-2.25289	-1.98204	0.00005	0.00136643
SYTL1	chr1:27668482-27680423	3.96238	0.822936	-2.26752	-2.18835	0.00005	0.00136643
HPGD	chr4:175411327-175444049	2.44775	0.50805	-2.26842	-1.77226	0.0003	0.00551994

GPR64	chrX:19007424-19140755	0.471466	0.0970322	-2.28062	-1.80073	0.00005	0.00136643
BMP4	chr14:54416454-54423554	4.88367	1.0005	-2.28725	-1.77408	0.0011	0.0145132
KLHL14	chr18:30252633-30352974	0.353045	0.0722447	-2.28889	-1.71934	0.00005	0.00136643
DAO	chr12:109273856-109294710	0.789851	0.16112	-2.29345	-1.5799	0.0004	0.00687883
STRIP2	chr7:129074273-129128239	16.2093	3.2971	-2.29756	-2.40535	0.00005	0.00136643
TEKT1	chr17:6703299-6735060	6.52256	1.32585	-2.29852	-2.00129	0.00045	0.00751151
SLC27A2	chr15:50474392-50528589	5.71384	1.16136	-2.29864	-2.1631	0.00005	0.00136643
SLC2A12	chr6:134308718-134373789	25.9113	5.24329	-2.30504	-2.20124	0.0001	0.00238525
MYLK3	chr16:46736193-46782221	3.98576	0.805954	-2.30608	-2.44645	0.0001	0.00238525
CYP24A1	chr20:52769987-52790516	0.559459	0.113075	-2.30676	-1.34669	0.00585	0.0490104
NUP62CL	chrX:106366656-106449670	2.00816	0.405765	-2.30716	-1.5401	0.0035	0.0336944
SLC16A10	chr6:111408780-111544606	9.33281	1.87255	-2.31731	-2.19219	0.00005	0.00136643
PROC	chr2:128175995-128186822	0.802018	0.160673	-2.31951	-1.59722	0.00085	0.0120733
SLC38A8	chr16:84043271-84075762	1.52884	0.3053	-2.32413	-1.45733	0.0042	0.0384589
NRIP3	chr11:9002122-9025596	49.553	9.88809	-2.32521	-3.66323	0.00005	0.00136643
CEL	chr9:135937364-135947250	3.99811	0.796941	-2.32677	-1.90143	0.00005	0.00136643
ATOH7	chr10:69990351-69991870	2.70756	0.539623	-2.32697	-1.76033	0.00005	0.00136643
WDR16	chr17:9479943-9546776	6.85527	1.36236	-2.33111	-2.3163	0.00005	0.00136643
COL25A1	chr4:109731876-110223799	15.5054	3.05872	-2.34177	-2.62422	0.00005	0.00136643
SLC24A3	chr20:19193289-19703541	39.8491	7.85666	-2.34256	-3.56939	0.00005	0.00136643
MYO5B	chr18:47340392-47721451	2.85531	0.561675	-2.34584	-1.50434	0.00395	0.0368006
ANKRD20A19	chr13:24481422-24523454	7.41144	1.45617	-2.34757	-1.89326	0.0003	0.00551994
SAMD3	chr6:130465446-130686570	2.92804	0.574067	-2.35064	-2.04514	0.0017	0.0198318
EGR2	chr10:64571755-64578927	1.75655	0.34323	-2.3555	-1.97527	0.00005	0.00136643
EGR4	chr2:73518056-73520829	3.72949	0.7255	-2.36193	-1.88991	0.00005	0.00136643
COL9A1	chr6:70925742-71012786	16.9327	3.28972	-2.36377	-2.30103	0.00005	0.00136643
LOC440896	chr9:69174213-69181041	3.02494	0.587695	-2.36377	-1.91104	0.00005	0.00136643
RORC	chr1:151778546-151804348	0.788383	0.152221	-2.37273	-1.66268	0.00125	0.0158974
CLDN16	chr3:190105660-190129932	0.388233	0.0738437	-2.39437	-1.58873	0.00045	0.00751151
DYDC2	chr10:82095860-82127829	3.62616	0.689689	-2.39442	-2.06899	0.00025	0.00483727
C15orf26	chr15:81426643-81441516	3.7064	0.704075	-2.39622	-1.86061	0.0002	0.00407561
NPY2R	chr4:156129780-156138228	4.40205	0.835355	-2.39771	-2.33107	0.00005	0.00136643
ABCA13	chr7:48211056-48687091	0.405311	0.0768393	-2.39912	-2.07679	0.00005	0.00136643
ST6GALNAC5	chr1:77333185-77529737	46.6956	8.77403	-2.41198	-3.52981	0.00005	0.00136643
ROPN1L	chr5:10441973-10465138	6.25546	1.17305	-2.41485	-2.14159	0.0011	0.0145132
CPNE4	chr3:131252403-131758450	13.2423	2.4569	-2.43024	-3.27414	0.00005	0.00136643
MAS1	chr6:160327973-160329107	20.8516	3.8563	-2.43487	-2.53488	0.00005	0.00136643
PCP4	chr21:41239346-41301322	51.8519	9.56091	-2.43918	-2.40004	0.00005	0.00136643
DAW1	chr2:228736326-228789026	8.37019	1.53703	-2.44512	-2.02854	0.0002	0.00407561
KCNE1L	chrX:108866928-108868393	1.96627	0.355892	-2.46595	-1.89755	0.00005	0.00136643
PRSS12	chr4:119201192-119273922	4.63256	0.837774	-2.46718	-2.54007	0.00005	0.00136643
CLDN1	chr3:190023489-190040235	3.74156	0.674671	-2.47138	-2.21298	0.00005	0.00136643
IGFN1	chr1:201159952-201198080	0.798403	0.143741	-2.47365	-2.05345	0.00005	0.00136643
INHBA-AS1	chr7:41728600-41818976	9.00941	1.61442	-2.48042	-2.01677	0.0007	0.010395
C1orf194	chr1:109648572-109656479	4.5732	0.818961	-2.48134	-1.94339	0.00005	0.00136643
HLA-C	chr6_ssto_hap7:2570004-2573382	4.04058	0.723431	-2.48164	-1.73686	0.0021	0.0232225
DNAI1	chr9:34458749-34520987	2.18879	0.39181	-2.48191	-2.12046	0.00005	0.00136643
CCDC114	chr19:48799708-48823332	3.00215	0.536949	-2.48314	-2.42632	0.00005	0.00136643

CAPSL	chr5:35904397-35938881	7.338	1.31145	-2.48423	-2.15031	0.00005	0.00136643
CHGB	chr20:5891973-5906005	116.096	20.6325	-2.49233	-3.92358	0.00005	0.00136643
GPR157	chr1:9164475-9189229	1.70281	0.301294	-2.49868	-1.55209	0.0007	0.010395
TNNI3	chr19:55663135-55669100	0.622718	0.110146	-2.49916	-1.39303	0.00585	0.0490104
KL	chr13:33590570-33640282	12.1667	2.12095	-2.52016	-2.97471	0.00005	0.00136643
SYTL5	chrX:37865834-37988073	12.7355	2.21531	-2.52327	-3.28547	0.00005	0.00136643
CDC42BPG	chr11:64591661-64612041	0.598662	0.103951	-2.52584	-1.53075	0.00395	0.0368006
MAPK15	chr8:144798506-144804633	7.69612	1.33169	-2.53087	-2.59616	0.00005	0.00136643
CCDC153	chr11:119060962-119066584	2.35264	0.402379	-2.54766	-1.61128	0.0056	0.0474801
FAM19A1	chr3:68040733-68594771	24.3695	4.1601	-2.55039	-3.26236	0.00005	0.00136643
TH	chr11:2185158-2193035	0.567014	0.0967578	-2.55094	-1.3713	0.00135	0.0168292
LINC01314	chr15:80487820-80544603	36.7253	6.25781	-2.55305	-3.76408	0.00005	0.00136643
GLB1L2	chr11:134201767-134246218	4.31494	0.733669	-2.55614	-2.27854	0.00005	0.00136643
TMEM45B	chr11:129685740-129729898	2.49179	0.41944	-2.57064	-1.94136	0.00015	0.00330506
VAT1L	chr16:77822482-78014001	50.43	8.39122	-2.58733	-2.8278	0.00005	0.00136643
BNC1	chr15:83924654-83953468	0.233804	0.0388558	-2.58909	-1.77017	0.0001	0.00238525
CCDC108	chr2:219866936-219906273	3.08728	0.512654	-2.59028	-2.27712	0.0002	0.00407561
GALNT11	chr7:151722777-151819427	109.152	18.1062	-2.59179	-2.52866	0.00005	0.00136643
CDH3	chr16:68678150-68732957	5.20994	0.862607	-2.59449	-2.46359	0.00005	0.00136643
ADTRP	chr6:11713887-11779280	4.89894	0.809918	-2.59662	-1.88497	0.00005	0.00136643
IL5RA	chr3:3108007-3152058	2.5002	0.410945	-2.60503	-1.97673	0.00005	0.00136643
BDNF	chr11:27528398-27743605	5.57072	0.913987	-2.60762	-2.17008	0.0002	0.00407561
LOC10013207	chr11:111284966-111288911	0.369121	0.0603286	-2.61318	-1.36643	0.00325	0.0317912
WDR63	chr1:85527980-85598821	4.16496	0.673718	-2.62808	-2.5103	0.00005	0.00136643
TRPM3	chr9:73149965-73736514	116.093	18.6994	-2.63422	-2.00169	0.0003	0.00551994
BHLHE22	chr8:65492794-65496191	20.8418	3.35065	-2.63696	-3.46977	0.00005	0.00136643
MYPN	chr10:69865873-69971773	0.253468	0.0404669	-2.64699	-1.5601	0.00085	0.0120733
CCL19	chr9:34689566-34691274	1.31025	0.208588	-2.65112	-1.5843	0.003	0.0299609
DCDC2	chr6:24171982-24383520	6.46602	1.02787	-2.65323	-2.60921	0.00005	0.00136643
SPATA18	chr4:52917592-52963458	6.36845	0.993625	-2.68017	-2.79968	0.00005	0.00136643
SLC30A3	chr2:27477439-27485960	27.0996	4.21619	-2.68426	-3.21268	0.00005	0.00136643
ABCC12	chr16:48116883-48180681	0.812494	0.126343	-2.68501	-2.27267	0.00005	0.00136643
ST8SIA2	chr15:92937139-93011958	1.06547	0.164016	-2.69958	-2.26959	0.00005	0.00136643
TGM3	chr20:2276612-2321725	0.844688	0.129699	-2.70325	-2.05875	0.00005	0.00136643
FAM216B	chr13:43355685-43365685	5.36657	0.821832	-2.70709	-2.41044	0.00005	0.00136643
FIBCD1	chr9:133777824-133814455	15.984	2.43344	-2.71556	-3.17337	0.00005	0.00136643
PDGFD	chr11:103777913-104035027	4.83987	0.731781	-2.72548	-2.45324	0.00005	0.00136643
FCN3	chr1:27695600-27701315	2.27991	0.341272	-2.73999	-1.60819	0.0003	0.00551994
MAK	chr6:10762955-10838788	1.54358	0.22942	-2.75022	-2.21929	0.00005	0.00136643
SLC39A12	chr10:18240767-18332221	155.959	23.1584	-2.75157	-1.88307	0.0022	0.0239231
KCNA5	chr12:5153084-5155954	15.6944	2.31595	-2.76058	-2.75061	0.00005	0.00136643
FAM46C	chr1:118148603-118171011	6.15348	0.905291	-2.76495	-2.32333	0.00005	0.00136643
C1orf158	chr1:12806133-12821102	1.97546	0.289342	-2.77134	-1.7177	0.00115	0.0149934
FAM81B	chr5:94727047-94786144	4.90503	0.717655	-2.7729	-2.17349	0.00005	0.00136643
VWA5B1	chr1:20617411-20681387	2.43533	0.354749	-2.77924	-2.34304	0.00005	0.00136643
CLEC4G	chr19:7793842-7797057	3.18769	0.463368	-2.78228	-2.24594	0.00005	0.00136643
SST	chr3:187386693-187388201	37.9111	5.46577	-2.79412	-2.90783	0.00005	0.00136643
PZP	chr12:9301435-9360966	1.70655	0.245298	-2.79848	-1.79308	0.0031	0.0306643

WIF1	chr12:65444403-65515346	22.1415	3.14939	-2.81361	-3.19923	0.00005	0.00136643
FAM92B	chr16:85131964-85146114	2.1274	0.300886	-2.8218	-1.97675	0.0003	0.00551994
CELSR1	chr22:46756730-46933067	1.23788	0.174748	-2.82452	-2.5292	0.00005	0.00136643
ANKUB1	chr3:149478889-149510610	2.28143	0.318615	-2.84005	-1.83223	0.00085	0.0120733
MS4A8	chr11:60467046-60483285	1.76856	0.246529	-2.84275	-1.7057	0.00035	0.00620045
C1orf168	chr1:57184476-57285369	3.06531	0.426209	-2.8464	-2.14448	0.00005	0.00136643
LOC10050753	chr16:47883224-47942342	1.40493	0.194356	-2.85373	-1.83657	0.002	0.0224405
LOC10192815	chr5:173213792-173217945	2.31987	0.309559	-2.90576	-1.75158	0.00055	0.00876569
CRH	chr8:67088611-67090846	1.06026	0.138184	-2.93976	-1.73923	0.0024	0.0253778
PIP5K1B	chr9:71320329-71624092	35.2183	4.5729	-2.94514	-2.59994	0.0001	0.00238525
BCMO1	chr16:81272295-81324747	4.49825	0.579657	-2.95609	-2.35962	0.00005	0.00136643
C18orf42	chr18:5143671-5197255	2.9572	0.372945	-2.9872	-2.45328	0.00005	0.00136643
LINC00624	chr1:146853913-146989699	0.296394	0.0373341	-2.98895	-1.48335	0.00105	0.0140962
TRIM29	chr11:119981993-120008863	1.0356	0.128976	-3.00529	-2.14078	0.00005	0.00136643
COL5A2	chr2:189896640-190044605	19.6076	2.41883	-3.01904	-3.59836	0.00005	0.00136643
DCDC5	chr11:30885149-31014233	3.29093	0.405971	-3.01905	-3.11796	0.00005	0.00136643
RARRES1	chr3:158414896-158450275	3.93599	0.481839	-3.0301	-1.96994	0.0002	0.00407561
FLJ41200	chr9:13406378-13431328	1.23979	0.150518	-3.0421	-2.14187	0.00005	0.00136643
C9orf171	chr9:135285582-135448706	1.78075	0.213969	-3.05701	-2.03813	0.00085	0.0120733
PROS1	chr3:93591880-93692934	41.6167	4.93706	-3.07544	-2.74108	0.00005	0.00136643
ATP13A5	chr3:192992830-193096514	6.3031	0.737017	-3.09629	-2.39628	0.00005	0.00136643
DNAI2	chr17:72270385-72311023	1.83236	0.213452	-3.10172	-2.11018	0.0001	0.00238525
PLVAP	chr19:17462263-17488137	4.33026	0.502291	-3.10786	-2.26565	0.00005	0.00136643
ANKRD66	chr6:46714653-46726954	2.35635	0.272046	-3.11463	-1.87708	0.00005	0.00136643
C9orf135	chr9:72435730-72521148	4.6649	0.52214	-3.15934	-1.92915	0.0001	0.00238525
RGS9BP	chr19:33166312-33169206	9.31758	1.03528	-3.16994	-2.57055	0.00005	0.00136643
FAM183A	chr1:43613593-43622067	7.43283	0.819049	-3.18189	-2.15635	0.00005	0.00136643
SFRP1	chr8:41119475-41166990	88.302	9.67682	-3.18984	-2.30391	0.00005	0.00136643
HTR2C	chrX:113818550-114144624	36.8688	4.0156	-3.19871	-2.72654	0.00005	0.00136643
MICALCL	chr11:12308446-12380691	1.30475	0.140827	-3.21178	-2.32449	0.00005	0.00136643
SGMS2	chr4:108745720-108836204	12.4795	1.34696	-3.21178	-2.89607	0.00005	0.00136643
CA12	chr15:63615729-63674075	22.0863	2.26858	-3.28329	-2.64322	0.00005	0.00136643
SPAG6	chr10:22634373-22706539	11.4378	1.16182	-3.29935	-2.90525	0.00005	0.00136643
CPXM2	chr10:125505151-125651500	13.6626	1.38187	-3.30554	-2.76137	0.00005	0.00136643
CCDC33	chr15:74528629-74628482	2.35803	0.236737	-3.31622	-2.12908	0.00585	0.0490104
HPCA	chr1:33352097-33366953	225.082	22.4233	-3.32738	-4.27152	0.00005	0.00136643
C11orf88	chr11:111385509-111407756	9.0855	0.904167	-3.32891	-2.31295	0.00005	0.00136643
GPX3	chr5:150399998-150408554	125.611	12.3362	-3.34799	-2.52238	0.0002	0.00407561
IGJ	chr4:71521257-71532348	1.51982	0.149179	-3.34879	-1.91625	0.0011	0.0145132
TFCP2L1	chr2:121974163-122042778	5.29582	0.518698	-3.35189	-2.91346	0.00005	0.00136643
TAC1	chr7:97361270-97369784	14.9066	1.45482	-3.35703	-3.30517	0.00005	0.00136643
KCNE1	chr21:35818985-35884573	2.83118	0.275903	-3.35917	-2.82036	0.00005	0.00136643
FAM3B	chr21:42688660-42729654	0.566109	0.0547605	-3.36987	-1.58097	0.00065	0.00986869
F5	chr1:169481191-169555769	14.2987	1.35666	-3.39776	-2.20628	0.00015	0.00330506
LAG3	chr12:6881669-6887621	1.08041	0.101909	-3.40623	-1.79247	0.00115	0.0149934
SOSTDC1	chr7:16501105-16505474	9.46238	0.884447	-3.41936	-2.69431	0.00005	0.00136643
DCSTAMP	chr8:105352023-105368917	4.13129	0.380261	-3.44153	-2.50286	0.0044	0.039835
LOC643711	chr12:98107189-98150295	0.824421	0.0758612	-3.44195	-1.95439	0.0011	0.0145132

LINC00996	chr7:150130741-150145228	0.415101	0.0376827	-3.46149	-1.87688	0.00135	0.0168292
CLDN4	chr7:73245192-73247023	2.76203	0.238591	-3.53312	-1.88877	0.0003	0.00551994
SOD3	chr4:24797084-24802467	25.0211	2.11069	-3.56736	-2.95857	0.00005	0.00136643
KRT18	chr12:53290970-53346685	11.7086	0.966963	-3.59796	-2.45172	0.00045	0.00751151
DSG2	chr18:29078026-29136874	6.01001	0.486559	-3.62668	-2.53732	0.00005	0.00136643
KLHDC7A	chr1:18807423-18812480	1.12955	0.0911005	-3.63215	-2.00705	0.00005	0.00136643
C4orf22	chr4:81256873-81884910	4.70361	0.372629	-3.65795	-2.50581	0.0001	0.00238525
LIPH	chr3:185225569-185270369	1.54883	0.122026	-3.66592	-2.07256	0.00035	0.00620045
SCGN	chr6:25652428-25702008	4.5291	0.353833	-3.67808	-2.84672	0.00005	0.00136643
PENK	chr8:57353512-57359282	19.2112	1.49109	-3.68751	-3.16206	0.00005	0.00136643
AIM1	chr6:106959729-107018334	9.68344	0.74603	-3.69821	-3.09511	0.00005	0.00136643
ROR2	chr9:94484877-94712444	6.02268	0.463522	-3.6997	-3.37485	0.00005	0.00136643
DNAH11	chr7:21582832-21985542	7.94324	0.605833	-3.71273	-2.99637	0.00005	0.00136643
PRSS8	chr16:31142753-31147151	0.75915	0.0576808	-3.71822	-1.94541	0.00075	0.0109258
CDHR4	chr3:49828166-49837254	0.440722	0.0327966	-3.74825	-1.98057	0.00015	0.00330506
DUSP4	chr8:29190578-29208267	12.0142	0.893125	-3.74974	-4.67471	0.00005	0.00136643
SLC5A5	chr19:17982781-18005983	15.074	1.11077	-3.76242	-2.29207	0.00005	0.00136643
NEUROD6	chr7:31377074-31380538	34.0121	2.48862	-3.77263	-4.47051	0.00005	0.00136643
LRRC71	chr1:156890423-156902880	1.50275	0.106849	-3.81396	-2.42915	0.00005	0.00136643
RIPK4	chr21:43159528-43187249	0.888884	0.0624148	-3.83203	-1.91008	0.00035	0.00620045
TTLL10	chr1:1109285-1133313	0.624215	0.0419912	-3.89388	-2.43826	0.00025	0.00483727
TMEM27	chrX:15645438-15683154	3.96007	0.266396	-3.89388	-2.23777	0.0001	0.00238525
SLC16A12	chr10:91190050-91295313	23.9616	1.61112	-3.89459	-3.24943	0.00005	0.00136643
CCDC60	chr12:119772516-119978852	2.09976	0.135519	-3.95366	-2.40747	0.00005	0.00136643
NXNL1	chr19:17566233-17571725	5.12725	0.330187	-3.95683	-2.11348	0.0003	0.00551994
TDO2	chr4:156824844-156841558	1.18704	0.0753667	-3.9773	-2.36964	0.0003	0.00551994
C6orf223	chr6:43968336-43973694	0.605649	0.0375488	-4.01164	-2.03054	0.00005	0.00136643
NR1H4	chr12:100867550-100957645	0.277928	0.0167103	-4.0559	-1.32969	0.0026	0.0269589
RBP1	chr3:139236275-139258671	129.966	7.72847	-4.07181	-3.65265	0.00005	0.00136643
DMRT3	chr9:976967-991732	2.10988	0.12374	-4.09178	-2.52058	0.00005	0.00136643
RAB17	chr2:238482964-238499769	4.74692	0.274508	-4.11207	-3.07211	0.00005	0.00136643
TMPRSS2	chr21:42836477-42880085	0.619173	0.0353163	-4.13194	-2.26659	0.00005	0.00136643
LEFTY2	chr1:226124297-226129083	1.91356	0.0909768	-4.39462	-2.7553	0.00005	0.00136643
PRND	chr20:4702499-4709108	1.6517	0.0721438	-4.51693	-3.21348	0.00005	0.00136643
FAM26D	chr6:116817650-116880031	0.343638	0.0143116	-4.58563	-0.508764	0.0042	0.0384589
LINC00880	chr3:156799455-156840791	5.46293	0.220155	-4.63308	-3.19967	0.00005	0.00136643
ADRA1D	chr20:4201277-4229659	6.98026	0.279548	-4.64211	-4.18817	0.00005	0.00136643
TNNC1	chr3:52485106-52488057	4.17917	0.157945	-4.72572	-2.32568	0.0001	0.00238525
C2orf40	chr2:106682112-106694609	82.5225	2.94826	-4.80685	-3.91916	0.00005	0.00136643
TMC5	chr16:19422056-19510434	2.70748	0.0965195	-4.80999	-3.45327	0.00005	0.00136643
SERPINF1	chr17:1665258-1680859	277.495	9.32355	-4.89544	-3.38852	0.00005	0.00136643
SELP	chr1:169558087-169599377	4.94479	0.15968	-4.95266	-2.89769	0.00005	0.00136643
GPR1	chr2:207040041-207130967	3.9334	0.120249	-5.03168	-2.8538	0.00005	0.00136643
RASSF6	chr4:74437266-74486348	0.661687	0.0187366	-5.14222	-2.57271	0.00325	0.0317912
GALNT5	chr2:158114339-158167913	1.38677	0.036895	-5.23216	-3.00033	0.00005	0.00136643
SFRP5	chr10:99526507-99531756	9.39072	0.229022	-5.35768	-3.20997	0.00005	0.00136643
LOC200772	chr2:241894035-241906868	2.77876	0.0673559	-5.3665	-2.98531	0.00005	0.00136643
FNDC1	chr6:159590428-159693140	6.22773	0.133012	-5.54907	-5.09316	0.00005	0.00136643

GDF15	chr19:18496967-18499986	29.0671	0.614337	-5.56421	-4.1267	0.00005	0.00136643
CNGB3	chr8:87586162-87755903	0.362716	0.00724745	-5.64522	-2.46513	0.00425	0.0387958
SLC28A3	chr9:86890764-86983413	2.40013	0.0478423	-5.64868	-2.97526	0.00005	0.00136643
TRPV4	chr12:110220891-110271241	7.56669	0.14677	-5.68804	-3.19795	0.00005	0.00136643
CLDN3	chr7:73183326-73184600	3.06963	0.0486334	-5.97998	-3.20122	0.00115	0.0149934
ABCA4	chr1:94458393-94586705	15.4059	0.207415	-6.21482	-3.67632	0.00005	0.00136643
PRLR	chr5:35048860-35230691	99.6137	1.00133	-6.63635	-2.25201	0.00005	0.00136643
KCNJ13	chr2:233562014-233725289	44.3137	0.409328	-6.75835	-1.41362	0.00345	0.0333771
GYLTL1B	chr11:45943195-45950647	1.52911	0.0137681	-6.79522	-3.04922	0.00435	0.0394838
FOLR1	chr11:71900601-71907367	81.6281	0.668816	-6.93131	-4.13698	0.0007	0.010395
CLIC6	chr21:36041687-36090519	80.1928	0.63859	-6.97244	-4.44714	0.00005	0.00136643
FABP4	chr8:82390731-82395473	33.9285	0.267781	-6.9853	-4.05899	0.00005	0.00136643
CDHR5	chr11:616564-625067	2.09731	0.0130138	-7.33235	-3.45748	0.00225	0.024287
TYRP1	chr9:12693385-12710266	12.2193	0.0748897	-7.35018	-4.61704	0.00005	0.00136643
LMX1A	chr1:165171103-165325952	2.20189	0.0122358	-7.49149	-3.59069	0.0006	0.00935947
SCNN1A	chr12:6456008-6500737	13.9851	0.0477926	-8.19289	-1.53	0.0008	0.0115682
OTX2	chr14:57267424-57277194	15.432	0.0435214	-8.46999	-4.10906	0.00105	0.0140962
HPD	chr12:122277432-122326517	36.3974	0.0998204	-8.51028	-5.06336	0.0001	0.00238525
KRT5	chr12:52908358-52914243	20.6261	0.0490027	-8.7174	-5.175	0.00005	0.00136643
FBP2	chr9:97320995-97356114	22.1949	0.0379637	-9.19139	-5.0293	0.0014	0.0173787
CLDN2	chrX:106143292-106174091	23.431	0.0251896	-9.86137	-4.77078	0.00045	0.00751151

Supplementary Table S2. Protein coding genes resulted deregulated from RNA-Seq data analysis of the hippocampal region of LOAD patients compared to controls. Expression levels are expressed as Log2 fold change; the P-value and the corresponding corrected P-value were calculated by CuffDiff2.

Legend
Gene Symbol: Gene Symbol from RefSeq annotations
Locus: Genomic locus according to hg19 human genome assembly
value_Ctrl: Mean expression level (in FPKM) per Control group calculated by CuffDiff2
value_PD: Mean expression level (in FPKM) per PD group calculated by CuffDiff2
log2(fold_change): Log2 of the ratio value_Ctrl/value_PD calculated by CuffDiff2
test_stat: Statistical Test value calculated by CuffDiff2
p_value: P value calculated by CuffDiff2
q_value: Corrected P value calculated by CuffDiff2

**Gene Symbol**	**Locus**	**value_Ctrl**	**value_PD**	**log2(fold_change)**	**test_stat**	**p_value**	**q_value**
HLA-DRA	chr6_ssto_hap7:3754282-3759493	0.226689	9.69449	5.41838	3.27568	5,00E-05	0.0454421
HSPA6	chr1:161494035-161496687	0.603785	18.3459	4.92528	2.99514	5,00E-05	0.0454421
CHRNA2	chr8:27317277-27336813	0.204193	5.18865	4.66736	2.78274	5,00E-05	0.0454421
NPAS4	chr11:66188474-66194177	0.211207	3.44844	4.02921	2.09023	5,00E-05	0.0454421
SFN	chr1:27189632-27190947	0.388187	5.55984	3.84022	2.11454	5,00E-05	0.0454421
CXCL8	chr4:74606222-74609433	1.19927	16.3187	3.76629	2.1111	5,00E-05	0.0454421
HAMP	chr19:35773409-35776045	1.15162	15.3527	3.73675	1.99263	5,00E-05	0.0454421
PVALB	chr22:37196744-37215517	2.00308	25.0226	3.64294	2.03643	5,00E-05	0.0454421
ANKRD22	chr10:90562486-90611732	0.0546895	0.63663	3.54112	1.82818	5,00E-05	0.0454421
FOSL1	chr11:65659691-65667997	0.159539	1.44116	3.17525	1.54369	5,00E-05	0.0454421
HSPA7	chr1:161575848-161578341	0.836662	6.20282	2.89021	2.00927	5,00E-05	0.0454421
SYT2	chr1:202559724-202679551	0.549678	3.29916	2.58544	1.82539	5,00E-05	0.0454421
SERPINH1	chr11:75273100-75283849	5.53594	23.4339	2.0817	1.85284	5,00E-05	0.0454421
CD14	chr5:140011312-140013286	7.97265	32.3621	2.02117	1.93879	5,00E-05	0.0454421
TNFRSF1B	chr1:12226999-12269277	3.58355	13.3321	1.89544	1.77902	5,00E-05	0.0454421
HSPB1	chr7:75931874-75933614	32.0892	115.061	1.84224	1.85775	5,00E-05	0.0454421
BAG3	chr10:121410881-121437329	12.517	42.8248	1.77455	1.74554	5,00E-05	0.0454421
FCGBP	chr19:40353962-40440533	1.74626	5.69364	1.70509	1.80527	5,00E-05	0.0454421
SELP	chr1:169558087-169599377	4.06267	0.329058	-3.62601	-1.81915	5,00E-05	0.0454421

Supplementary Table S3. Protein coding genes resulted deregulated from RNA-Seq data analysis of the hippocampal region of PD patients compared to controls. Expression levels are expressed as Log2 fold change; the P-value and the corresponding corrected P-value were calculated by CuffDiff2.

Case	Case ID	Gender	Race	Expired age	PMI (hrs)	Braak stage	Brain Bank	RIN
Crtl7	A213/12	male	Ca	78	24	ND	LNDBB	4.2
Crtl8	A319/11	male	Ca	74	22	ND	LNDBB	4.6
Crtl10	A114/12	male	Ca	82	24	ND	LNDBB	2.7
Crtl11	PDC022	male	Ca	75	ND	ND	PUKBB	4.9
AD8	A283/09	male	Ca	77	10	VI	LNDBB	4.7
AD9	A341/11	male	Ca	76	8	VI	LNDBB	5.9
AD10	A318/09	male	Ca	72	5	VI	LNDBB	5.9
AD11	A111/12	male	Ca	70	20	VI	LNDBB	6.3

Supplementary Table S4. Additional samples selected for RT-qPCR experiments. Case: sample ID assigned in the present study; Case ID: sample ID in the original Bank; Ctrl: Non-Demented Control; AD: Late onset Alzheimer's Disease Patient; Race: Ca= Caucasian; PMI: Post-Mortem Interval expressed in hours; Braak stage: index used to classify the degree of AD pathology; ND: Not defined; LNDBB: London Neurodegenerative Diseases Brain Bank; PUKBB: Parkinson's UK Brain Bank; RIN: RNA integrity number.

Gene/lncRNA	Description	RNA-Seq log2(FC)	TaqMan® Gene Expression Assays ID
CPLX3	Complexin 3	1.27713	Hs00226740_m1
NR4A2	Nuclear receptor subfamily 4 group A member 2	1.34818	Hs00428691_m1
GRIK3	Glutamate ionotropic receptor kainate type subunit 3	1.2135	Hs00168182_m1
TESPA1	Thymocyte expressed, positive selection associated 1	4.44102	Hs00207702_m1
SLCO4A1	Solute carrier organic anion transporter family member 4A1	3.15842	Hs00983988_m1
SERPINA5	Serpin family A member 5	2.64909	Hs04333915_m1
ADAM33	ADAM metallopeptidase domain 33	2.39175	Hs00905552_m1
SERPINA1	Serpin family A member 1	2.51271	Hs00165475_m1
BHLHE22	Basic helix-loop-helix family member e22	-2.63696	Hs01084964_s1
PRSS12	Protease, serine 12	-2.46718	Hs00186221_m1
NEUROD6	Neuronal differentiation 6	-3.77263	Hs00745618_s1
NEUROD1	Neuronal differentiation 1	-2.23346	Hs00159598_m1
PCDH8	Protocadherin 8	-1.98189	Hs04187285_g1
SCN11A	Sodium voltage-gated channel alpha subunit 11	-1.68122	Hs00204222_m1
ARC	Activity regulated cytoskeleton associated protein	-1.92259	Hs01045540_g1
PRKCG	Protein kinase C gamma	-2.15096	Hs00177010_m1
GRIA1	Glutamate ionotropic receptor AMPA type subunit 1	-1.88396	Hs00181348_m1
NRN1	Neuritin 1	-1.84677	Hs00213192_m1
DUSP4	Dual specificity phosphatase 4	-3.74974	Hs01027785_m1
SYTL5	Synaptotagmin like 5	-2.52327	Hs00371091_m1
CAMK1D	Calcium/calmodulin dependent protein kinase ID	-1.4763	Hs00220668_m1
LOC400891	Long noncoding RNA LOC400891	-2.24346	Hs00416319_m1

Supplementary Table S5. Differentially expressed coding and non coding genes resulting from hippocampal RNA-Seq data analysis and TaqMan® Gene Expression Assays used for RT-qPCR validation.

CaseID	Sample	Brain Areas	PF Reads
5028	Ctrl1	HIP	5,594,716
		GTM	6,062,202
		GFM	3,508,636
5174	Ctrl2	HIP	4,365,193
		GTM	4,172,119
		GFM	5,321,332
5247	Ctrl3	HIP	4,866,579
		GTM	5,020,766
		GFM	3,283,898
5352	Ctrl4	HIP	4,895,164
		GTM	5,066,063
		GFM	3,496,835
5533	Ctrl5	HIP	4,233,352
		GTM	4,818,247
		GFM	3,916,527
5362	Ctrl6	HIP	4,920,853
		GTM	5,098,064
		GFM	3,977,584
1625	AD1	HIP	4,401,114
		GTM	4,164,340
		GFM	3,373,129
4737	AD2	HIP	5,110,156
		GTM	7,765,437
		GFM	3,519,459
5195	AD3	HIP	3,905,615
		GTM	5,045,195
		GFM	3,336,492
5198	AD4	HIP	3,988,204
		GTM	4,125,095
		GFM	4,492,951
1946	AD5	HIP	5,012,121
		GTM	5,543,402
		GFM	3,303,003
3136	AD6	HIP	5,411,173
		GTM	5,485,136
		GFM	4,987,147
1272	PD1	HIP	4,334,166
1741	PD2	HIP	2,792,271
1901	PD3	HIP	3,771,754
4526	PD4	HIP	9,078,887
5329	PD5	HIP	3,335,528
5520	PD6	HIP	3,519,065

Supplementary Table S6. Statistics of miRNA-seq data in hippocampus, middle temporal and frontal gyrus. HIP: hippocampus; GTM: middle temporal gyrus; GFM: middle frontal gyrus; PF reads: Passing Filter reads assigned to an index.

miRNA	log2FC	p-value	padj
hsa-miR-146b-5p	2.53	2.10E-09	7.49E-08
hsa-miR-146a-5p	2.41	7.30E-17	2.38E-14
hsa-miR-24-3p	2.05	3.90E-12	4.17E-10
hsa-miR-30a-5p	1.78	2.40E-05	4.38E-04
hsa-miR-1307-5p	1.66	6.00E-04	6.55E-03
hsa-miR-33a-5p	1.64	6.00E-05	9.24E-04
hsa-miR-32-5p	1.51	4.70E-04	5.39E-03
hsa-miR-192-5p	1.42	2.00E-09	7.49E-08
hsa-miR-151b	1.27	2.20E-10	1.18E-08
hsa-miR-126-5p	0.99	1.80E-03	1.51E-02
hsa-miR-191-5p	0.71	1.90E-04	2.35E-03
hsa-miR-1271-5p	-2.94	1.50E-10	9.98E-09
hsa-miR-501-3p	-2.88	2.00E-16	3.28E-14
hsa-miR-409-5p	-2.75	2.10E-07	5.64E-06
hsa-miR-193a-3p	-2.53	1.50E-04	1.93E-03
hsa-miR-149-5p	-2.08	3.20E-11	2.62E-09
hsa-miR-129-1-3p	-1.92	1.50E-03	1.38E-02
hsa-miR-3607-3p	-1.92	8.90E-08	2.63E-06
hsa-miR-1249-3p	-1.86	5.10E-08	1.66E-06
hsa-miR-412-5p	-1.82	1.60E-03	1.39E-02
hsa-miR-138-5p	-1.77	3.10E-05	5.13E-04
hsa-miR-551b-3p	-1.74	1.60E-03	1.39E-02
hsa-miR-935	-1.62	8.60E-04	9.05E-03
hsa-miR-331-3p	-1.52	8.40E-03	4.96E-02
hsa-miR-150-5p	-1.52	2.90E-10	1.36E-08
hsa-miR-504-5p	-1.51	7.60E-03	4.57E-02
hsa-miR-885-5p	-1.47	3.00E-06	7.10E-05
hsa-miR-497-5p	-1.37	1.10E-05	2.29E-04
hsa-let-7e-3p	-1.31	1.20E-04	1.59E-03
hsa-miR-128-3p	-1.28	2.10E-03	1.64E-02
hsa-miR-664a-3p	-1.27	3.20E-03	2.33E-02
hsa-let-7d-3p	-1.09	1.80E-05	3.48E-04
hsa-miR-197-3p	-1.04	6.00E-03	3.88E-02
hsa-miR-361-3p	-0.92	3.20E-05	5.13E-04
hsa-miR-328-3p	-0.88	5.20E-03	3.53E-02
hsa-miR-365b-3p	-0.85	2.90E-03	2.14E-02
hsa-miR-484	-0.76	1.60E-05	3.34E-04
hsa-miR-125a-5p	-0.67	7.20E-05	1.07E-03
hsa-miR-29c-3p	-0.59	5.50E-03	3.65E-02
hsa-miR-423-3p	-0.57	6.90E-03	4.30E-02

Supplementary Table S7. miRNAs resulted deregulated from miRNA-Seq data analysis of the hippocampal region CA1 of PD patients compared to controls. Expression levels are expressed as log2 fold change; the P-value and the corresponding corrected P-value (padj) were calculated by DESeq.

KEGG Pathway	p-value
Neurotrophin signaling pathway	2.40E-31
MAPK signaling pathway	2.30E-30
Long-term potentiation	9.40E-17
Dopaminergic synapse	1.30E-09
Wnt signaling pathway	1.10E-07
Axon guidance	9.60E-07
B cell receptor signaling pathway	2.10E-06
HIF-1 signaling pathway	2.40E-06
T cell receptor signaling pathway	8.20E-06
Calcium signaling pathway	1.20E-05
Cholinergic synapse	5.10E-05
Chemokine signaling pathway	5.70E-05
Retrograde endocannabinoid signaling	6.20E-05
Notch signaling pathway	8.80E-05
Glutamatergic synapse	9.80E-05
Apoptosis	4.70E-04
Fc gamma R-mediated phagocytosis	1.70E-03
mTOR signaling pathway	5.80E-03
Long-term depression	7.90E-03

Supplementary Table S8. KEGG Pathways affected by PD deregulated miRNAs in the hippocampus. DIANA miRPath analysis identified several pathways involved in neuronal and inflammatory processes. The Fisher-exact test P-value is reported in each column.

miRNA	Brain Areas	miRNA-Seq log2(FC)	TaqMan® Advanced miRNA Assays ID
hsa-miR-320a	GTM	0.76	478594_mir
hsa-miR-10a-5p	GTM	1.23	479241_mir
hsa-miR-30a-3p	GTM	0.51	478273_mir
hsa-miR-28-3p	GTM	0.61	477999_mir
hsa-miR-501-3p	GTM	2.13	478350_mir
hsa-miR-539-5p	GTM	-1.53	478152_mir
hsa-miR-184	HIP	-4.26	477938_mir
hsa-miR-34c-3p	HIP	-3.31	478051_mir
hsa-miR-375	HIP	-1.77	478074_mir
hsa-miR-941	GFM	0.80	479217_mir
hsa-miR-889-3p	GFM	-1.27	478208_mir
hsa-miR-582-5p	GFM	-0.85	478166_mir
hsa-miR-132-3p	GTM<br>GFM	-1.85<br>-1.58	478705_mir
hsa-miR-132-5p	HIP<br>GTM<br>GFM	-1.04<br>-1.77<br>-0.91	477900_mir
hsa-miR-212-3p	GTM<br>GFM	-1.80<br>-1.99	478318_mir
hsa-miR-212-5p	GTM<br>GFM	-1.85<br>-1.34	478767_mir

Supplementary Table S9. MiRNAs and TaqMan® Advanced miRNA Assays selected for RT-qPCR validation of miRNA-seq data analysis in hippocampus, middle temporal and frontal gyrus. Expression levels, expressed as log2 fold change, are indicated.

HIP: hippocampus; GTM: middle temporal gyrus; GFM: middle frontal gyrus.
